# Miktoarm Star-polypept(o)ide-Based Polyion Complex Micelles for the Delivery of Large Nucleic Acids

**DOI:** 10.1021/acs.biomac.4c00695

**Published:** 2024-09-18

**Authors:** David Schwiertz, Jennifer Angelina, Heyang Zhang, Matthias Barz

**Affiliations:** † Biotherapeutics Division, Leiden Academic Centre for Drug Research (LACDR), Leiden University, Einsteinweg 55, 2333 CC Leiden, The Netherlands; ‡ Department of Dermatology, University Medical Center, Johannes Gutenberg-University Mainz (JGU), Obere Zahlbacher Straße 63, 55131 Mainz, Germany

## Abstract

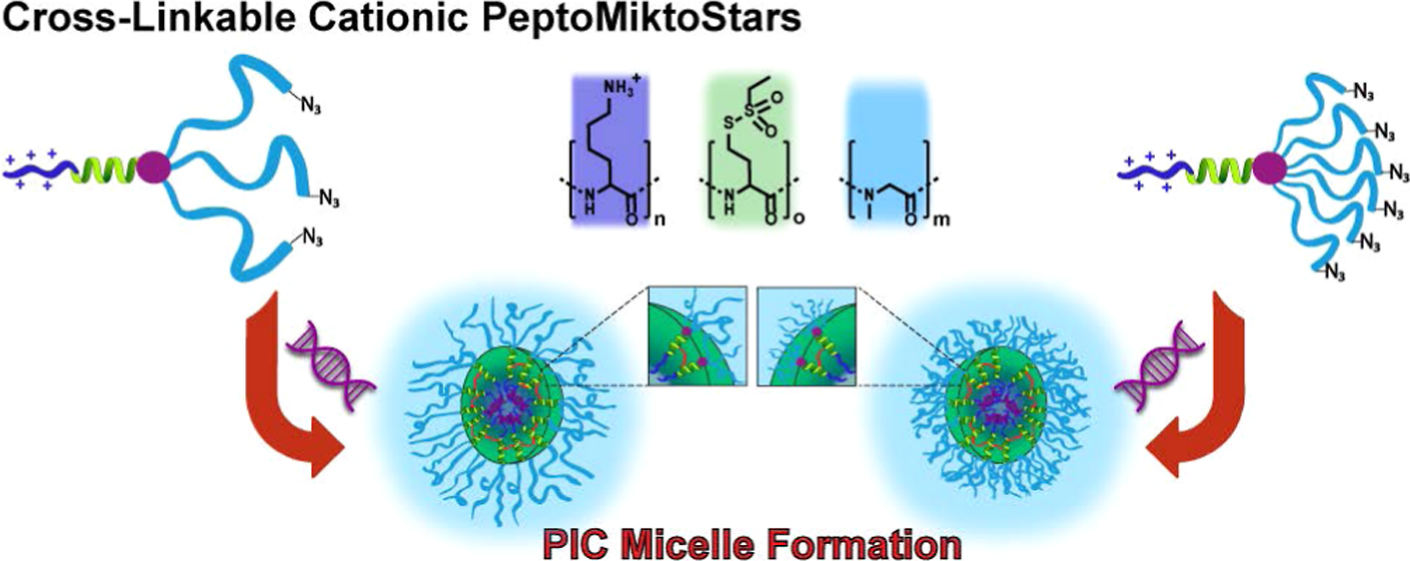

Miktoarm star polymers exhibit a captivating range of physicochemical properties, setting them apart from their linear counterparts. This study devised a synthetic pathway to synthesize cationic miktoarm stars utilizing polypept(o)ides (PeptoMiktoStars), comprising 3 or 6 polysarcosine (pSar) arms (AB_3×100_, AB_6×50_, overall 300) for shielding and a cross-linkable poly(*S*-ethylsulfonyl-l-homocysteine) (pHcy(SO_2_Et)_20_) block and a poly(l-lysine) ((pLys)_20_) block for nucleic acid complexation. Precise control over the DP_*n*_ and narrow molecular weight distributions (*D̵* ≈ 1.2) were achieved for both structures. Both PeptoMiktoStars efficiently complexed mRNA and pDNA into polyion complex micelles (PICMs). AB_6_–PICMs provided modest (mRNA) to high (pDNA) stability against glutathione and heparin sulfate (HS), while even cross-linked AB_3_–PICMs were susceptible to HS. All PICMs delivered pDNA and mRNA into D1 cells (over 80%) and Jurkat T cells (over 50%) in vitro. Despite payload- and cell-dependency, AB_3_ showed overall higher transfection efficiency, while AB_6_ demonstrated better shielding and enhanced stability.

## 1 Introduction

Polypept(o)ides are a class of hybrid materials combining polypeptoids, e.g., polysarcosine (pSar), and polypeptides.^1,2^ These polymers are readily accessible by sequentially controlled living ring-opening polymerization (ROP) of the corresponding *N*-carboxyanhydrides (NCAs), resulting in well-defined polymers with low dispersity, high end group integrity, and precise control over chain length and block-sequence.^3,4^ The polypeptoid pSar has been identified as one of the most promising alternatives to polyethylene glycol (PEG) since it possesses comparable solution properties, namely second virial coefficient and Kuhn-length, and has demonstrated a stealth-like nature, namely absence of a protein corona and long blood half-life time in zebrafish, mice, and rats upon intravenous or intraperitoneal injection.^5−9^ Interestingly, pSar displays reduced immunogenicity compared to PEG.^10−16^ In addition to lipid formulations, pSar has been involved in several polymeric drug delivery systems to enhance stability, reduce toxicity, off-target effect, and improve therapeutic efficacy.^17^ Our group recently introduced novel ABC-type triblock copolypept(o)ides constructed as a combination of a shielding pSar block, a cross-linkable poly(*S*-ethylsulfonyl-l-cysteine) block (pCys(SO_2_Et)), and a polycationic poly(l-lysine) block (pLys), which successfully formed polyion complex micelles (PICMs), encapsulated, and delivered small interfering RNA (siRNA) into Neuro2A and KB cells.^18^

To further improve the shielding of nucleic acids, an application of miktoarm star polymers seems beneficial. Miktoarm star polymers represent a class of star polymers featured by an asymmetrical branching structure with at least three polymer arms emanating from a central core.^19^ The polymer architecture of such polymers is depicted in the form of A_*X*_B_*Y*_, where A and B denote the polymeric chains, and the subscript indicates the quantity of the respective arm.^20^ The asymmetrical topology of miktoarm star polymers has given rise to unique characteristics for their aqueous self-assemblies, such as a lower critical micelle concentration, increased internal and peripheral functionalities, and a higher polymer density at the block interface for better shielding (multiple stealth-like blocks) or improved encapsulation capabilities (multiple cationic blocks).^21,22^ Additionally, during the synthesis of the respective miktoarm stars, both the surface and the interior of the resulting nanocarriers, such as PICMs, can be tuned, which enables better control over morphology, improved blood circulation half-life, and controlled drug release.^23−25^

Star polymers have been already exploited as vehicles for nucleic acids, peptides, and drugs.^26^ For instance, symmetrical 3-arm and 6-arm star diblock copolymers of pLys-*b*-pSar have demonstrated their potential as building blocks in PICMs for siRNA delivery, with high complexation capacity and efficient siRNA release in the presence of increased glutathione concentrations.^27^ In addition to small nucleic acid delivery, Huh et al. have shown an improved transfection efficiency while maintaining low cytotoxicity by using the star polymer of PLL–PEG block for pDNA delivery.^28^ Chen et al. reported a reduced pDNA condensation but overall a higher transfection efficiency using cyclodextrin (CD)-cored star-shaped poly(2-aminoethyl methacrylate) (PAEM) compared with the linear PAEM-based polyplexes.^29^ In 2020, a star polymer of poly(*N*,*N*′-dimethylaminoethyl methacrylate)-*b*-poly(oligo(ethylene glycol) methacrylate) (pDMAEMA-*b*-pOEGMA-OH) was first explored for mRNA delivery, which however did not show potent transfection efficiency.^30^ Despite these interesting properties, the realization of well-defined asymmetrical structures with multiple well-defined designed arms remains chemically challenging, owing to the rigorous demands of the synthesis of the individual blocks and purification processes associated with the applied polymerization strategies.^31−33^ Typically, miktoarm star polymers are synthesized using either the arm-first or core-first synthetic approaches, often in conjunction with orthogonal initiators for the combination of at least two polymerization techniques, multiple protection/deprotection steps, or by connecting presynthesized polymers.^34−37^ Although common approaches can yield high molecular weights, it is widely acknowledged that the dispersity can broaden undesirably due to the side reactions such as star–star coupling, resulting in poorly defined polymers, and translation to specific biomedical materials makes the process even more challenging.^38^

In this study, we report on the development of core cross-linkable AB_3_ and AB_6_ miktoarm star polymers based on polypept(o)ides (PeptoMiktoStars). The asymmetric architecture consists of one arm composed of a block poly(l-lysine) (pLys) and poly(*S*-ethylsulfonyl-l-homocysteine) (pHcy(SO_2_Et)), along with 3 or 6 pSar arms. The two synthesized miktoarm star polymers with well-defined molecular weight and chain architecture, pLys_20_pHcy(SO_2_Et)_20_(pSar_100_)_3_ (AB_3_) and pLys_20_pHcy(SO_2_Et)_20_(pSar_50_)_6_ (AB_6_), were then investigated in the formulation and delivery efficacy of large nucleic acids (mRNA and pDNA) on Jurkat T cells and D1 cells. Importantly, we used aqueous hydrolysis of *S*-ethylsulfonyl groups for disulfide formation instead of employing oligo thiol cross-linkers.^18,39−41^ The cross-linking reaction yields disulfide bonds stabilizing the PICMs while being cleaved upon endocytosis in antigen-presenting cells or cells with high metabolic activity, e.g., cancer cells.^41^ Previous work suggests that the transfection efficiency of polyplexes is not only cell-dependent but also outlines that mRNA has a stronger binding ability to pLys than pDNA, and thus different transfection efficacies were observed.^42^ This study outlines a synthetic route to reactive miktoarm star polymers combining the shielding ability of 3 or 6 pSar arms with disulfide stabilization (pHcy(SO_2_Et))^43,44^ and efficient nucleic acid binding (pLys).^45,46^ Moreover, it provides insights into the relationship between polymer microstructure, namely, the number shielding polymers in block ionomers, and the biological activity of polymeric carrier systems in nucleic acid delivery.

## 2 Experimental Section

### 2.1 Materials

Materials, solvents, and chemicals were obtained from commercial sources such as Sigma-Aldrich (Munich, Germany), Acros Organics (Nidderau, Germany), Roth (Karlsruhe, Germany), Fisher Scientific (Schwerte, Germany), or Fluka (Munich, Germany) at the highest purity available and were used without further purification unless stated otherwise. Sarcosine, l-methionine, and *N*-ε-*Boc*-protected lysine was purchased from ORPEGEN and dried under a vacuum before NCA synthesis. Tetrahydrofuran (THF) and *n*-hexane were dried over Na/K prior to use. *N*,*N*-Dimethylformamide (DMF) was bought from Acros (99.8%, extra dry over molecular sieve) and purified by repetitive freeze–thaw cycles to remove dimethylamine prior to use. Diphosgene was purchased from Alfa Aesar and used without further purification. Deuterated solvents were obtained from Deutero GmbH and were used as received. *N*,*N*-Diisopropylethylamine (DIPEA) and triethylamine (TEA) were purchased from ABCR, dried over CaH_2_ and molecular sieves (4 Å), and fractionally distilled under a N_2_ atmosphere. Milli-Q water was prepared by using a Milli-Q Reference A^+^ System. Water at a resistivity of 18.2 MΩ/cm and a total organic carbon content of <5 ppm was used throughout this study. Dulbecco’s Modified Eagle’s Medium/F12 (DMEM/F12), RPMI-1640, EDTA, trypan blue, UltraPure agarose, Subcloning Efficiency DH5a competent cells, HiPure Plasmid Midiprep kit, 2-mercaptoethanol, and GlutaMAX-I were purchased from Thermo Fisher Scientific (Landsmeer, The Netherlands). DPBS[−], l-glutamine, and penicillin–streptomycin (10,000 U/mL penicillin, 10,000 U/mL streptomycin) were purchased from Lonza Bioscience. GelRed was bought from Bioconnect. Fetal bovine serum was obtained from SERANA (Brandenburg, Germany). R1 supernatant, and dendritic D1 cell line were kindly provided by Leiden University Medical Center. Jurkat T cell line was provided by Leiden University. Chemically modified messenger mRNA encoding Green Fluorescence Protein (GFP) was purchased from Cellerna Bioscience (Baesweiler, Germany).

### 2.2 Methods

#### 2.2.1 Nuclear Magnetic Resonance Spectroscopy

^1^H nuclear magnetic resonance (NMR) and Diffusion Ordered Spectroscopy (DOSY) spectra were recorded on a Bruker Avance I (AV-400 MHz) at room temperature and a concentration of 15 mg/mL. The chemical shifts (δ) are given in parts per million (ppm) relative to tetramethylsilane (TMS). NMR spectra were processed with the software MestReNova (version 12.0.2) from Mestrelab Research. Samples were prepared in deuterated solvents, and their corresponding signals were referenced to residual nondeuterated solvent signals.

#### 2.2.2 Attenuated Total Reflectance Fourier Transform Infrared Spectroscopy

Measurements were performed at ambient temperature on a FT/IR-4100 (JASCO) equipped with an ATR sampling accessory (MIRacle TM, Pike Technologies), and spectra were visualized as well as analyzed using Spectra Manager 2.0 software (JASCO). To monitor NCA polymerization progress, attenuated total reflectance Fourier transform infrared spectroscopy (ATR-FTIR) was utilized, correlating progress to respective NCA carbonyl bands at 1853 and 1786 cm^–1^.

#### 2.2.3 Size-Exclusion Chromatography

Size-exclusion chromatography (SEC) was performed at 40 °C using HFIP as the eluent, which was equipped with 3 g/L potassium trifluoroacetate. The column material was modified silica gel (PFG columns, particle size: 7 μm, porosity: 100 and 4000 Å), purchased from PSS Polymer Standards Service GmbH. For polymer detection, a UV detector (JASCO UV-2075+) at a wavelength of λ = 230 nm. Molecular weights were determined by using a polysarcosine^12^ calibration with toluene as the internal standard. The elution diagram was evaluated with a PSS WinGPC (PSS Polymer Standard Service GmbH). Samples were prepared at 1 mg/mL and filtered through GHP syringe filters (0.2 μm pore size, Acrodisc) prior to injection.

#### 2.2.4 Dynamic Light Scattering

Single-angle dynamic light scattering (DLS) experiments were performed with a ZetaSizer Nano ZS instrument (Malvern Instruments Ltd., Worcestershire, UK) equipped with a He–Ne laser (λ = 632.8 nm) as the incident beam. All DLS measurements were performed at 25 °C and a detection angle of 173°. Samples were prepared at a concentration of 0.1 mg/mL. For nucleic acid-loaded PICMs, both the hydrodynamic diameter and ζ-potential were checked in HEPES buffer (10 mM, pH 7.4) at a polymer final concentration of 1 μg/mL.

#### 2.2.5 Plasmid Transformation, Isolation, and Purification

##### 2.2.5.1 Plasmid Transformation

Agar plates with antibiotics were freshly prepared with a final ampicillin concentration of 50 μg/mL and kept at 37 °C prior to use. Competent*Escherichia coli* cells stored at −80 °C were thawed on ice for approximately 20 min. Afterward, 1 μL containing 100 ng of eGFP-encoded pDNA was transferred into 20 μL of competent cells and gently mixed. The competent cell/pDNA mixture was incubated on ice for another 20 min. The transformation tube was then subjected at heat shock by placing half of the tube into a water bath of 42 °C for 45 s. After the heat shock treatment, the tube was put on ice for 2 min, followed by the addition of 250 μL of lysogeny broth (LB). The bacteria were then left in an 37 °C incubator for 45 min. At the end of incubation, 50 μL of the transformation was transferred and spread on the prepared LB agar plates (ϕ10 cm) containing ampicillin before being incubated at 37 °C overnight. Afterward, a starter culture was prepared. For this purpose, LB medium with ampicillin (50 μg/mL) was prepared. 1 μL of LB medium (+ampicillin) was then transferred into a tube. Using a pipet tip, a colony was selected from the LB agar plate, and the tip was dropped into the tube. After swirling, the tube was covered with a nonairtight cap followed by incubation in a 37 °C shaking incubator overnight. After incubation, the growth of the bacteria was checked by naked eyes, and 50 μL was transferred into an Erlenmeyer flask containing 50 mL of LB medium (+ampicillin). This mixture was incubated in a 37 °C shaking incubator for approximately 16 h. Afterward, the bacterial growth was checked before plasmid isolation.

##### 2.2.5.2 Plasmid Isolation and Purification

Meanwhile, a cell lysate was prepared of 25 mL of overnight LB culture by the following steps. The overnight LB culture was centrifuged at 4000*g* for 10 min, and the remaining medium was removed to harvest the cells. Next, 4 mL of resuspension buffer with RNase was added and resuspended homogeneously by pipetting up and down. Then, 4 mL of lysis buffer was added, and the mixture was gently inverted until it was homogeneous, followed by 5 min of incubation at room temperature. After incubation, 4 mL of precipitation buffer was immediately added and mixed by inverting the tube until it was homogeneous. This mixture was centrifuged at 12,000*g* for 10 min at room temperature to obtain the cell lysate. The cell lysate supernatant was loaded onto the equilibrated column. After the solution was drained, the column was washed twice with 10 mL of wash buffer. The pDNA was then eluted by adding 5 mL of elution buffer to the column and collected in a sterile centrifuge tube. In order to precipitate the pDNA, 3.5 mL of isopropanol was added to the eluent, followed by centrifuging at 12,000*g* for 30 min at 4 °C. The pellet was resuspended in 3 mL of cold 70% ethanol and centrifuged at 12,000*g* for 5 min at 4 °C. The pellet was then put at −20 °C for no longer than 5 min before air-drying for 10 min. The pDNA pellet was resuspended in 200 μL of TE buffer. The pDNA concentration was then quantified using a DeNovix DS-11 Series Spectrophotometer/Fluorometer.

## 3 Synthetic Procedures

### 3.1 Monomers

#### 3.1.1 Sarcosine-*N*-carboxyanhydride (Sar-NCA)

The synthesis was performed according to our previous reports.^48^ The synthesis was confirmed by ^1^H NMR and melting point measurements (mp = 103 °C). ^1^H NMR (400 MHz, CDCl_3_): δ (ppm) = 4.13 (s, 2H, **a**), 3.03 (s, 3H, **b**).

#### 3.1.2 *S*-Ethylsulfonyl-l-homocysteine-*N*-carboxyanhydride (Hcy(SO_2_Et)-NCA)

The synthesis was performed according to our previous reports.^40^ The synthesis was confirmed by ^1^H NMR and melting point measurements (mp = 95 °C). ^1^H NMR (400 MHz, DMSO-*d*_6_): δ (ppm) = 9.14 (s, 1H, **a**), 4.53 (ddd, 1H, **b**), 3.57 (q, 2H, **c**), 3.28–3.16 (m, 2H, **d**), 2.24–2.06 (m, 2H, **e**), 1.30 (t, 3H, **f**).

#### 3.1.3 *N*-ε-*tert*-Butyloxycarbonyl-l-lysine-*N*-carboxyanhydride (Lys(*Boc*)-NCA)

The synthesis was performed according to our previous reports.^27^ The synthesis was confirmed by ^1^H NMR and melting point measurements (mp = 138 °C). ^1^H NMR (400 MHz, DMSO-*d*_6_): δ (ppm) = 9.07 (s, 1H, **a**), 4.53 (t, 1H, **b**), 4.42 (t, 1H, **c**), 2.90 (q, 2H, **d**), 1.79–1.55 (m, 2H, **e**), 1.48–1.21 (m, 13H, **f**).

### 3.2 Tetrafunctional Initiator

#### 3.2.1 Tris{[2-(*tert*-butoxycarbonyl)ethoxy]methyl}methylamine

The synthesis was performed according to our previous reports.^48^ The synthesis was confirmed by ^1^H NMR. ^1^H NMR (400 MHz, CDCl_3_): δ (ppm) = 3.64 (t, 6H, **a**), 3.31 (s, 6H, **b**), 2.45 (t, 6H, **c**), 1.44 (s, 27H, **d**).

#### 3.2.2 *Fmoc*-Ahx-Tris{[2-(*tert*-butoxycarbonyl)ethoxy]methyl}methylamide

The synthesis was performed according to our previous reports.^49^ The synthesis was confirmed by ^1^H NMR. ^1^H NMR (400 MHz, DMSO-*d*_6_): δ (ppm) = 7.88 (d, 2H, **a**), 7.67 (d, 2H, **b**), 7.41 (t, 2H, **c**), 7.32 (t, 2H, **d**), 7.25 (t, 1H, **e**), 6.90 (s, 1H, **f**), 4.27 (d, 2H, **g**), 4.20 (t, 1H, **h**), 3.64–3.47 (m, 12H, **i**), 3.04–2.93 (m, 2H, **j**), 2.38 (t, 6H, **k**), 2.04 (t, 2H, **l**), 1.56–1.29 (m, 33H, **m**).

#### 3.2.3 *Fmoc*-Ahx-Tris[2-(carboxyethoxy)methyl]methylamide

The synthesis was performed according to our previous reports.^49^ The synthesis was confirmed by ^1^H NMR. ^1^H NMR (400 MHz, DMSO-*d*_6_): δ 13.45–11.70 (m, 3H, **a**) 7.86 (d, 2H, **b**), 7.67 (d, 2H, **c**), 7.40 (t, 2H, **d**), 7.32 (t, 2H, **e**), 7.23 (t, 1H, **f**), 6.92 (s, 1H, **g**), 4.28 (d, 2H, **h**), 4.20 (t, 1H, **i**), 3.66–3.44 (m, 12H, **j**), 3.04–2.93 (m, 2H, **k**), 2.42 (t, 6H, **l**), 2.04 (t, 2H, **m**), 1.52–1.35 (m, 6H, **n**).

#### 3.2.4 *Fmoc*-Ahx-Tris{[2-(*N*-Cbz-ethylendiaminecarbonyl)ethoxy]methyl}methylamide

The synthesis was performed according to our previous reports.^49^ The product was confirmed by ^1^H NMR. ^1^H NMR (400 MHz, DMSO-*d*_6_): δ (ppm) = 7.97–7.76 (m, 5H, **a**), 7.67 (d, 2H, **b**), 7.40 (t, 2H, **c**), 7.38–7.28 (m, 17H, **d**), 7.25 (t, 4H, **e**), 6.99 (s, 1H, **f**), 5.00 (s, 6H, **g**), 4.27 (d, 2H, **h**), 4.20 (t, 1H, **i**), 3.63–3.43 (m, 12H, **j**), 3.17–3.07 (m, 6H, **k**), 3.06–2.98 (m, 6H, **l**), 2.95 (q, 2H, **m**), 2.27 (t, 6H, **n**), 2.05 (t, 2H, **o**), 1.49–1.29 (m, 6H, **p**).

#### 3.2.5 Ahx-Tris{[2-(*N*-Cbz-ethylendiaminecarbonyl)ethoxy]methyl}methylamide

The synthesis was performed according to our previous reports.^49^ The product was confirmed by ^1^H NMR. ^1^H NMR (400 MHz, DMSO-*d*_6_): δ (ppm) = 7.97–7.80 (m, 3H, **a**), 7.48–7.23 (m, 18H, **b**), 6.98 (s, 1H, **c**), 5.00 (s, 6H, **d**), 3.64–3.43 (m, 12H, **e**), 3.17–3.07 (m, 6H, **f**), 3.06–2.99 (m, 6H, **g**), 2.85–2.55 (m, 2H, **h**), 2.27 (t, 6H, **i**), 2.05 (t, 2H, **j**), 1.49–1.29 (m, 6H, **k**).

#### 3.2.6 *Boc*-Ahx-Tris{[2-(*N*-Cbz-ethylendiaminecarbonyl)ethoxy]methyl}methylamide

The synthesis was performed according to our previous reports.^49^ The product was confirmed by ^1^H NMR. ^1^H NMR (400 MHz, DMSO-*d*_6_): δ (ppm) = 7.89 (t, 3H, **a**), 7.40–7.28 (m, 15H, **b**), 7.25 (t, 3H, **c**), 6.99 (s, 1H, **d**), 6.73 (t, 1H, **e**), 5.00 (s, 6H, **f**), 3.57–3.50 (m, 12H, **g**), 3.15–3.08 (m, 6H, **h**), 3.07–2.95 (m, 6H, **i**), 2.87 (q, 2H, **j**), 2.27 (t, 6H, **k**), 2.04 (t, 2H, **l**), 1.50–1.30 (m, 15H, **m**).

### 3.3 Heptafunctional Initiator

#### 3.3.1 Cbz-Ahx-Tris{[2-(*tert*-butoxycarbonyl)ethoxy]methyl}methylamide

The synthesis was performed according to our previous reports.^48^ The product was confirmed by ^1^H NMR. ^1^H NMR (400 MHz, DMSO-*d*_6_): δ (ppm) = 7.39–7.27 (m, 5H, **a**), 7.21 (t, 1H, **b**), 6.90 (s, 1H, **c**), 4.99 (s, 2H, **d**), 3.55–3.52 (m, 12H, **e**), 2.99–2.92 (m, 2H, **f**), 2.38 (t, 6H, **g**), 2.02 (t, 2H, **h**), 1.45–1.38 (m, 27H, **i**), 1.37–1.35 (m, 2H, **j**), 1.31–1.12 (m, 2H, **k**).

#### 3.3.2 Cbz-Ahx-Tris[2-(carboxyethoxy)methyl]methylamide

The synthesis was performed according to our previous reports.^48^ The product was confirmed by ^1^H NMR. ^1^H NMR (400 MHz, DMSO-*d*_6_): δ (ppm) = 7.39–7.27 (m, 5H, **a**), 7.20 (t, 1H, **b**), 6.92 (s, 1H, **c**), 4.99 (s, 2H, **d**), 3.56–3.51 (m, 12H, **e**), 2.99–2.92 (m, 2H, **f**), 2.41 (t, 6H, **g**), 2.03 (t, 2H, **h**), 1.45–1.43 (m, 4H, **i**), 1.37–1.35 (m, 2H, **j**), 1.31–1.12 (m, 2H, **k**).

#### 3.3.3 Cbz-Ahx-Tris{[2-(*N*-*Boc*-ethylendiaminecarbonyl)ethoxy]methyl}methylamide

The synthesis was performed according to our previous reports.^48^ The product was confirmed by ^1^H NMR. ^1^H NMR (400 MHz, DMSO-*d*_6_): δ (ppm) = 7.85 (t, 3H, **a**), 7.40–7.28 (m, 5H, **b**), 7.21 (t, 1H, **c**), 6.99 (s, 1H, **d**), 6.78 (t, 3H, **e**), 4.99 (s, 2H, **f**), 3.57–3.50 (m, 12H, **g**), 3.10–3.01 (m, 6H, **h**), 3.00–2.91 (m, 8H, **i**), 2.27 (t, 6H, **j**), 2.05 (t, 2H, **k**), 1.50–1.30 (m, 33H, **l**).

#### 3.3.4 *Boc*-Deprotection of the Tetrafunctional Initiator

First, Cbz-Ahx-Tris{[2-(*N*-*Boc*-ethylendiaminecarbonyl)ethoxy]methyl}methylamide (1.89 g, 1.87 mmol, 1 equiv) was weighed into a round-bottom flask and was then dissolved in 10 mL of dichloromethane. The solution was cooled to 10 °C, and trifluoroacetic acid (10 mL) was added dropwise under stirring with continued cooling. Afterward, the mixture was stirred for an additional hour at room temperature and then concentrated in vacuo. The resulting product was dissolved in 5 mL of H_2_O, filtered, and lyophilized. The pure product was obtained as a brown solid (1.97 g, 1.87 mmol, quant.). The product was confirmed by ^1^H NMR. ^1^H NMR (400 MHz, DMSO-*d*_6_): δ (ppm) = 8.06 (t, 3H, **a**), 7.81 (s, 9H, **b**), 7.40–7.28 (m, 5H, **c**), 7.24 (t, 1H, **d**), 7.01 (s, 1H, **e**), 5.00 (s, 2H, **f**), 3.57–3.50 (m, 12H, **g**), 3.27 (t, 6H, **h**), 3.03–2.92 (m, 2H, **i**), 2.88–2.77 (m, 6H, **j**), 2.32 (t, 6H, **k**), 1.48–1.18 (m, 6H, **m**).

#### 3.3.5 Heptafunctional Initiator

The *Boc*-deprotected tetrafunctional initiator (1.97 g, 1.87 mmol, 1 equiv) was added to a round-bottom flask along with *N*_α_,*N*_ε_-di-*Boc*-l-lysine hydroxysuccinimide ester (4.15 g, 9.35 mmol, 5 equiv), DIPEA (1.05 mL, 6.17 mmol, 3 equiv), and a mixture of 20 mL of chloroform and 20 mL of dimethylformamide. The reaction mixture was stirred at room temperature for 24 h. The completion of the reaction was monitored by TLC (*R*_*f*_ = 0.51, DCM/MeOH, 95:5). Afterward, the reaction mixture was poured into 0.5 M KHSO_4_ and extracted 3 times with dichloromethane. The organic layers were combined and subsequently washed with H_2_O and brine, dried over anhydrous Na_2_SO_4_, and concentrated in vacuo. The crude product was purified by column chromatography (DCM/MeOH: 95:5, *R*_*f*_ = 0.51) and gave 1.62 g (0.95 mmol, 51%) of the pure product as a white solid. The successful synthesis was confirmed by ^1^H NMR. ^1^H NMR (400 MHz, DMSO-*d*_6_): δ (ppm) = 7.90–7.75 (m, 6H, **a**), 7.40–7.28 (t, 3H, **b**), 7.21 (t, 1H, **c**), 6.99 (s, 1H, **d**), 6.85–6.70 (m, 6H, **e**), 4.99 (s, 2H, **f**), 3.79 (t, 3H, **g**), 3.57–3.50 (m, 12H, **h**), 3.17–3.05 (m, 12H, **i**), 3.04–2.90 (m, 2H, **j**), 3.03–2.83 (m, 6H, **k**), 2.28 (t, 6H, **l**), 2.04 (t, 2H, **m**), 1.60–1.13 (m, 78H, **n**).

### 3.4 Initiator Deprotection for Macroinitiator Synthesis

#### 3.4.1 Cbz-Deprotection of the Tetrafunctional Initiator

Into a round-bottom flask, *Boc*-Ahx-Tris{[2-(*N*-Cbz-ethylendiaminecarbonyl)ethoxy]methyl}methyl-amide (500.00 mg, 0.46 mmol, 1 equiv) was weighed in and dissolved in 10 mL of methanol. Afterward, 50 mg of Pd/C (10 wt %) was added, and the suspension was stirred under an H_2_-atmosphere for 48 h. The catalyst was removed by filtration through Celite. After removal of the solvent under reduced pressure and lyophilization, the product was obtained as a white solid (317.80 mg, 0.46 mmol, quant.). The deprotection was confirmed by ^1^H NMR. ^1^H NMR (400 MHz, DMSO-*d*_6_): δ (ppm) = 7.87 (m, 3H, **a**), 7.00 (s, 1H, **b**), 6.78 (t, 1H, **c**), 3.57–3.48 (m, 12H, **d**), 3.15–3.00 (m, 6H, **e**), 2.91–2.83 (m, 2H, **f**), 2.58 (t, 6H, **g**), 2.29 (t, 6H, **h**), 2.05 (t, 2H, **i**), 1.55–1.27 (m, 15H, **j**).

#### 3.4.2 *Boc*-Deprotection of the Heptafunctional Initiator

The *Boc*-deprotection was performed analogously to the method described previously for the tetrafunctional initiator. The corresponding pure product was obtained as a brown solid in quantitative yield. The deprotection was confirmed via ^1^H NMR. ^1^H NMR (400 MHz, DMSO-*d*_6_): δ (ppm) = 8.68–8.5 (m, 3H, **a**), 8.18 (s, 9H, **b**), 7.99 (t, 3H, **c**), 7.82 (s, 9H, **d**), 7.45–7.26 (m, 5H, **e**), 7.23 (t, 1H, **f**), 7.10–6.98 (m, 1H, **g**), 5.08–4.97 (m, 2H, **h**), 3.69 (t, 3H, **i**), 3.64–3.47 (m, 12H, **j**), 3.25–3.10 (m, 12H, **k**), 2.96 (t, 2H, **l**), 2.75 (q, 6H, **m**), 2.30 (t, 6H, **n**), 2.05 (t, 2H, **o**), 1.75–1.60 (m, 6H, **p**), 1.53 (q, 6H, **q**), 1.48–1.20 (m, 12H, **r**).

### 3.5 Polypeptoid Macroinitiators

#### 3.5.1 Three-Arm Macroinitiator Synthesis (*Boc*-(pSar_100_-N_3_)_3_)

In a typical experiment, 10.00 mg (14.47 μmol, 1 equiv) of the triple deprotected initiator was weighed into a predried Schlenk flask with a stir bar, and the material was dried for 2 h under high vacuum. After the initiator was dissolved in 2 mL of DMF, 8.12 μL of freshly distilled DIPEA (47.75 μmol, 3.3 equiv) was added, and the mixture was stirred at room temperature for 3 h. Then, Sar-NCA (499.75 mg, 4.34 mmol, 300 equiv) was transferred under nitrogen counterflow into a predried Schlenk flask, dried for 30 min under high vacuum, and dissolved in 3.0 mL of DMF. The initiator solution was added to the NCA-solution via syringe (yielding a total of 5.00 mL of DMF and a monomer concentration of 100 mg/mL). The polymerization was performed at 0 °C in the absence of light under a dry nitrogen atmosphere. The reaction progress was monitored by IR spectroscopy (disappearance of the NCA peaks at 1853 and 1786 cm^–1^). After completion of the reaction was confirmed by IR, pentafluorophenyl-4-azidobutanoat (47.74 mg, 144.70 μmol, 10 equiv) and DIPEA (24.60 μL, 144.70 μmol, 10 equiv) were added, and the solution was stirred at room temperature for 24 h. Afterward, the polymer was precipitated by pouring into cold diethyl ether and separated by centrifugation (4500 rpm at 4 °C for 20 min). After the liquid fraction was discarded, fresh ether was added, and the polymer was resuspended using sonication. The suspension was centrifuged again, and the procedure was repeated. Afterward, the product was dissolved in H_2_O and lyophilized to obtain 385.78 mg (77%) of the desired polymer as a white fluffy powder. ^1^H NMR (400 MHz, DMSO-*d*_6_): δ (ppm) = 7.89 (m, 3H, **a**), 7.00 (s, 1H, **b**), 6.73 (t, 1H, **c**), 4.52–3.75 (m, 600H (2n), **d**), 3.58–3.44 (m, 12H, **e**), 3.15–3.04 (m, 6H, **f**), 3.03–2.55 (m, 920H (3n), **g**), 2.29 (t, 6H, **h**), 2.04 (t, 2H, **i**), 2.10–1.90 (m, 6H, **j**), 1.45–1.30 (m, 15H, **k**).

#### 3.5.2 Six-Arm Macroinitiator Synthesis (Cbz-(pSar_50_-N_3_)_6_)

The six-arm macroinitiator was realized in the same way as the described three-arm macroinitiator, except for the addition of 6 equiv of DIPEA into the initiator-solution prior to the addition to the NCA solution in order to guarantee activation and simultaneous initiation of all amine groups. 372.10 mg of the six-arm macroinitiator (72%) was obtained as a white powder. ^1^H NMR (400 MHz, DMSO-*d*_6_): δ (ppm) = 7.89 (m, 6H, **a**), 7.40–7.31 (m, 5H, **b**), 7.19 (s, 1H, **c**), 6.98 (s, 1H, **d**), 4.99 (s, 2H, **e**), 4.51–3.77 (m, 603H (2n), **f**), 3.57–3.44 (m, 12H, **g**), 3.17–3.02 (m, 12H, **h**), 3.01–2.65 (m, 932H (3n), **i**), 2.28 (t, 6H, **j**), 2.15–2.00 (m, 14H, **k**), 1.67–1.15 (m, 24H, **l**).

#### 3.5.3 *Boc*-Deprotection of the Three-Arm Macroinitiator (NH_2_-(pSar_100_-N_3_)_3_)

The macroinitiator *Boc*-(pSar_100_-N_3_)_3_ (450.21 mg) was transferred to a round-bottom flask and dissolved in 10 mL of Millipore water. The solution was cooled to 0 °C, and trifluoroacetic acid (10 mL) was added dropwise under stirring with continued cooling. Afterward, the mixture was stirred for 2 h at 0 °C. Afterward, the solution was transferred into a dialysis bag (MWCO, 3.5 kDa) and dialyzed against Millipore water, saturated NaHCO_3_-solution, and again Millipore water, each for 1 day. The deprotected macroinitiator was lyophilized from water, and 290.15 mg (65%) of a white solid was obtained. ^1^H NMR (400 MHz, DMSO-*d*_6_): δ (ppm) = 7.95–7.88 (m, 3H, **a**), 7.01 (s, 1H, **b**), 4.55–3.65 (m, 600H (2n), **c**), 3.57–3.45 (m, 12H, **d**), 3.15–3.03 (m, 6H, **e**), 3.05–2.60 (m, 920H (3n), **f**), 2.28 (t, 6H, **g**), 2.04 (t, 2H, **h**), 2.11–1.90 (m, 6H, **i**), 1.55–1.31 (m, 4H, **j**), 1.30–1.20 (m, 2H, **k**).

#### 3.5.4 Cbz-Deprotection of the Six-arm Macroinitiator Cbz-(pSar_50_-N_3_)_6_

The Cbz-deprotection was performed according to Saroha et al. and modified.^50^ The macroinitiator Cbz-(pSar_50_-N_3_)_6_ (372.10 mg) was transferred to a round-bottom flask and dissolved in 15 mL of methanol. 38.01 mg of nickel(II) chloride hexahydrate (159.91 μmol, 10 equiv) was added to the solution. Afterward, the mixture was cooled to 0 °C, and 18.15 mg of sodium borohydride (479.70 μmol, 30 equiv) was added slowly with vigorous stirring. The black mixture was stirred for 1 h at 0 °C, following for 48 h at room temperature. After the reaction mixture was decanted, it was further purified through a syringe filter (PVDF, 0.2 μm) and transferred into a dialysis bag (MWCO, 3.5 kDa). After successful dialysis against Millipore water, saturated NaHCO_3_-solution, and again Millipore water, each for 1 day, the deprotected macroinitiator was lyophilized from water, and 234.23 mg (63%) of a white solid was obtained. ^1^H NMR (400 MHz, DMSO-*d*_6_): δ (ppm) = 7.85 (t, 6H, **a**), 6.99 (s, 1H, **b**), 4.52–3.75 (m, 603H (2n), **c**), 3.56–3.44 (m, 12H, **d**), 3.17–3.02 (m, 12H, **e**), 3.01–2.63 (m, 932H (3n), **f**), 2.41–2.33 (m, 6H, **g**), 2.23–1.96 (m, 14H, **h**), 1.65–1.15 (m, 24H, **i**).

### 3.6 Cross-Linkable and Complexing Miktoarm Star Polymers (PeptoMiktoStars)

#### 3.6.1 AB_3_ PeptoMiktoStar (pLys(*Boc*)_20_pHcy(SO_2_Et)_20_(pSar_100_-N_3_)_3_)

Introduction of the pHcy(SO_2_Et)-*block* (pHcy(SO_2_Et)_20_(pSar_100_-N_3_)_3_)—under a nitrogen counterflow, the deprotected macroinitiator NH_2_-(pSar_100_-N_3_)_3_ (173.43 mg, 7.80 μmol, 1 equiv) was transferred into a predried Schlenk tube equipped with a stir bar. After being suspended in toluene, the macroinitiator was dried overnight using a high vacuum. On the following day, the substance was dissolved in 2.00 mL of dry DMF, and the resulting solution was then cooled to −10 °C. Subsequently, Hcy(SO_2_)-NCA (39.51 mg, 156.01 μmol, 20 equiv) was introduced into another predried Schlenk flask under a nitrogen counterflow. The flask was then subjected to high vacuum drying for 30 min before the NCA was dissolved in 1.00 dry DMF. After the solutions were cooled to −10 °C, the NCA-solution was added to the macroinitiator solution via a syringe. In the absence of light and under a dry nitrogen atmosphere, the polymerization was conducted at −10 °C. After 7 days, the reaction was verified as complete using IR spectroscopy, and the polymer was then precipitated into diethyl ether and centrifuged, and this process was repeated twice. Following this, the product was dissolved in water, purified by repetitive spin filtration (Amicon Ultra, MWCO 10 kDa), and subjected to lyophilization, resulting in 166.09 mg of the desired polymer as a white powder (78%). ^1^H NMR (400 MHz, DMSO-*d*_6_): δ (ppm) = 8.42–8.25 (m, 20H (1n), **a**), 7.96–7.82 (m, 3H, **b**), 7.00 (s, 1H, **c**), 4.65–3.77 (m, 620H (2n + 1n), **d**), 3.64–3.44 (m, 52H (2n), **e**), 3.25–3.05 (m, 46H (2n), **f**), 3.04–2.65 (m, 920H (3n), **g**), 2.28 (t, 6H, **h**), 2.20–1.84 (m, 48H (2n), **i**), 1.50–1.30 (m, 4H, **j**), 1.29–1.15 (m, 62H (3n), **k**).

Introduction of the pLys(*Boc*)-*block* (pLys(*Boc*)_20_pHcy(SO_2_Et)_20_(pSar_100_-N_3_)_3_)—the introduction of the Lys(*Boc*)-*block* followed a procedure analogous to the first block using the Lys(*Boc*)-NCA, except that the polymerization was conducted at 0 °C. Afterward, the product was dissolved in H_2_O and lyophilized to obtain 168.34 mg (80%) of the desired polymer as a white fluffy powder. ^1^H NMR (400 MHz, DMSO-*d*_6_): δ (ppm) = 8.45–8.24 (m, 20H (1n), **a**), 8.03–7.90 (m, 23H (1n), **b**), 7.01 (s, 1H, **c**), 6.75–6.60 (m, 20H (1n), **d**), 4.62–3.72 (m, 640H (2n + 1n + 1n), **e**), 3.64–3.43 (m, 52H (2n), **f**), 3.27–3.06 (m, 46H (2n), **g**), 3.05–2.64 (m, 960H (3n + 2n), **h**), 2.28 (t, 6H, **i**), 2.19–1.87 (m, 48H (2n), **j**),1.70–1.18 (m, 366H (15n + 3n), **k**).

#### 3.6.2 Deprotected AB_3_ PeptoMiktoStar (pLys_20_pHcy(SO_2_Et)_20_(pSar_100_-N_3_)_3_)

In order to remove the *Boc*-protective groups from the pLys(*Boc*)-*block*, the final AB_3_ PeptoMiktoStar (168.34 mg, 5.29 μmol, 1 equiv) was dissolved in 5 mL of Milli-Q water and stirred while being cooled in an ice bath. Dropwise addition of 5 mL of TFA was carried out, and the resulting solution was stirred for 2 h at 0 °C, followed by overnight stirring at ambient temperature. Subsequently, the solution was transferred to a dialysis bag (MWCO 3.5 kDa) and dialyzed against Milli-Q water for 2 days. Following lyophilization, the deprotected polymer was obtained as a colorless powder (141.35 mg, 85%). ^1^H NMR (400 MHz, DMSO-*d*_6_): δ (ppm) = 8.45–8.20 (m, 20H (1n), **a**), 8.01–7.90 (m, 23H (1n), **b**), 7.02 (s, 1H, **c**), 4.51–3.75 (m, 640H (2n + 1n + 1n), **d**), 3.28–3.04 (m, 46H (2n), **e**), 3.03–2.64 (m, 960H (3n + 2n), **f**), 2.28 (t, 6H, **g**), 2.22–1.74 (m, 48H (2n), **h**),1.71–1.38 (m, 180H (9n), **i**), 1.35–1.25 (m, 66H (3n), **j**).

#### 3.6.3 AB_6_ PeptoMiktoStar (pLys(*Boc*)_20_pHcy(SO_2_Et)_20_(pSar_50_-N_3_)_3_)

Introduction of the pHcy(SO_2_Et)-*block* (pHcy(SO_2_Et)_20_(pSar_50_-N_3_)_6_)—the polymerization was conducted following the procedure outlined in Section 3.4.1, which involved the introduction of the pHcy(SO_2_Et)-*block* into the AB_3_ architecture. After lyophilization, 171.26 mg of the desired polymer was obtained as a white powder (67%). ^1^H NMR (400 MHz, DMSO-*d*_6_): δ (ppm) = 8.54–8.27 (m, 20H (1n), **a**), 7.94–7.83 (m, 6H, **b**), 7.00 (s, 1H, **c**), 4.66–3.73 (m, 620H (2n + 1n), **d**), 3.60–3.43 (m, 52H (2n), **e**), 3.26–3.05 (m, 52H (2n), **f**), 3.01–2.60 (m, 932H (3n), **g**), 2.31 (t, 6H, **h**), 2.23–1.82 (m, 54H (2n), **i**), 1.39–1.24 (m, 84H (3n), **j**).

Introduction of the pLys(*Boc*)-*block* (pLys(*Boc*)_20_pHcy(SO_2_Et)_20_(pSar_50_-N_3_)_6_)—the polymerization was conducted following the procedure outlined for the introduction of the pHcy(SO_2_Et)-*block* into the AB_3_ architecture. After lyophilization, 171.26 mg of the desired polymer was obtained as a white powder (67%). ^1^H NMR (400 MHz, DMSO-*d*_6_): δ (ppm) = 8.65–7.81 (m, 46H (1n + 1n), **a**), 7.02 (s, 1H, **b**), 6.85–6.60 (m, 20H (1n), **c**), 4.61–3.73 (m, 640H (2n + 1n + 1n)), 3.61–3.44 (m, 52H (2n), **e**), 3.24–3.04 (m, 52H (2n), **f**), 3.03–2.67 (m, 972H (3n + 2n), **g**), 2.30 (t, 6H, **h**), 2.23–1.86 (m, 54H (2n), **i**), 1.50–1.17 (m, 384H (15n + 3n), **j**).

#### 3.6.4 Deprotected AB_6_ PeptoMiktoStar (pLys_20_pHcy(SO_2_Et)_20_(pSar_50_-N_3_)_6_)

The *Boc*-deprotection of the AB_6_ PeptoMiktoStars was conducted following the same procedure as that employed for the AB_3_ PeptoMiktoStars outlined before. After lyophilization was completed, the deprotected AB_6_ star was obtained as a colorless powder (120.21 mg, 77%). ^1^H NMR (400 MHz, DMSO-*d*_6_): δ (ppm) = 8.45–8.20 (m, 46H (1n + 1n), **a**), 6.99 (s, 1H, **b**), 4.55–3.85 (m, 640H (2n + 1n + 1n), **c**), 3.77–3.24 (m, 52H (2n), **d**), 3.23–3.04 (m, 52H (2n), **e**), 3.03–2.55 (m, 972H (3n + 2n), **f**), 2.36 (t, 6H, **g**), 2.20–1.80 (m, 54H (2n), **h**), 1.79–1.45 (m, 180H (9n), **i**), 1.44–1.25 (m, 84H (3n), **j**).

### 3.7 End Group Modification-Dye Labeling of PeptoMiktoStars

#### 3.7.1 AF647-Labeled pLys_20_(pHcy(SO_2_Et)_20_(pSar_50_)_6_)

The dye AlexaFluor647-DBCO was conjugated via a strain-promoted azide–alkyne coupling reaction (SPAAC). In a typical experiment, 10.05 mg of pLys_20_pHcy(SO_2_Et)_20_(pSar_50_-N_3_)_6_ (0.31 μmol, 1 equiv) and 0.34 mg of AlexaFluor647-DBCO (0.31 μmol, 1 equiv) were weighed into a separated Schlenk flask. After dissolving each in 1 mL abs. DMF, the solutions were mixed, and the labeling reaction was carried out at room temperature for 3 days under light exclusion. Next, the reaction mixture was purified by dialysis against methanol (MWCO 10 kDa) and subsequent repetitive spin filtration (Amicon Ultra, MWCO 10 kDa) in a mixture of water/methanol (1:1). Afterward, the product was lyophilized to obtain 9.35 mg (0.28 μmol, 89%) of the labeled PeptoMiktoStar as a fluffy powder.

### 3.8 Further Reagents

#### 3.8.1 Pentafluorophenyl-4-azidobutanoat

The synthesis of the azide capping agent was performed according to our previous reports.^51^^1^H NMR (400 MHz, CDCl_3_): δ (ppm) = 3.46 (t, 2H, **a**), 2.79 (t, 2H, **b**), 2.05 (m, 2H, **c**).

### 3.9 Preparation of PICMs

The polymer and nucleic acid were diluted with HEPES buffer (10 mM, pH 7.4). The PICMs were prepared depending on the desired N/P ratio by adding nucleic acid into polymer solution at equal volume. After vortexing for 30 s, the mixture was incubated at room temperature for 40 min prior to use.

### 3.10 Complexation of mRNA and pDNA PICMs

The complexation ability of polymers was examined by using 1% agarose gel electrophoresis. According to the defined N/P ratio, 12.5 μL of nucleic acid solution (150 ng pDNA or mRNA) and 12.5 μL of p(Lys)_20_-p(Cys)_20_-p(Sar)_100×3_ (AB_3_) or poly(Lys)_20_-(Cys)_20_-pSar_50×6_ (AB_6_) with different concentrations were mixed. Free or naked mRNA or pDNA (150 ng) was used as a control. After incubation for 40 min at room temperature, 5 μL of glucose (50%, w/v) was added to each sample prior to gel loading. Gel electrophoresis was conducted at 100 mV for 40 min. The gel was imaged using a ChemiDoc MP Gel Imaging system.

### 3.11 Stability of PICMs

PICMs containing pDNA/ABn or mRNA/ABn at N/P 10 were prepared as described above. In brief, ABn polymers were mixed with pDNA or mRNA in equal volume. After incubation at room temperature for 40 min, the formulated PICMs were exposed to glutathione at different final concentrations (10 μM or 10 mM) at 37 °C for 1.5 h. Afterward, heparin sulfate (HS) was added to the samples to a final concentration of 0.5 or 2.0 mg/mL. After 1 h of incubation at room temperature, 5 μL of glucose (50%, w/v) was added to each sample prior to gel loading. Gel electrophoresis was conducted at 100 mV for 40 min. The gel was imaged using the ChemiDoc MP Gel Imaging system.

### 3.12 Cell Culture

D1 dendritic cells were cultured in 70% (v/v) DCCM [IMDM without l-glutamine, 8% (v/v) FCS, 80 IU/mL penicillin, 2 mM Glutamax, and 50 μM 2-ME] with 30% (v/v) R1 supernatant. The Jurkat T cell line was cultured in RPMI-1640 supplemented with 10% FBS, 100 μg/mL penicillin/streptomycin, and 2 mM l-glutamine. All cells were incubated at 37 °C in a humidified atmosphere containing 5% CO_2_.

### 3.13 Cellular Uptake and Transfection

D1 cells were seeded in 96-well F-bottom plates at a density of 50,000 cells/well, and Jurkat T cells were seeded in 96-well U-bottom plates at a density of 40,000 cells/well. All cells were incubated at 37 °C in a humidified atmosphere containing 5% CO_2_ for 24 h prior to the treatment. Briefly, nucleic acid-loaded PICMs at varying N/P ratios were applied to cells, while nontreated cells and cells exposed to HEPES buffer served as negative controls. In each well, 20 μL of each sample were transferred into each well, followed by an addition of 80 μL of fresh culture medium, and it was then incubated for 48 and 24 h for pDNA and mRNA, respectively. For the D1 cell, the cell supernatant in each well was transferred to 96-well plate U-bottom at the end of incubation. 50 μL of EDTA (2 mM) was added to each well and incubated at room temperature for 5 min. Afterward, cells were harvested and transferred into the same 96-well U-bottom plate, followed by centrifugation at 500*g* for 5 min at room temperature. The supernatant was removed, and 200 μL of flow buffer (1% bovine serum albumin, 0.1% NaN_3_, DPBS[−]) was added to each well to resuspend the cells. For Jurkat T cells, after incubation, the plate was centrifuged directly at 500*g* for 5 min at room temperature. The cell pellet from each well was resuspended in 200 μL of flow buffer. The fluorescence signal was determined by flow cytometry on a CytoFLEXS Flow Cytometer device (Beckman Coulter, Woerden, The Netherlands) and analyzed by FlowJo software version 10.8.1.

### 3.14 Cell Viability

D1 cells were seeded at a density of 50,000 cells/well in 96-well F-bottom plates (Greiner Bio-One, Alphen aan den Rijn, The Netherlands), and Jurkat T cells were seeded at a density of 40,000 cells/well in 96-well U-bottom plates (Greiner Bio-One, Alphen aan den Rijn, The Netherlands) for 24 h prior to use. MTT stock solution (11 mg/mL) was prepared beforehand by dissolving MTT (3-(4,5-dimethylthiazol-2-yl)-2,5-diphenyltetrazolium) in DPBS[−] in the absence of light. 20 μL of polymer solutions were added to each well to a final volume of 180 μL and incubated for 24 h at 37 °C. After the incubation, 20 μL of MTT stock solution was added to each well for another 3 h incubation at 37 °C in a light-free condition. At the end of incubation, MTT solutions in each well were aspirated, and 200 μL of pure DMSO was added to each well, followed by gentle shaking on a plate shaker at 150 rpm for 20 min. MTT absorbance was measured on a Spark Microplate Reader (Tecan Austria GmbH) at 590 and 690 nm, respectively. The cell viability was then determined according to the previous report.^47^

### 3.15 Statistical Analysis

The results are presented as mean ± standard deviation. Experiments were carried out in at least three replicates on independent days. The significance was determined using 2-way ANOVA with GraphPad Prism 10. The following asterisks indicate statistical significance: *p < 0.05; **p < 0.01; ***p < 0.001, ****p < 0.0001.

## 4 Results and Discussion

**Figure 1:**
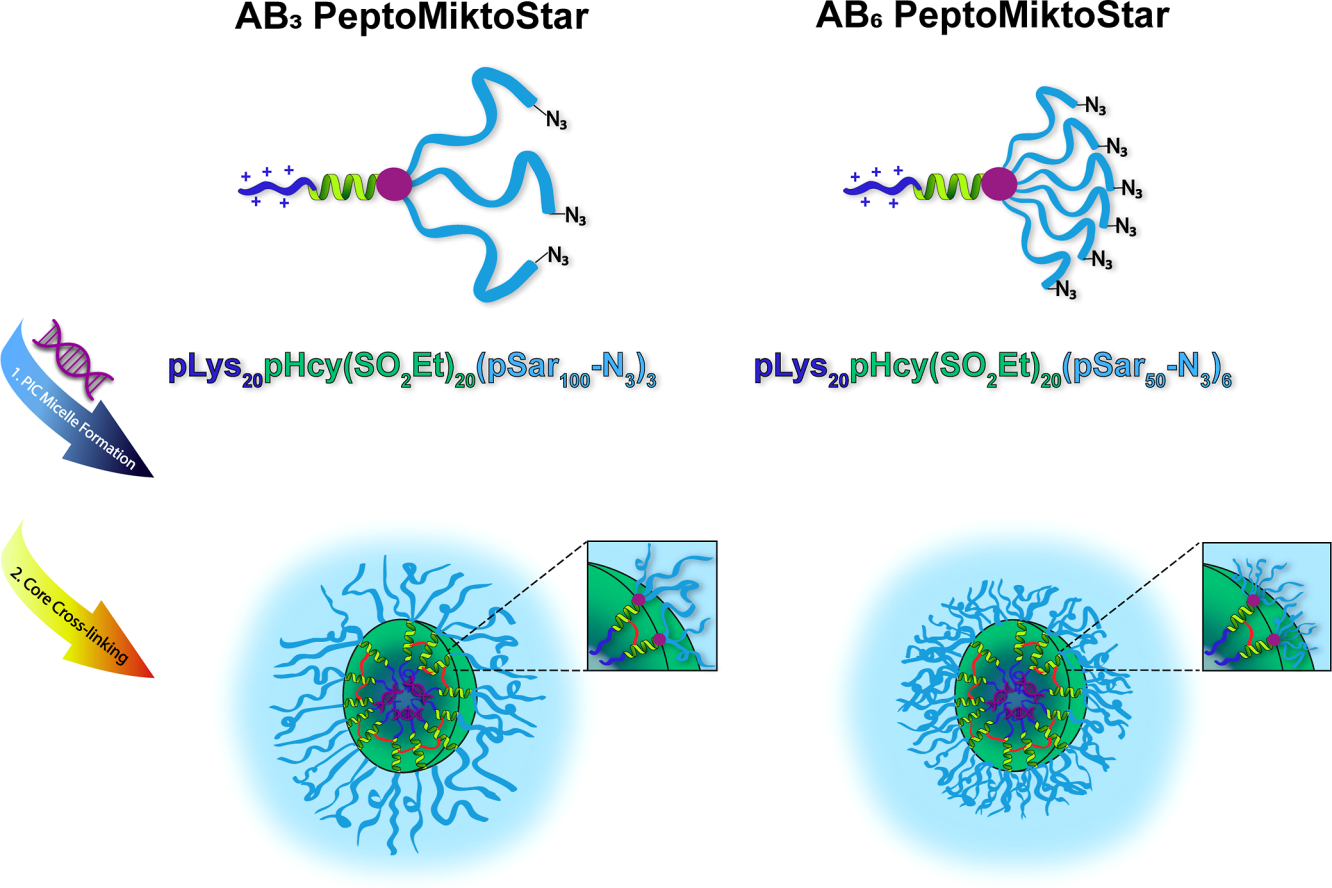
Overview of the realized cationic and cross-linkable polypept(o)ide-based miktoarm star polymers (PeptoMiktoStars) and the corresponding PICM formation.

In order to establish a synthetic route for the PeptoMiktoStars depicted in Figure 1, we aim to employ the recently introduced core-first approach. The combination with an orthogonal protecting group strategy enabled the incorporation of sensitive functional groups, such as the *S*-alkylsulfonyl protecting group, in the final step of AB_3_ miktoarm star polymer synthesis.^49^ To facilitate the creation of AB_6_ PeptoMiktoStars, the previously developed tetrafunctional initiator, Cbz-Ahx-Tris{[2-(*N*-*Boc*-ethylendiaminecarbonyl)ethoxy]methyl}methylamide was initially deprotected from the *tert*-butyloxy-carbonyl (*Boc*) protecting groups through a straightforward deprotection process under acidic conditions (Figure S12). Thereafter, it was successfully engaged in a peptide coupling reaction with *N*_α_-*N*_ε_-di-*Boc*-l-lysine-hydroxysuccine-imidester to yield a heptafunctional initiator with six *Boc*-protected amine functionalities.

**Scheme 1:**
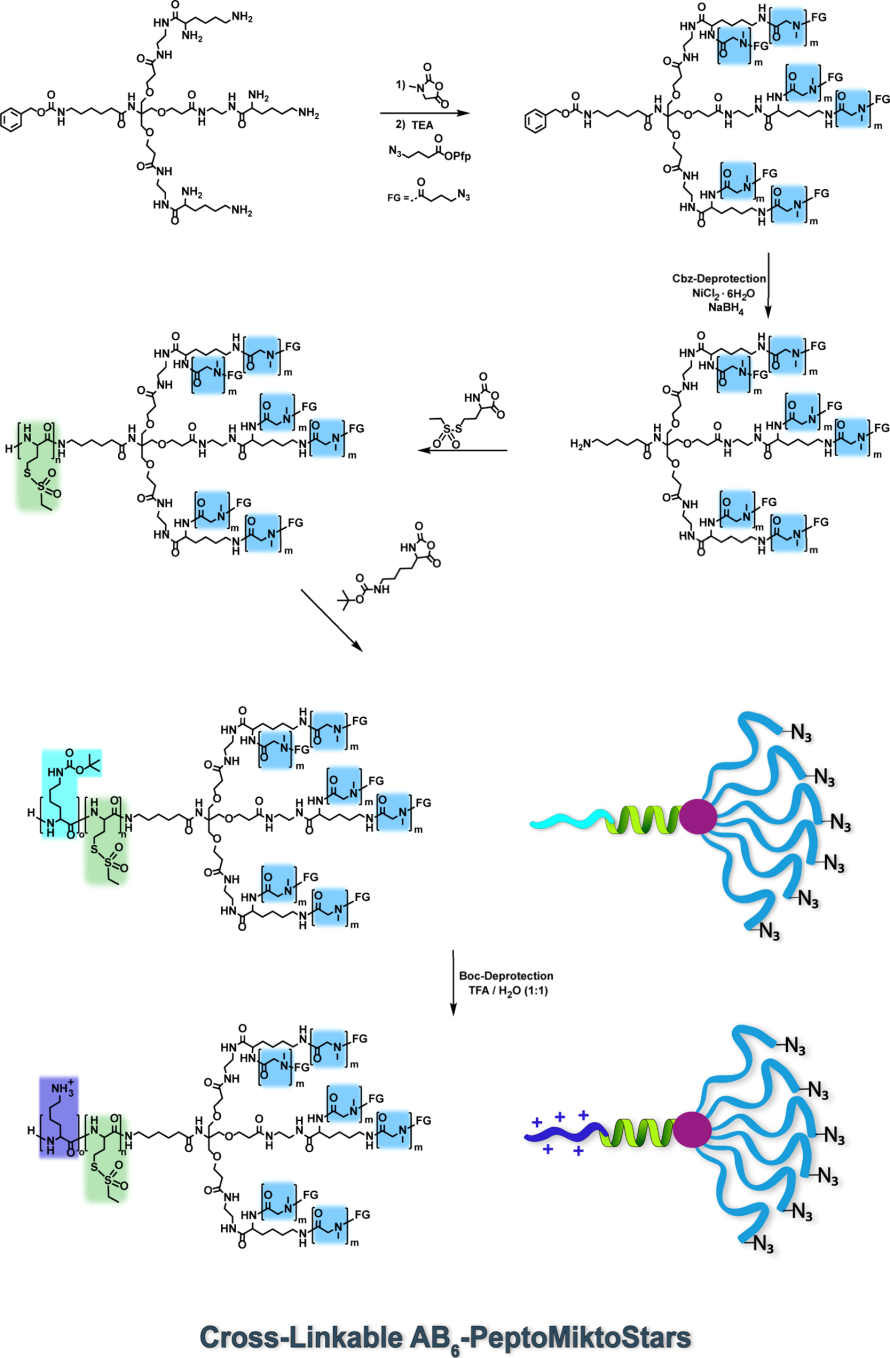
Synthesis of Cross-Linkable AB_6_ PeptoMiktoStars

The synthesis of both initiators involved a 6-step process originating from tris(hydroxymethyl)-aminomethane (TRIS). A high degree of purity of the final initiator is crucial for obtaining well-defined structures through the subsequent ROP of NCAs. The presence of impurities would impede the polymerization, as impurities can terminate growing species or initiate themselves, resulting in the generation of linear polymer contaminations and consequently impair the uniformity of miktoarm star polymers. The purity of components was analyzed by ^1^H NMR, as depicted in Figures S6 and S17, melting point (NCA), and Karl Fischer titration.^52,53^ Since the strategy for the synthesis of AB_3_ and AB_6_ miktoarm stars, as detailed in Scheme 1 and Figure S1, involves multiarm polypeptoid macroinitiators, triple- and hexafunctional core structures are necessary for the synthesis of the corresponding macroinitiators. The successful deprotection, as indicated by ^1^H NMR (Figures S19 and S20), yielded the desired core structures, enabling the synthesis of the final macroinitiators.

By conducting the controlled living ROP of Sar-NCA in absolute dimethylformamide (DMF) at 0 °C, the simultaneous introduction of three and respective six pSar-arms was achieved.^48^ Based on former work of our group on polyion complex micelles (PICMs) and polyplexes, a degree of polymerization (DP_*n*_) of 300 (3 × 100 and 6 × 50) was selected, taking into account the desired stability and size of the resulting nanostructures.^41,45,46^ IR spectroscopy was used to track the conversion of the monomer with the NCA vibration bands at 1853 and 1786 cm^–1^ disappearing when the NCA is consumed. After confirming complete monomer conversion, a quenching step was conducted, using an azide-containing capping agent. The introduction of azide functionalities on each pSar-arm offers the potential to accomplish multiple modifications at the hydrophilic pSar end groups of the resulting miktoarm star structure, thereby allowing chemoselective [3 + 2] cycloaddition reactions with alkyne-modified dyes or targeting molecules under mild reaction conditions.^51,54^

**Figure 2:**
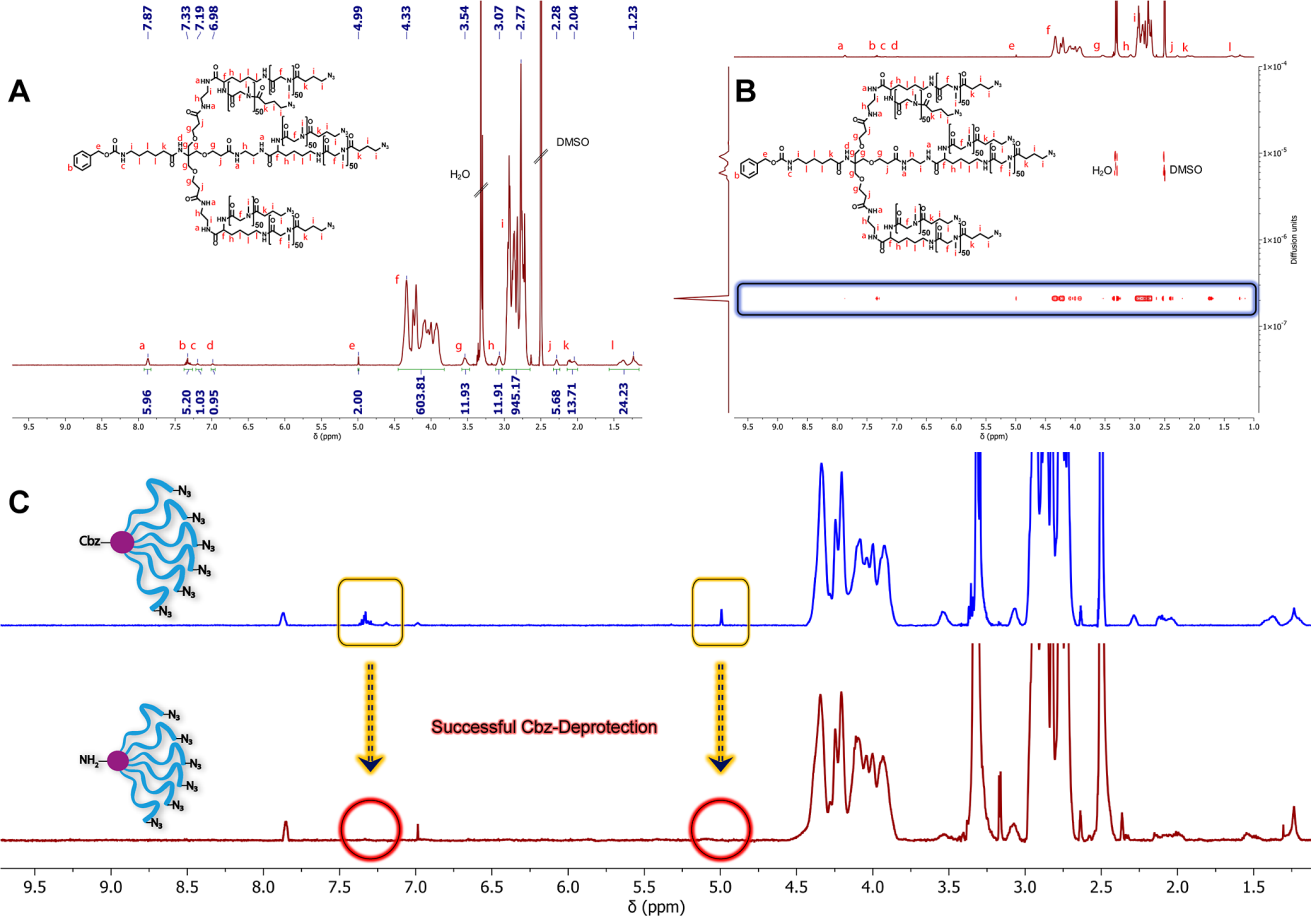
Characterization of the protected and deprotected six-arm macroinitiator. (A) ^1^H NMR of the protected 6-arm macroinitiator Cbz-(pSar_50_-N_3_)_6_. (B) ^1^H DOSY NMR of the protected 6-arm macroinitiator Cbz-(pSar_50_-N_3_)_6_. (C) ^1^H NMR spectra after successful Cbz-deprotection.

To ascertain the successful syntheses, the three- and six-arm macroinitiators were subsequently precipitated in cold diethyl ether and characterized using ^1^H NMR, ^1^H DOSY, and SEC in hexafluoroisopropanol (HFIP) (Figures 2, S22 and S23). The analytical data are summarized in Table 1.

**Table 1:** Analytical Data of the AB_3_ and AB_6_ Cross-Linkable PeptoMiktoStars and Their Corresponding Macroinitiators

polymer	DP_*n*_^a^ (calc.)	DP_*n*_^b^ (determ.)	*M*_n_^c^/Da	*M*_w_^d^/Da	*D̵*^d^
*Boc*-(pSar_100_-N_3_)_3_	300	301	22,300	23,700	1.1
Cbz-(pSar_50_-N_3_)_6_	300	302	23,100	20,500	1.1
pLys(*Boc*)_20_pHcy(SO_2_Et)_20_(pSar_100_-N_3_)_3_	20/20/300	20/21/301	31,300	33,500	1.2
pLys(*Boc*)_20_pHcy(SO_2_Et)_20_(pSar_50_-N_3_)_6_	20/20/300	20/20/302	31,700	30,000	1.2

a Calculated degree of polymerization using DP_*n*_ = [M]/[I].

b Determined by ^1^H NMR in DMSO-*d*_6_.

c Determined by obtained chain lengths from ^1^H NMR after subtraction of the corresponding initiator’s molecular weight.

d Determined by SEC in HFIP using linear pSar standards.

DP_*n*_ can be easily determined by relating the characteristic protons of the pSar backbone with the ones of the tetrafunctional or heptafunctional initiator in ^1^H NMR, which leads to a less than 1% deviation between calculated and obtained degree of polymerization for pSar and 5% for the polypeptide segments. In the case of the three-arm macroinitiator, the backbone protons of pSar can be related to the signals of the *Boc*-protecting group and the aminohexyl-spacer (Ahx), while for the six-arm macroinitiator, the protons of the benzylic methylene group of the respective Cbz-protecting group can be utilized. The obtained data highlight the concise control over the pSar and polypeptide chain length accomplished by adjusting the monomer-to-initiator ratio.

**Figure 3:**
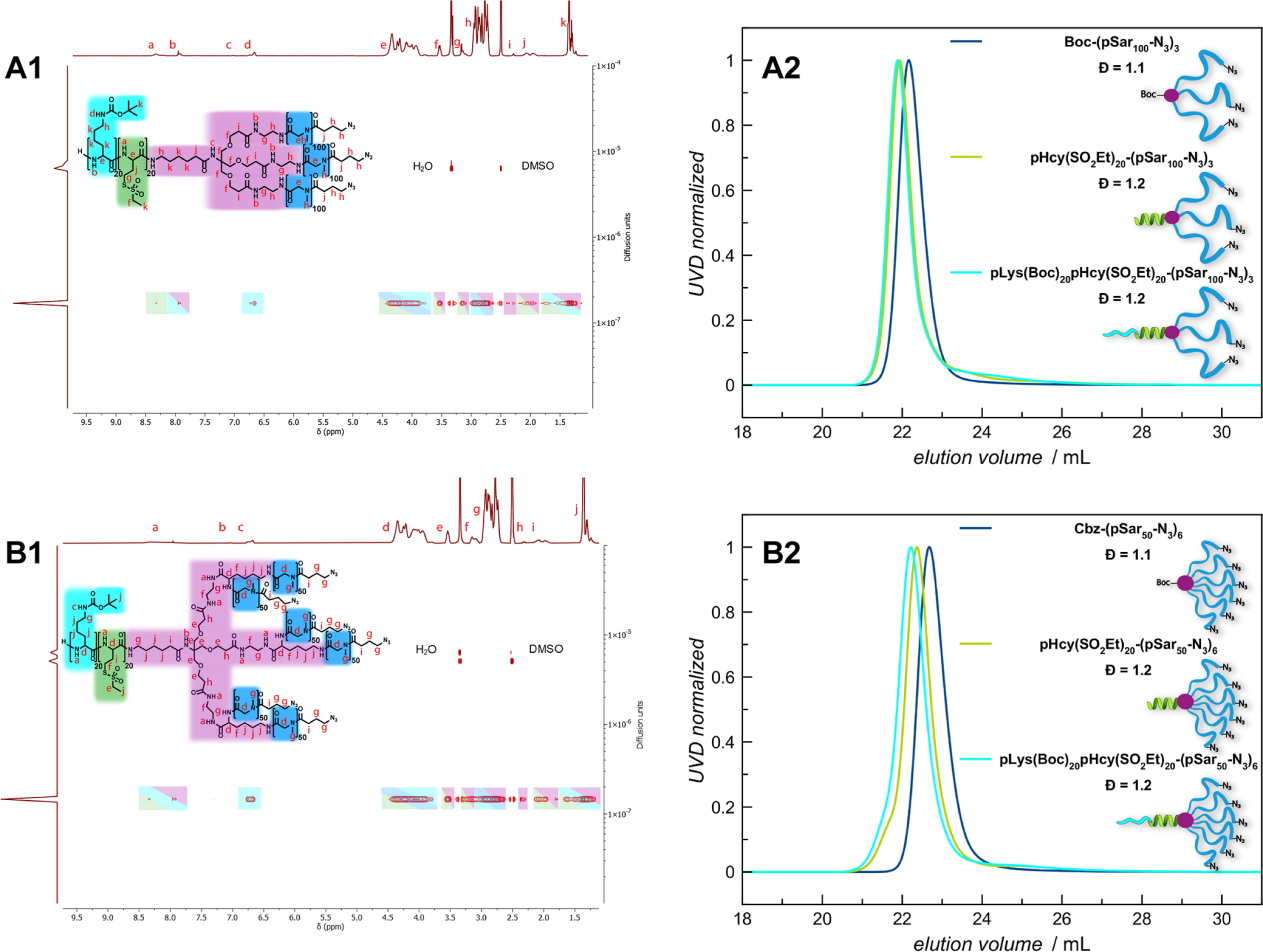
Analyses of the cross-linkable AB_3_ and AB_6_ PeptoMiktoStars after introduction of the pLys(*Boc*) blocks. (A1) ^1^H DOSY NMR spectrum of pLys(*Boc*)_20_pHcy(SO_2_Et)_20_(pSar_100_-N_3_)_3_ in DMSO-*d*_6_. (A2) GPC traces of pLys(*Boc*)_20_pHcy(SO_2_Et)_20_(pSar_100_-N_3_)_3_ in HFIP. (B1) ^1^H DOSY NMR of pLys(*Boc*)_20_pHcy(SO_2_Et)_20_(pSar_50_-N_3_)_6_ in DMSO-*d*_6_. (B2) GPC traces of pLys(*Boc*)_20_pHcy(SO_2_Et)_20_(pSar_50_-N_3_)_6_ in HFIP.

The SEC analysis shown in Figure 3A2,B2, revealed for both macroinitiators that chain growth occurs simultaneously and uniformly, leading to monomodal molecular weight distributions and low dispersities (*D̵* = ∼1.1). Additionally, the ^1^H DOSY experiments provided additional confirmation for both systems, showing that the star-shaped architecture is formed by the integration of all relevant initiator signals into one polymeric species, thus substantiating the absence of linear polymers resulting from impurities (Figures 2B and S23).

Once the successful synthesis of the macroinitiators was validated, the remaining protective groups were removed to facilitate the introduction of the functional polypeptide arm in the subsequent step. For the three-arm macroinitiator, the *Boc* deprotection was achieved using the similar approach, involving quantitative cleavage with a mixture of TFA/H_2_O (1:1) at 0 °C, ensuring its structural integrity.^48^ As previously highlighted in the introduction of the established strategy, the Cbz protecting group can no longer be removed by reductive hydrogenation in the presence of palladium due to steric hindrance.^49^ By implementing the method outlined by Saroha et al., the liberation of the final protected amine functionality at the six-arm macroinitiator was successfully achieved. The well-defined polymer structure was preserved by this innovative approach while facilitating mild Cbz cleavage via in situ generated nickel boride from NaBH_4_ and NiCl_2_·6H_2_O.^50^ Following the different deprotection steps, both macroinitiators underwent purification through subsequent dialysis against sodium hydrogen carbonate solution and Milli-Q water to eliminate any impurities that could initiate themselves or interact with the sensitive functional groups that were later introduced. Moreover, this step obviates the necessity of adding a base in subsequent polymerization steps to ensure the simultaneous initiation of all existing amine functionalities, thereby minimizing the risk of potential side reactions, e.g., homopolymer formation, and enabling the synthesis of well-defined miktoarm stars. The ^1^H NMR and ^1^H DOSY analyses, depicted in Figures S26–S30, provide conclusive evidence for the accomplished deprotections, purity, and preservation of 3-arm and 6-arm macroinitiators.

In accordance with the design concept outlined in Scheme 1 and Figure S1, the prepared precursors were employed for chain extension to incorporate the reactive pHcy(SO_2_Et) block into both star topologies. The polymerization process was conducted following the same procedure as the macroinitiator synthesis, but at a temperature of −10 °C to maintain the cross-linkable sites of the *S*-alkylsulfonyl group during the ROP of the respective NCA.^40,43,44^ Once all monomers for the initial block were consumed, the crude AB_3_ and AB_6_ PeptoMiktoStars were precipitated in diethyl ether and purified by spin-filtration (Amicon Ultra 15, MWCO 10 kDa, 4500 rpm) to remove residual low molecular weight components. As stated before, the sequential polymerization of both polypeptide blocks is straightforward and scalable from several milligrams to hundreds of milligrams. However, as demonstrated, the implementation of an additional purification step enables characterization of each PeptoMiktoStar and enhances the level of control over the final structure and the resulting molecular weight distribution.^49^

Upon successful purification of the star polymers, both were lyophilized and afterward analyzed via ^1^H NMR, ^1^H DOSY, and SEC (Figures S32, 33, S37, and 38). The chain length of the pHcy(SO_2_Et) block was determined using ^1^H NMR spectroscopy by examining the isolated signal of the amide proton at 8.32 ppm and verifying the intended DP_*n*_ of 20 for both miktoarm stars. SEC analysis displayed successful chain extension, as evidenced by a distinct shift of the elugrams toward lower elution volumes while still maintaining monomodal and narrow molecular weight distributions (Figure 3A2,B2). Furthermore, the successful synthesis of both architectures (chain extension) and the absence of homopolymers were confirmed by ^1^H DOSY (Figures S33 and S38). Both spectra show the presence of only one diffusing species, which can be identified as the PeptoMiktoStars. These polymers display the anticipated signals for the respective initiator, the first block of the functional polypeptide arm pHcy(SO_2_Et), and multiple pSar-arms. After effective integration of the reactive pHcy(SO_2_Et) block as the first part of the polypeptide arm into the asymmetric star architectures, the complexing pLys block was introduced to complete the intended AB_3_ and AB_6_ miktoarm star polymers with functional block copolypeptide segment (A). The *Boc*-protected lysine, pLys(*Boc*), enables acidic deprotection, which is compatible with the *S*-alkylsulfonyl protective groups on the polycysteine block. Notably, the use of protective groups cleavable by hard or soft nucleophiles is not compatible with the integrity of the *S*-alkylsulfonyl group.^18,43^

The incorporation of the second polypeptide block was achieved by polymerizing Lys(*Boc*)-NCA, utilizing the preliminary AB_3_ and AB_6_ miktoarm stars as macroinitiators, as illustrated in Scheme 1 and Figure S1, following the same conditions as in the pSar synthesis. After verifying the completeness of monomer consumption via IR, the finalized structures were processed and analyzed in a similar manner to the previous steps, including ^1^H NMR, ^1^H DOSY, and SEC in HFIP (Figures 3, S34 and S39).

Based on the findings presented in Table 1, the targeted chain lengths align remarkably well with the data obtained from ^1^H NMR experiments but are overall slightly smaller than expected. This small deviation is caused by the branched structure of AB_3_ and AB_6_ miktoarm stars related to the linear pSar polymers used for the calibration. Besides, the AB_6_ miktoarm stars display a hydrodynamic volume slightly lower than that of their AB_3_ counterparts. This further reaffirms the importance of macroinitiator purification and underlines that the monomer/macroinitiator ratio is the key element for enabling control over the DP_*n*_. The chain lengths of the respective pLys(*Boc*)-blocks were evaluated by relating the isolated signal of the *Boc* amide proton at 6.66 ppm to the pSar block. The verification for the successful synthesis of cross-linkable AB_3_ (pLys(*Boc*)_20_pHcy(SO_2_Et)_20_(pSar_100_)_3_) and AB_6_ (pLys(*Boc*)_20_pHcy(SO_2_Et)_20_-(pSar_50_)_6_) PeptoMiktoStars, along with the added complexing segment, is supported by the ^1^H DOSY findings depicted in Figure 3A1,B1. For both topologies, only a single diffusing species can be detected, exhibiting a narrow diffusion index distribution with all relevant signals related to the corresponding initiator and the polymer components (pSar, pHcy(SO_2_Et), and pLys(*Boc*)). The SEC elugrams (Figure 3A2,B2) provides further confirmation of successful chain extension, revealing a noticeable shift from the precursor as well as an even greater shift to the respective protected pSar-macroinitiator. In addition, it becomes evident that both synthesized miktoarm star polymers feature monomodal and narrow molecular weight distributions, with low dispersity indices of *D̵* = 1.2 for AB_3_ and *D̵* = 1.2 for AB_6_ topology, thereby verifying the synthesis of the targeted structures as well-defined PeptoMiktoStars.

**Figure 4:**
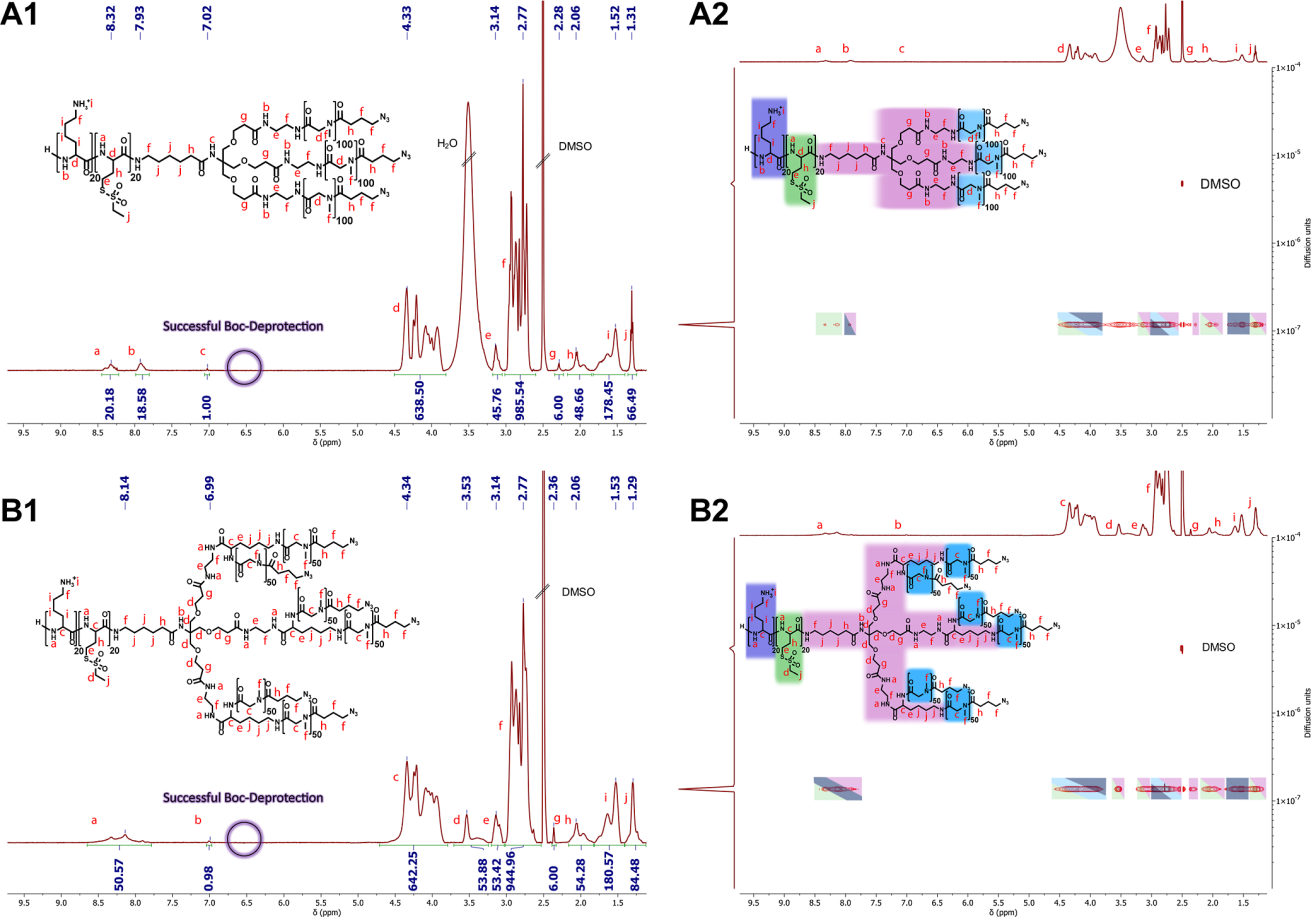
Analysis of the cationic and cross-linkable AB_3_ and AB_6_ PeptoMiktoStars after successful *Boc*-deprotection. (A1) ^1^H NMR spectrum of pLys_20_pHcy(SO_2_Et)_20_(pSar_100_-N_3_)_3_ in DMSO-*d*_6_. (A2) ^1^H DOSY NMR of pLys_20_pHcy(SO_2_Et)_20_(pSar_100_-N_3_)_3_ in DMSO-*d*_6_. (B1) ^1^H NMR of pLys_20_pHcy(SO_2_Et)_20_(pSar_50_-N_3_)_6_ in DMSO-*d*_6_. (B2) ^1^H DOSY NMR of pLys_20_pHcy(SO_2_Et)_20_(pSar_50_-N_3_)_6_ in DMSO-*d*_6_.

It should be noted that at DP_20_ pLys(*Boc*) can adopt either a random coil or helical secondary structure, differing in respective hydrodynamic volume, which artificially broadens SEC plots.^55^ Such effects, however, are hardly visible here due to the large pSar arms. To obtain the desired miktoarm star polymers capable of complexing nucleic acids, it is essential to deprotect the realized AB_3_ and AB_6_ PeptoMiktoStars in the next step. As previously stated, the switch of polarity from a hydrophobic to a cationic core can easily be achieved by acid-mediated removal of the *Boc*-protecting groups, a procedure we have already successfully shown for the linear systems^18,56^ and symmetrical star block copolymers.^27^ The ^1^H NMR analysis revealed that the respective pLys(*Boc*) blocks were successfully deprotected, as indicated by the absence of the *Boc* methyl protons at 1.35 ppm and the *Boc* amide proton at 6.66 ppm (Figure 4). After successful deprotection, the ^1^H DOSY experiment demonstrates that the structural integrity of both systems remains intact. Only one diffusing species can be observed, which still exhibits all relevant signals without any interfering impurities that could hinder the further application process.

### 4.1 Characterization of Nucleic Acid-Loaded PICMs

In previous work, the capacity of linear triblock copolymer micelle formation has been studied in siRNA delivery to Neuro2A and KB cells.^18^ First, we examined the ability of AB_3_ and AB_6_ to complex large nucleic acids, namely, mRNA and pDNA, using agarose gel electrophoresis. Under the applied conditions (HEPES buffer at pH of 7.4), hydrolysis of the *S*-alkylsulfonyl protective group occurs and causes cross-linking by disulfide formation, which stabilizes the PICMs.^43^ As shown in Figure S43A, free pDNA or mRNA was loaded into lane 1 as a control. The bands corresponding to naked pDNA became faint when the N/P ratio increased. The full complexation started from N/P 1 and N/P 2 for AB_3_/pDNA PICMs and AB_6_/pDNA PICMs, respectively (A1, A2). Both AB_3_ and AB_6_ were able to package mRNA and fully complex mRNA at the same N/P ratios observed for pDNA (A3, A4). For a direct comparison, AB_6_ polymers form stable complexes at higher N/P ratios compared to those of AB_3_, which may relate to better steric shielding. The complexes can be destabilized in the presence of glutathione, as demonstrated by Heller et al. and Capelôa et al., where chemoselective disulfide formation was employed for the stabilization of PICMs.^18,41^

We then characterized the different mRNA/AB_*n*_ and pDNA/AB_*n*_ PICMs using DLS, as shown in Figure S43B. PICMs of pDNA/AB_3_ were around 165 nm at N/P = 2, followed by a slight decrease in the hydrodynamic diameter (*D*_h_), along with the increased N/P ratio (B1), indicating a better compaction of the pDNA at higher N/P ratios. A comparable behavior was found for PICMs of pDNA/AB_6_, approximately 180 nm at N/P 2 and 120 nm at N/P 20 (B2). The compaction of pDNA is, however, in both cases less efficient than for linear pSar_300_-*block*-pLys_20_ block ionomers,^46^ which may relate to the increased steric demand at the interface of polypeptide and pSar blocks. In general, the size of PICMs decreased when the N/P ratio increased. The polydispersity observed in the single-angle DLS (Malvern, Zetasizer) measurements was between 0.2 and 0.3, indicating a modest uniformity of PICMs. Therefore, for further studies, the use of microfluidics seems beneficial.

**Figure 5:**
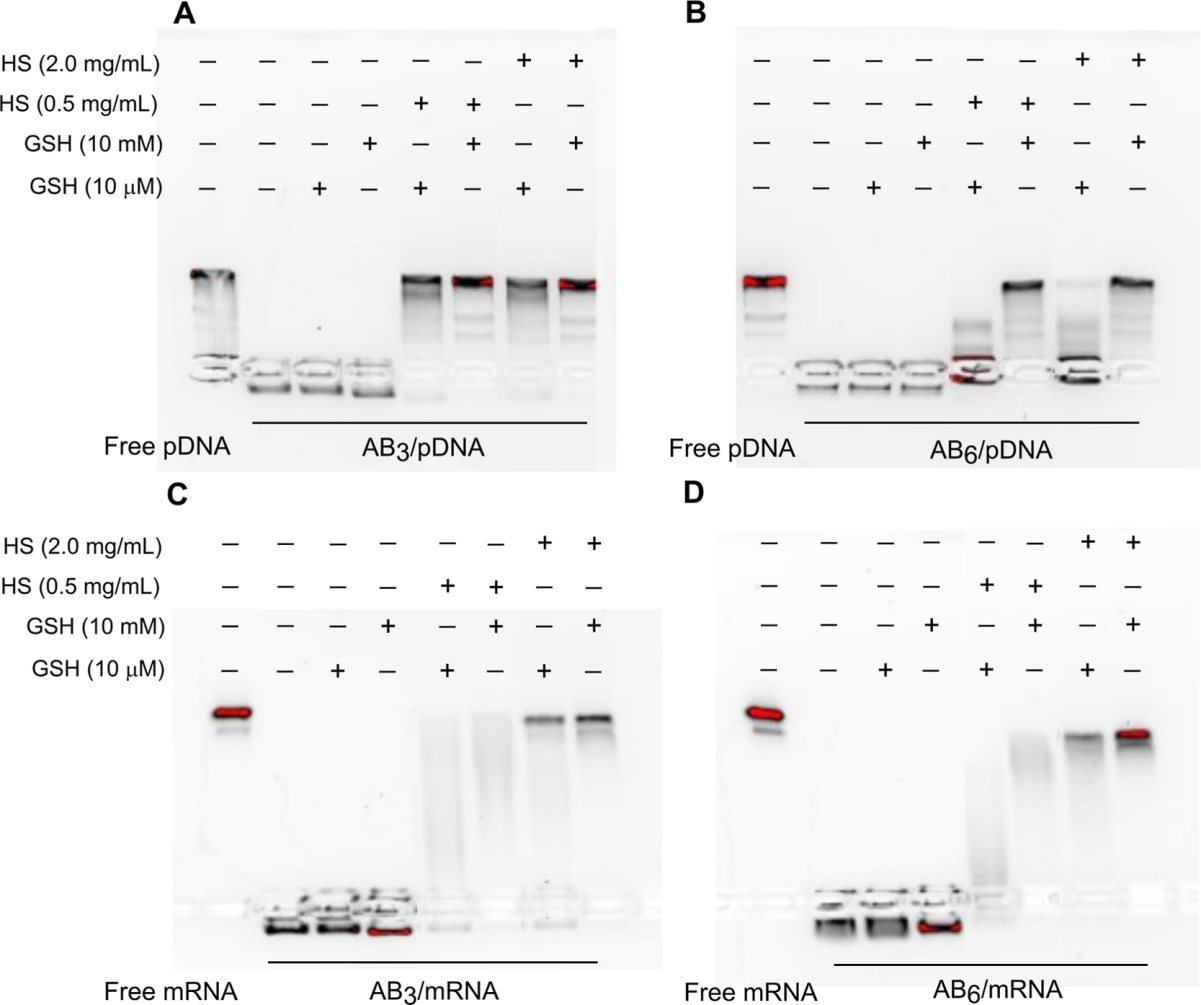
Representative images of (A,B) pDNA and (C,D) mRNA release in the presence of glutathione (GSH) and/or HS. Lane 1, free pDNA or mRNA; lane 2, (A,B) AB_*n*_/pDNA PICMs (N/P 10) or (C,D) AB_*n*_/mRNA PICMs (N/P 10); lane 3, 4, PICMs incubated with GSH at a final concentration of (lane 3) 10 μM or (lane 4) 10 mM; lane 5–6, PICMs incubated with (lane 5) 10 μM or (lane 7) 10 mM GSH, followed by exposure to HS (0.5 mg/mL); lane 7–8, PICMs incubated with (lane 7) 10 μM or (lane 8) 10 mM GSH, followed by exposure to HS (2.0 mg/mL).

Next, we examined the release of pDNA and mRNA from PICMs in dependency on glutathione (GSH) and HS at concentrations of 10 μM (blood level) and 10 mM (intracellular level) for GSH and 0.5 and 2.0 mg/mL for HS by gel electrophoresis (Figure 5). In the presence of GSH at 10 μM and 10 mM, all PICMs remained stable, and differences in pSar grafting density at the polypeptide/polypeptoid interphase were not visible for either mRNA or pDNA. When HS was added to trigger release from PICM by competing with the nucleic acids for polylysine binding, only the AB_6_-miktoarm star polymer-based PICMs containing pDNA remained stable at low GSH and low as well as high HS levels of 0.5 and 2.0 mg/mL, which underlines the more efficient shielding of AB_6_-compared to the AB_3_-miktoarm star polymer. Interestingly, the same systems did only provide modest stability for mRNA-based PICMs. Further research needs to clarify the underlying cause for those distinct differences between mRNA and pDNA.

At high GSH concentrations of 10 mM and high HS levels, all PICMs released their nucleic acid cargo in the gel electrophoresis experiments (Figure 5). Nevertheless, the cross-linking by *S*-alkylsulfonyl cysteine hydrolysis followed by disulfide formation between cysteines leads to less stable PICMs compared to the use of cross-linking agents containing additional amine moieties for electrostatic interaction with nucleic acids.^41^ This data indicates that indeed the stability of PICMs depends on the grafting density of pSar chains at the polypeptide/polypeptoid interphase, since only the AB_6_-miktoarm star polymer provides the expected stability.

### 4.2 AB_3_ Exhibited More Efficient Nucleic Acid Delivery In Vitro

In vitro cell study was conducted using a suspended human T lymphocyte cell line (Jurkat T) and adherent dendritic (D1) cell lines to evaluate uptake and transgene expression. To quantify the cellular uptake and transfection efficiency, Alexa Fluoro 647 (AF647) labeled-AB_*n*_ polymers were used to complex GFP encoding pDNA or mRNA into PICMs. Both the fraction of fluorescent positive cells and mean fluorescence intensity (MFI) were determined to evaluate the cellular uptake and transfection efficiency.

After being exposed to pDNA PICMs at a relatively high concentration of 100 μg/mL for 24 h, approximately 100% of cells exhibited AF647-positive, regardless of the formulation and cell type (Figure S44). The cytotoxicity was found to be cell- and dose-dependent. D1 cells were more resistant to the polymers, and AB_6_ is more toxic to Jurkat T cells than AB_3_ (Figure S47). Overall, both D1 and Jurkat T cells tolerated AB_3_ and AB_6_ polymers well within the dose applied for transfection in this study. Initial indications for cellular toxicity were first observed at doses of 5-fold above the highest concentration applied in this study.

**Figure 6:**
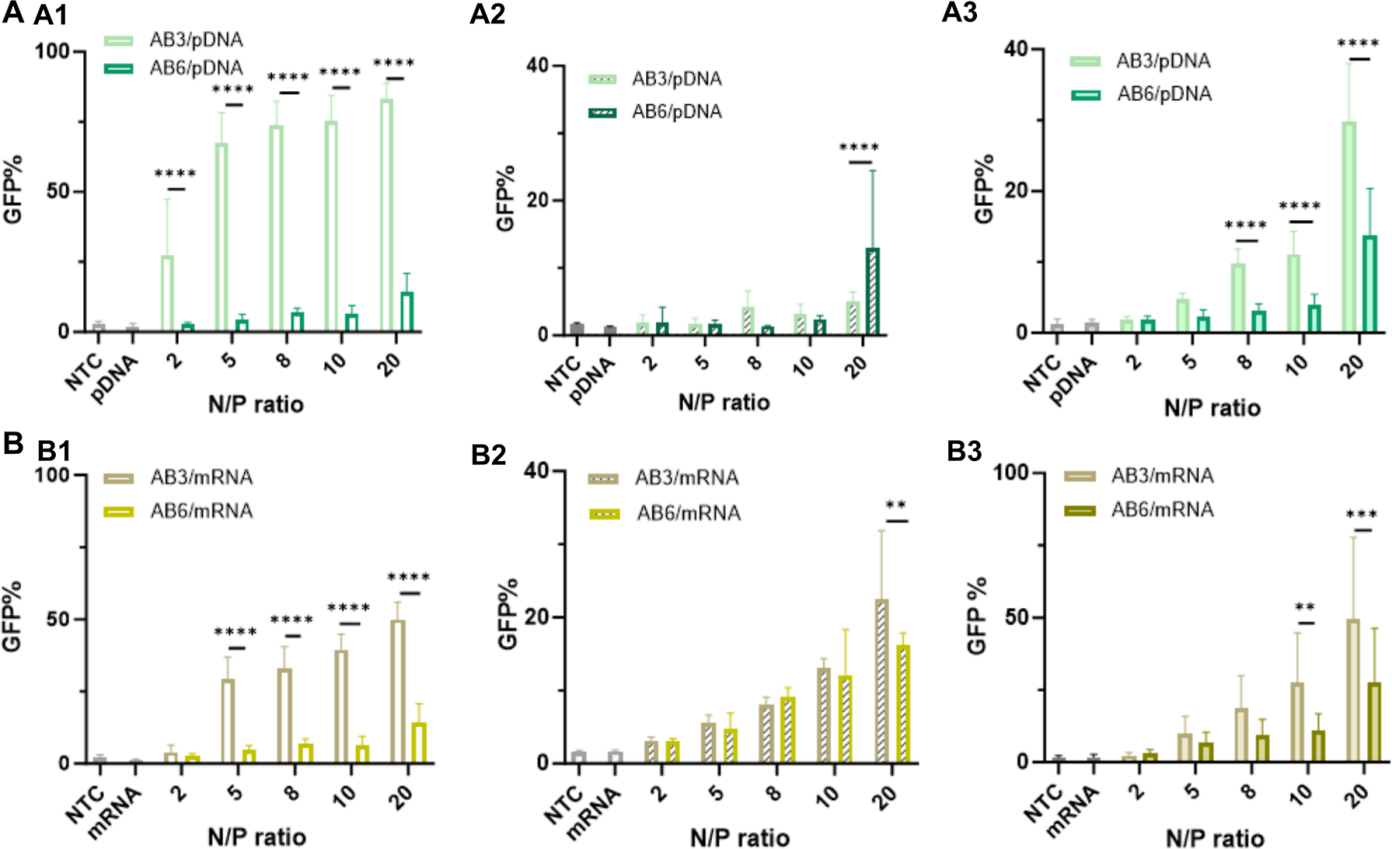
In vitro biological performance of (A) pDNA/ABn PICMs and (B) mRNA/ABn PICMs on (A1,B1) D1 and (A2,B2,A3,B3) Jurkat T cells. (A1,B1) In vitro evaluation of (A1) pDNA/ABn PICMs and (B1) mRNA/ABn PICMs at varying N/P ratios as indicated after being exposed to D1 cells in complete culture medium, respectively. (A2,B2; A3,B3) In vitro evaluation of (A2) pDNA/ABn PICMs and (B2) mRNA/ABn PICMs at varying N/P ratios as indicated after being exposed to Jurkat T cells cultured (A2,B2) in complete culture medium and in (A3,B3) Opti-MEM, respectively. Nontreated cells (NTC) and naked pDNA or mRNA served as controls. In each well, the final pDNA or mRNA concentration is 0.2 μg/well. At the end of incubation, cells were collected for flow cytometry measurement. All the data was averaged from three independent experiments (*n* ≥ 9). Statistical analysis was performed by 2-way ANOVA with the software GraphPad Prism 10 (*p < 0.05; **p < 0.01; ***p < 0.001, ****p < 0.0001).

Next, the expression of GFP encoded on pDNA (GFP) and mRNA (EGFP) constructs was investigated in D1 and Jurkat cells. Surprisingly, this cell-associated fluorescence did not lead to pronounced GFP expression. In the case of adherent D1 cells, the two treatments diverged significantly in the fraction of GFP (%)-positive cells. When cells were treated with pDNA/AB_3_ PICMs, a substantial increase in GFP (%) was detected as the N/P ratio increased, ranging from 67% (N/P 5) to 83% (N/P 20). While only up to 14% GFP-positive cells were detected after incubation with pDNA/AB_6_ PICMs at N/P 20 (Figure 6A1). A significantly higher GFP-associated fluorescence (rMFI) was found in AB_3_-based PICMs-treated D1 cells from N/P ratios 5 to 20 for both pDNA and mRNA (Figure S45A1,B1). The capacity of AB_*n*_ polymers to deliver pDNA in vitro was further studied in suspended Jurkat T cells. Only 5% and 12% GFP cells were detected in the cells exposed to pDNA/AB_3_ and pDNA/AB_6_ PICMs at N/P 20, respectively (Figure 6A2). As expected, the rMFI values in Jurkat T cells across all formulations in this study indicate very limited protein production (Figure S45A2).

We also studied the transfection efficiency of AB_3_- and AB_6_-based PICMs for mRNA. In terms of GFP-positive cells (%), mRNA/AB_3_ PICMs-treated D1 cells exhibited 30% at N/P 5 and gradually reached 50% at N/P 20, while cells exposed to mRNA/AB_6_ PICMs displayed a substantially lower GFP (%), up to 14% at N/P 20 (Figure 6B1). Again, GFP rMFI was higher for mRNA/AB_3_ PICMs than that of mRNA/AB_6_ (Figure S45B1). Compared with mRNA, the AB_3_ polymer was more efficient in inducing GFP expression by delivering pDNA into D1 cells with respect to GFP (%). When Jurkat T cells were exposed to mRNA/AB_3_ PICMs at N/P 10 and 20, 14% and 20% GFP (%) were determined, respectively. Whereas exposure to mRNA/AB_6_ PICMs showed less than 10% of GFP (%) at N/P from 2 to 8 and slightly increased to 16% at N/P 20 (Figure 6B2). For both cases, there was no increase in the GFP rMFI compared to the control groups regardless of treatments (Figure S45B2), which indicates low expression levels. In summary, AB_3_-based PICMs demonstrated a higher transfection efficiency in D1 cells compared to the AB_6_-based counterparts, regardless of the nucleic acid cargo, while all treatments induced rather limited transfection efficiency on Jurkat T cells. This may be attributed to a more pronounced shielding effect of AB_6_ compared to AB_3_–PICMs, which seems to limit endosomal escape and disassembly of PICMs more significantly than cellular uptake. Besides, pLys is known to have limited endosomal escape properties and enhanced binding to pDNA and mRNA.

To study the influence of serum proteins on transfection efficiency, Opti-MEM, which contains reduced serum, was used to replace the complete culture medium. Again, almost 100% AF647-positive Jurkat T cells were detected upon all treatments (Figure S44A3). Only a slightly higher transfection efficiency was observed after treatment with pDNA/AB_3_ PICMs from N/P 5 onward, up to 30% GFP (%), while no significant difference was determined in the cells exposed to pDNA/AB_6_ PICMs (Figure 6A3), which underlines the effective shielding of PICMs by the 6-arm miktoarm star polymer. We also examined the effect of Opti-MEM on the transfection efficiency of AB_3_- and AB_6_-based PICMs carrying mRNA on Jurkat T cells (Figure 6B3). Similarly, the culture condition did not lead to any changes in the AF647 (%) (Figure S44B3). Around 13%, 38%, and 68% GFP-positive Jurkat T cells were determined after being exposed to mRNA/AB_3_ PICMs at N/P 5, 10, and 20, respectively, whereas maximal 20% in complete culture medium was achieved at N/P 20. As well, mRNA/AB_6_ PICM treatment led to around 22% upon N/P 20 (Figure 6B3).

Further, we validated the findings on D1 cells. Similar results were obtained in Opti-MEM treatment with culture medium, with nearly 100% AF647-positive D1 cells detected among all treatments (Figure S46A1,B1). When cells were exposed to pDNA/AB_*n*_ PICMs, it is indeed a surprise that Opti-MEM treatment showed a slight decrease in GFP-positive cells (%). Being exposed to pDNA/AB_3_ PICMs at N/P 5 and 20, 22%, and 50% GFP (%) was detected in Opti-MEM (Figure S46A2), which was lower than that of 70% and 80% in full culture medium, respectively (Figure 6A1). After being exposed to mRNA/AB_3_ PICMs, GFP (%) substantially increased to approximately 65% at N/P 5 and finally achieved 85% at N/P 20 (Figure S46B2). The enhanced transfection efficiency was not observed in the cells treated with mRNA/AB_6_ PICMs (Figure 6B1).

Therefore, we can conclude that the effect of serum proteins on transfection efficacy of PICMs based on PeptoMiktoStars is minor, which underlines their high stability in complex environments. This stability vice versa limits their efficacy of pDNA and mRNA translation into proteins by reducing endosomal escape and cytosolic nucleic acid release. However, we have recently demonstrated how endosomal escape can be improved substantially by incorporation of endosomolytic molecules, such as cationic amphiphilic drugs (CADs) or viral derived peptides.^41,57,58^ Introducing stability for active targeting with ligands for cell surface proteins, however, is intrinsically complex, which limits nucleic acid delivery to other organs than lung, liver, and spleen. Having demonstrated a promising strategy to achieve stable PICMs in this work, future research will be devoted to the incorporation of ligands and CADs to unleash the full potential of PICMs based on PeptoMiktoStars.

## 5 Conclusions

In this study, we successfully developed novel AB_3_ and AB_6_ PeptoMiktoStars based on polypept(o)ides for large nucleic acid, pDNA, and mRNA delivery. We applied our recently established approach to introduce polylysine (pLys) as a nucleic acid complexing block in the AB_3_ architecture of *S*-alkylsulfonyl homocysteine-(pHcy(SO_2_Et)) and pSar-based miktoarm star polymers. Additionally, we present a new synthetic pathway that allows the attachment of ligands to the surface of AB_3_ and AB_6_ miktoarm stars by azide alkyne click chemistry. Utilizing these methods, we realized well-defined and cross-linkable AB_3_ and AB_6_ PeptoMiktoStars, characterized by narrow molecular weight distributions, low dispersity indices (*D̵* ≤ 1.2), and precise control over the final polymer architecture. Furthermore, we showcase the complexation, stability, and delivery capacity of AB_3_ and AB_6_ for large nucleic acids in terms of in vitro. Both copolymers were able to complex pDNA or mRNA into PICMs, which are stabilized by disulfide formation in the homocysteine block occurring upon hydrolysis in buffer. Interestingly, only AB_6_-miktoarm star polymers provided modest (mRNA) to high (pDNA) stability against 10 μM glutathione and 0.5 and 2.0 mg/mL of HS, while AB_3_-based PICMs are unstable at 2.0 mg/mL of HS. All PICMs are able to deliver their nucleic acid cargo into D1 cells and Jurkat T cells. The transfection efficiency, or GFP (%) is cell- and formulation-dependent. The transgene expression was dose dependent and significantly less efficient in the suspension Jurkat T cells (up to 50%) than the D1 cells (up to 80%). Overall, AB_3_ exhibited a more pronounced transfection efficiency than AB_6_ counterparts, and pDNA led to more robust transfection efficiency than mRNA. While the use of miktoarm star polymers with 3 and especially 6 arms enhances PICM stability, it limits the expression of GFP encoded on pDNA or mRNA. Nevertheless, these limitations can be tackled by the incorporation of endosomolytic molecules, e.g., CADs or viral peptides. Together with suitable ligands to foster interactions with specific cell types, this strategy holds the potential to access PICMs for organ- or even cell-specific nucleic acid delivery.

## Supporting Information Available

NMR data of all initiators, monomers, further reagents, macroinitiators, and final miktoarm star polymers, additional synthesis schemes to illustrate the synthetic approaches, characterization of pDNA/AB_*n*_ and mRNA/AB_*n*_ PICMs including complexation, size and size distribution, and in vitro evaluation and cell viability (PDF)
