# Supplementary material for: Miktoarm Star-polypept(o)ide-Based Polyion Complex Micelles for the Delivery of Large Nucleic Acids
Source: Biomacromolecules. 2024 Sep 18;25(10):6539–54. doi: 10.1021/acs.biomac.4c00695 (PMC11480978; doi:10.1021/acs.biomac.4c00695)
Supplement: Supplementary file 1 — bm4c00695_si_001.pdf [file bm4c00695_si_001.pdf]

Miktoarm star-polypept(o)ide-based polyion complex micelles for the delivery of large nucleic acids

David Schwiertz[1,2#], Jennifer Angelina[1#], Heyang Zhang[1], Matthias Barz[1,2,*]*

[1]Biotherapeutics Division, Leiden Academic Centre for Drug Research (LACDR), Leiden University, Einsteinweg 55, 2333 CC Leiden, The Netherlands

[2]Department of Dermatology, University Medical Center, Johannes Gutenberg-University Mainz (JGU), Obere Zahlbacher Straße 63, 55131 Mainz, Germany

Corresponding author

Matthias Barz: m.barz@lacdr.leidenuniv.nl, Heyang Zhang: h.zhang@lacdr.leidenuniv.nl

D.S and J.A equally contributed to this work.

Table of Content

Syntheses 3

Monomers 4

Sarcosine-N-carboxyanhydride (Sar-NCA) 4

S-Ethylsulfonyl-L-homocysteine-N-carboxyanhydride (Hcy(SO_2Et)-NCA) 5

N-ε-tert-butyloxycarbonyl-L-lysine-N-carboxyanhydride (Lys(Boc)-NCA) 6

Initiators 7

Tetrafunctional Initiator 7

Heptafunctional Initiator 14

Initiator Deprotection for Macroinitiator Synthesis 20

Polypeptoid Macroinitiators 22

Three-Arm Macroinitiator Synthesis (Boc-($pSar_{100}$-N_3)$_3$) 22

Six-Arm Macroinitiator Synthesis (Cbz-($pSar_{50}$-N_3)$_6$) 24

Boc-Deprotection of the Three-Arm Macroinitiator (NH_2-($pSar_{100}$-N_3)$_3$) 25

Cbz-Deprotection of the Six-Arm Macroinitiator (NH_2-($pSar_{50}$-N_3)$_6$) 28

Cross-linkable and Complexing Miktoarm Star Polymers (PeptoMiktoStars) 30

AB_3 PeptoMiktoStar (pLys(Boc)$_{20}$pHcy(SO_2Et)$_{20}$($pSar_{100}$-N_3)$_3$) 31

Deprotected AB_3 PeptoMiktoStar ($pLys_{20}$pHcy(SO_2Et)$_{20}$($pSar_{100}$-N_3)$_3$) 35

AB_6 PeptoMiktoStar (pLys(Boc)$_{20}$pHcy(SO_2Et)$_{20}$($pSar_{50}$-N_3)$_6$) 36

Deprotected AB_6 PeptoMiktoStar ($pLys_{20}$pHcy(SO_2Et)$_{20}$($pSar_{50}$-N_3)$_6$) 40

Further Reagents 41

Pentafluorophenyl-4-azidobutanoat 41

Biological test 43

Synthesis

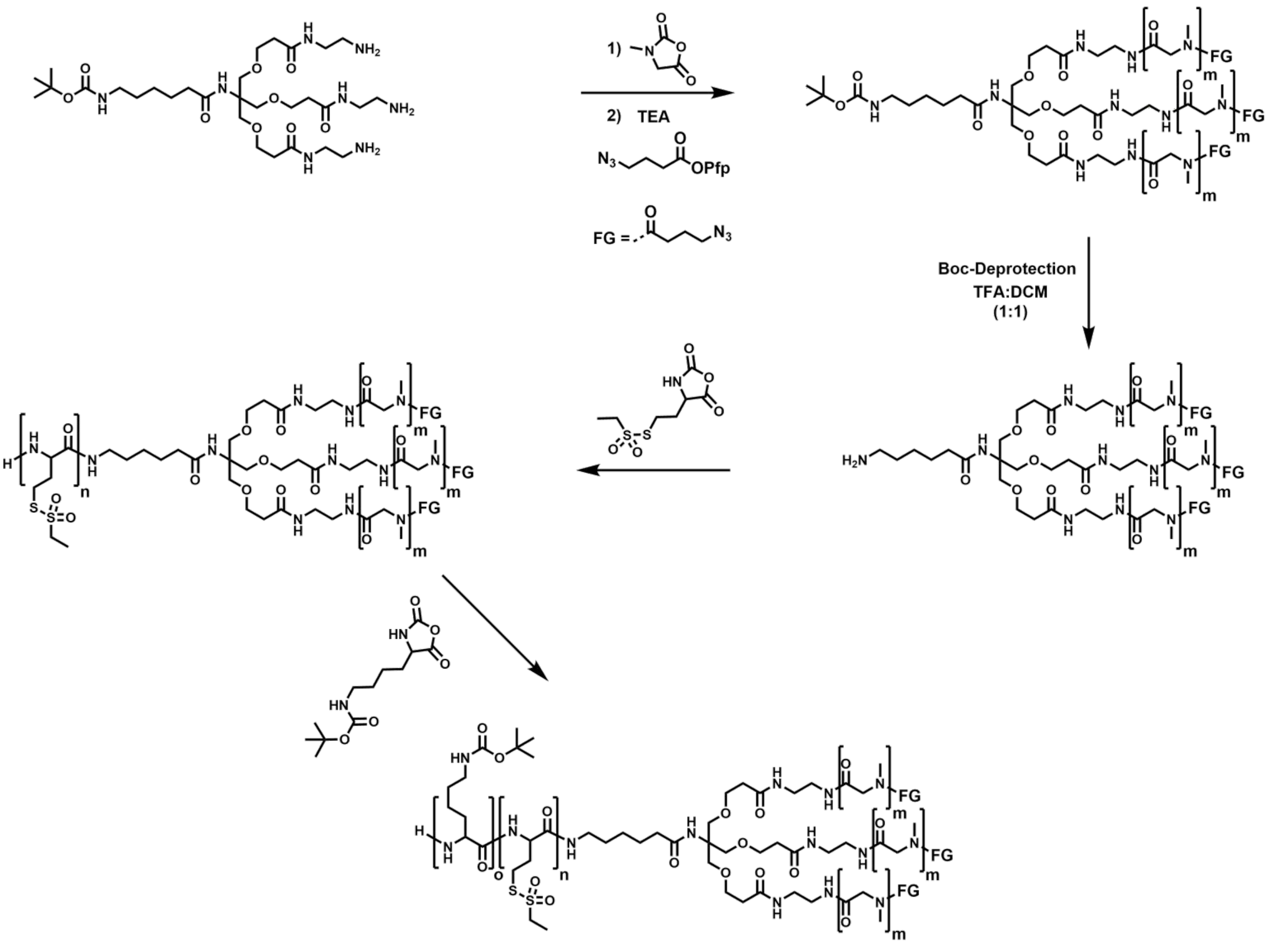

Figure S1. Synthetic pathway for the realization of cross-linkable AB_3 PeptoMiktoStars.

Monomers

Sarcosine-N-carboxyanhydride (Sar-NCA)

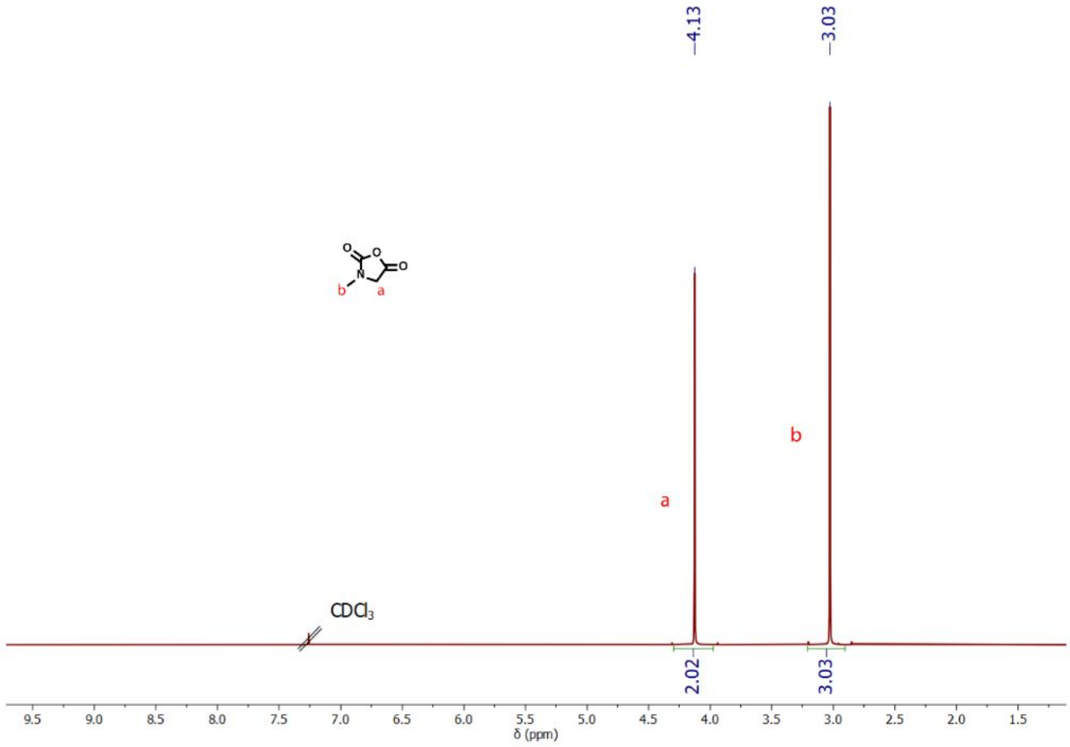

Figure S2. ^{1}H NMR spectrum of sarcosine-*N*-carboxyanhydride (Sar-NCA) in $CDCl_3$.

S-Ethylsulfonyl-L-homocysteine-N-carboxyanhydride (Hcy(SO_2Et)-NCA)

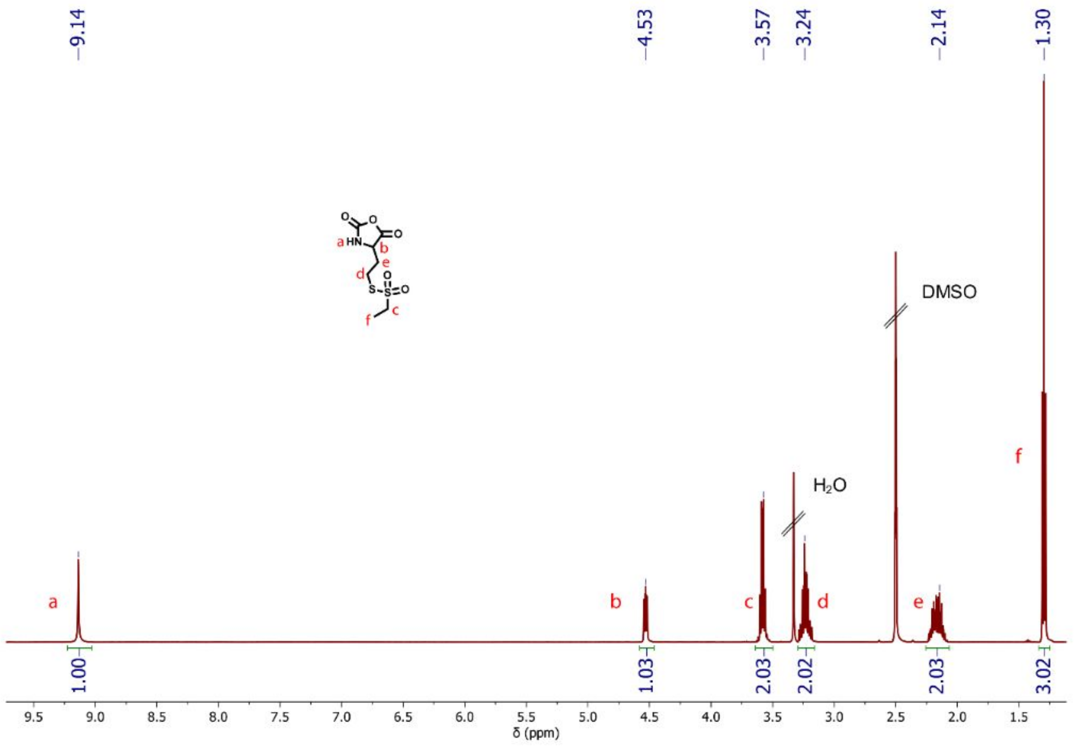

Figure S3. ^{1}H NMR spectrum of *S*-ethylsulfonyl-L-homocysteine-*N*-carboxyanhydride (Hcy(SO_2Et)-NCA) in DMSO-d_6.

***N*-ε-*tert*-butyloxycarbonyl-L-lysine-*N*-carboxyanhydride (Lys(Boc)-NCA)**

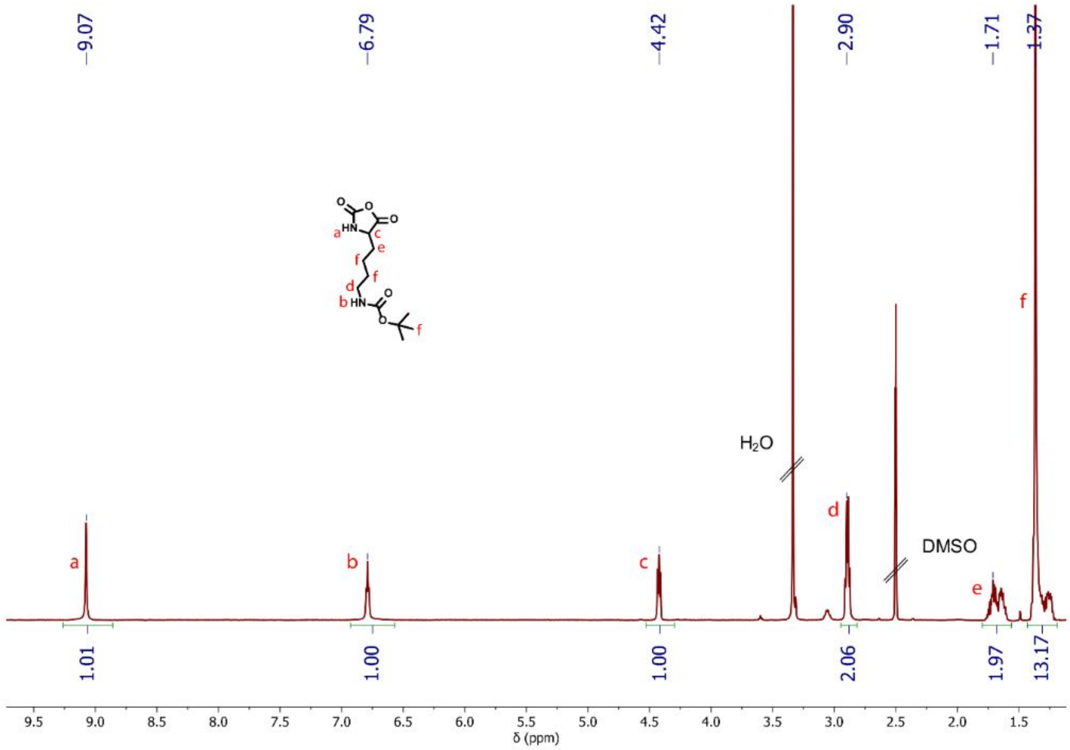

Figure S4. ^{1}H NMR spectrum of *N*-ε-*tert*-butyloxycarbonyl-L-lysine-*N*-carboxyanhydride (Lys(Boc)-NCA) in DMSO-d_6.

Initiators

Tetrafunctional Initiator

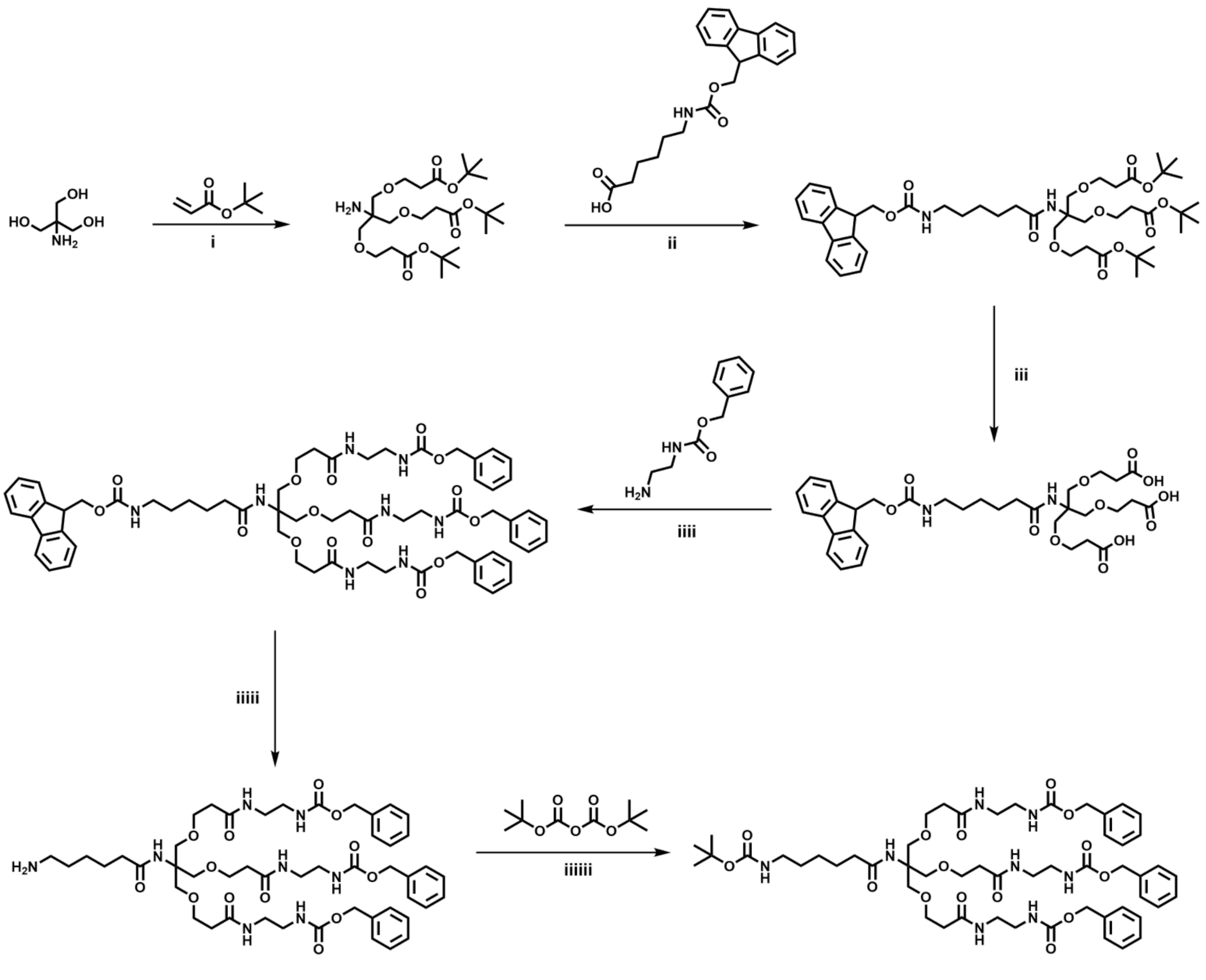

Figure S5. Synthesis of the tetrafunctional initiator Boc-Ahx-Tris{[2-(*N*-Cbz-ethylendiaminecarbonyl)ethoxy]methyl}methyl-amide. i) NaOH, DMSO, 15 °C, 24 h, yield 30 %. ii) PyBOP, DIPEA, THF, rt, yield: 93 %. iii) TFA/DCM (1/1), 0 °C, 1 h, yield: quantitative. iiii) PyBOP, DIPEA, DMF/DCM (1/1), rt, 24 h, yield: 90 %. iiiii) DMF/Piperidine (4/1), 0 °C, 1 h, yield: quantitative, iiiiii) TEA, DCM, rt, 24 h, yield: 92 %.

Synthesis of Tris{[2-(tert-butoxycarbonyl)ethoxy]methyl}methylamine (i)

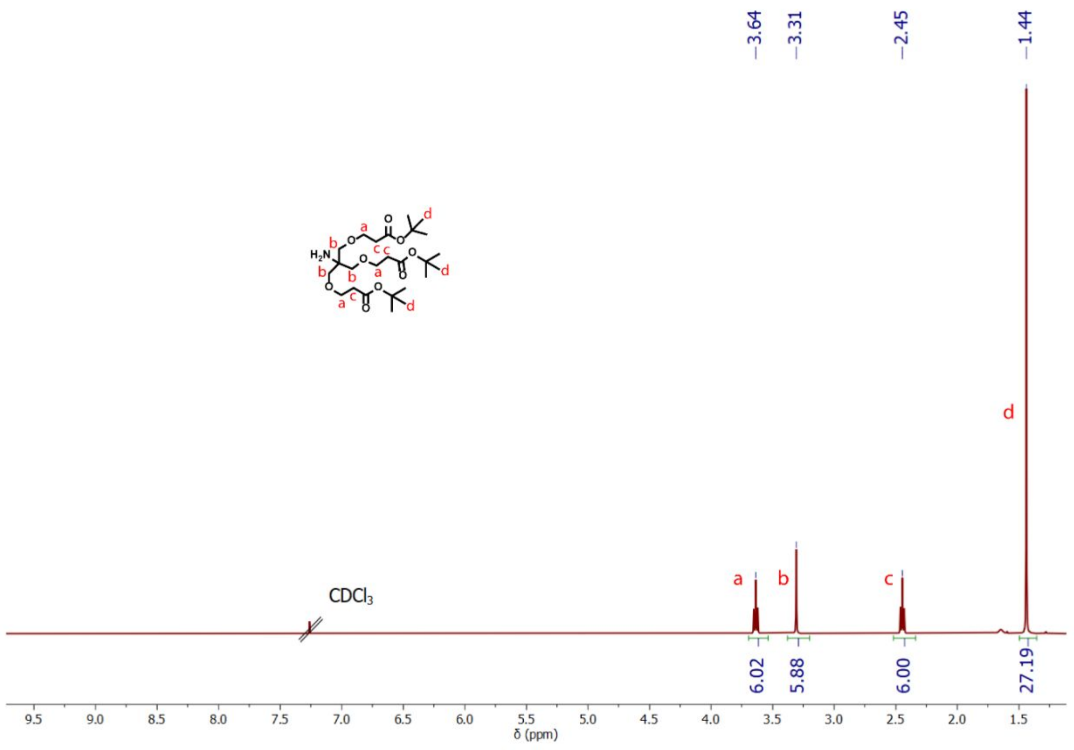

Figure S6. ¹H NMR spectrum of (i) Tris{[2-(*tert*-butoxycarbonyl)ethoxy]methyl}methylamine in $CDCl_3$.

Synthesis of Fmoc-Ahx-Tris{[2-(tert-butoxycarbonyl)ethoxy]methyl}methylamide (ii)

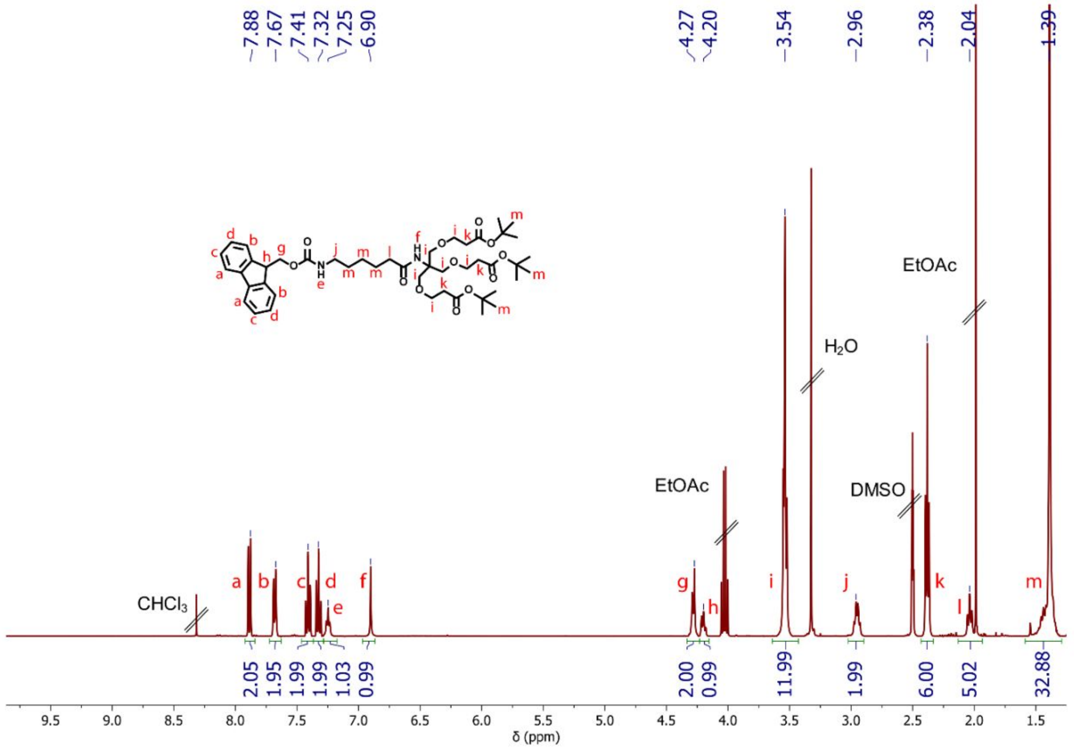

Figure S7. ^{1}H NMR spectrum of (ii) Fmoc-Ahx-Tris{[2-(tert-butoxycarbonyl)ethoxy]methyl}methylamide in DMSO-d_6.

Synthesis of Fmoc-Ahx-Tris[2-(carboxyethoxy)methyl]methylamide (iii)

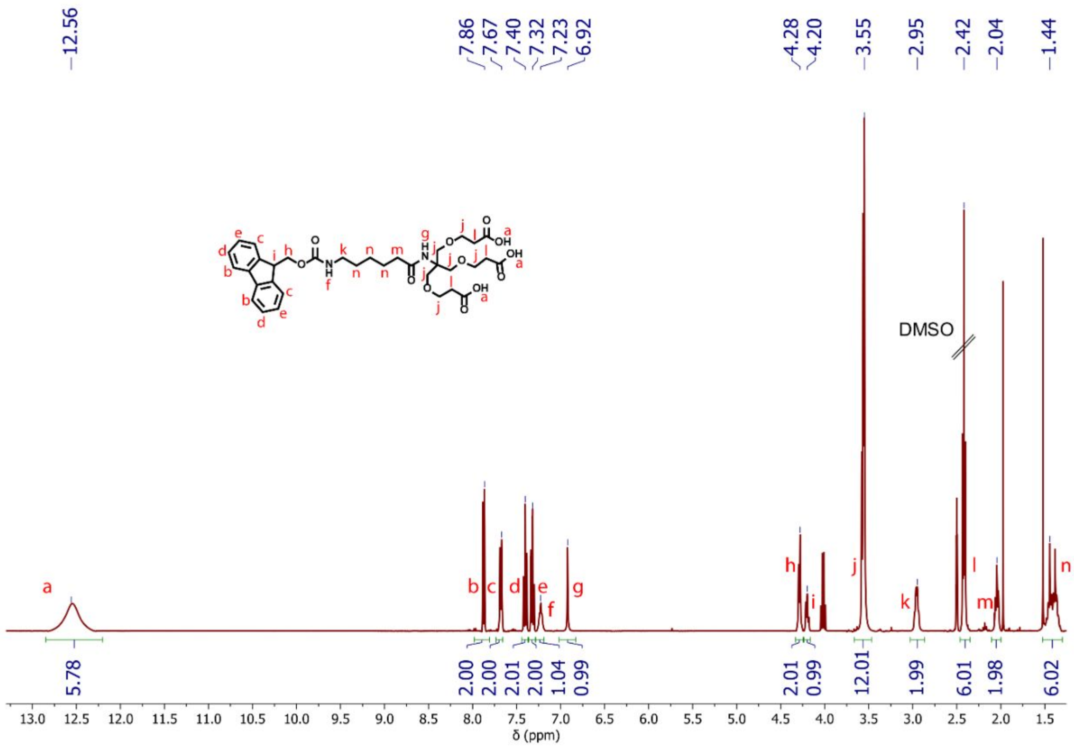

Figure S8. ^{1}H NMR spectrum of (iii) Fmoc-Ahx-Tris[2-(carboxyethoxy)methyl]methylamide in DMSO-d_6.

Synthesis of Fmoc-Ahx-Tris{[2-(N-Cbz-ethylendiaminecarbonyl)ethoxy]methyl}methylamide (iiii)

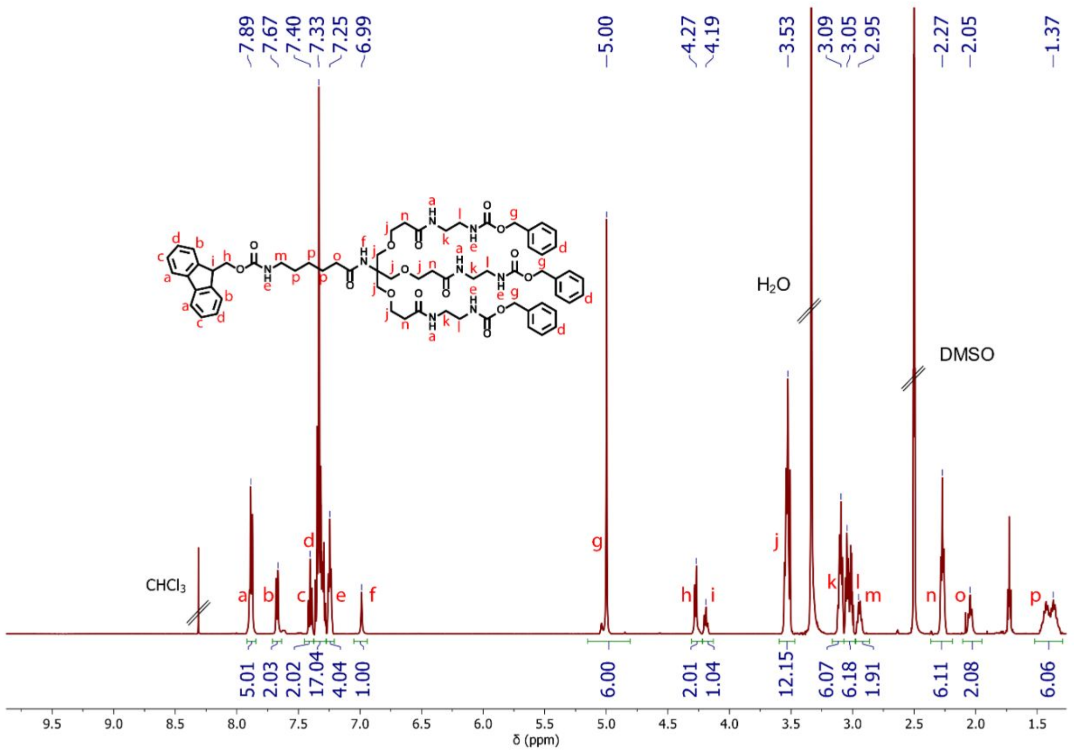

Figure S9. ^{1}H NMR spectrum of (iiii) Fmoc-Ahx-Tris{[2-(*N*-Cbz-ethylendiaminecarbonyl)ethoxy]methyl}methylamide in DMSO-d_6.

Synthesis of Ahx-Tris{[2-(N-Cbz-ethylendiaminecarbonyl)ethoxy]methyl}methylamide (iiiii)

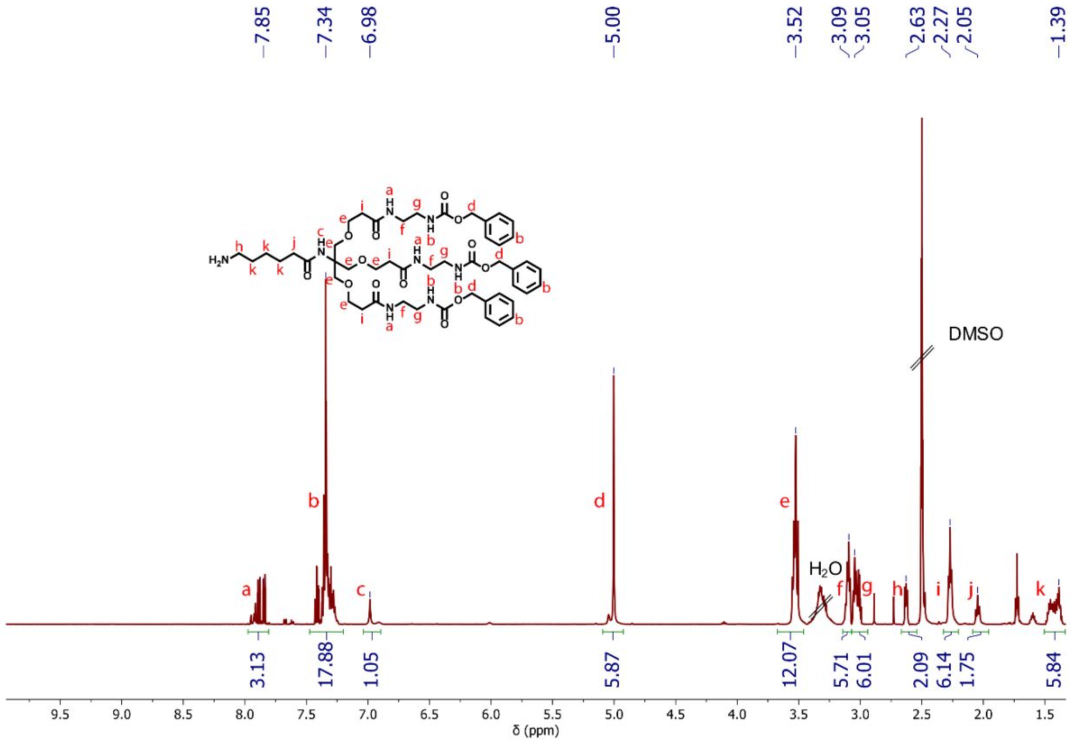

Figure S10. ^{1}H NMR spectrum of (iiiii) Ahx-Tris{[2-(*N*-Cbz-ethylendiaminecarbonyl)ethoxy]methyl}methylamide in DMSO-$\boldsymbol{d_6}$.

Synthesis of Boc-Ahx-Tris{[2-(N-Cbz-ethylendiaminecarbonyl)ethoxy]methyl}methylamide (iiiiii)

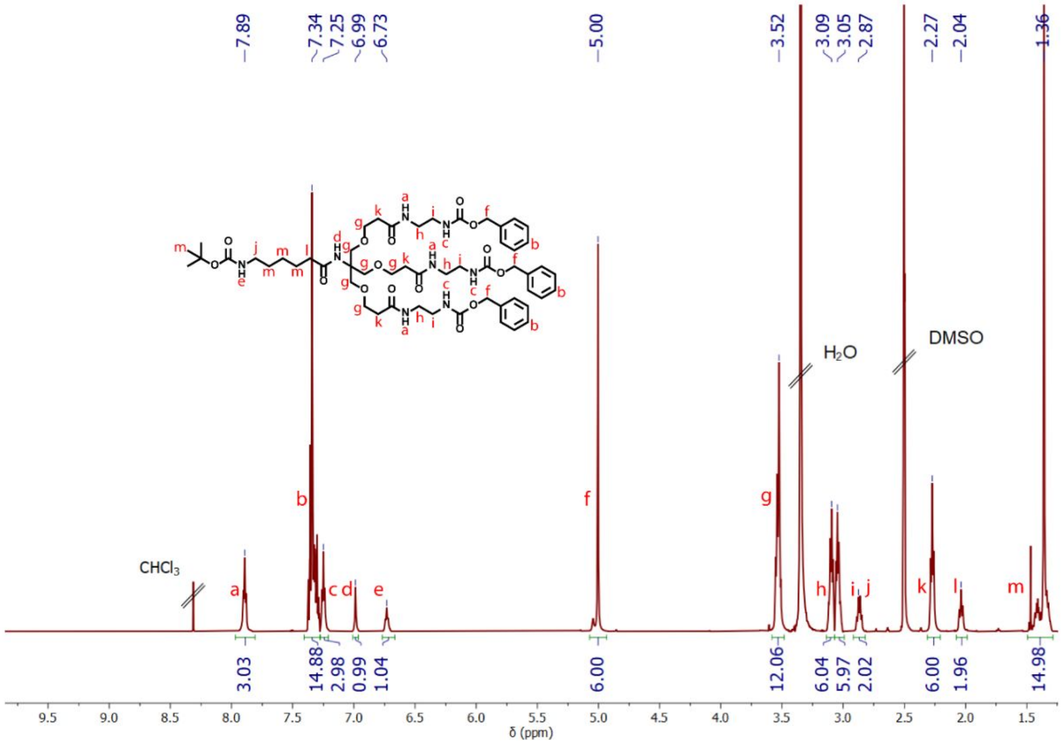

Figure S11. ^{1}H NMR spectrum of iiiiii) Boc-Ahx-Tris{[2-(*N*-Cbz-ethylendiaminecarbonyl)ethoxy]methyl}methylamide) in DMSO-d_6.

Heptafunctional Initiator

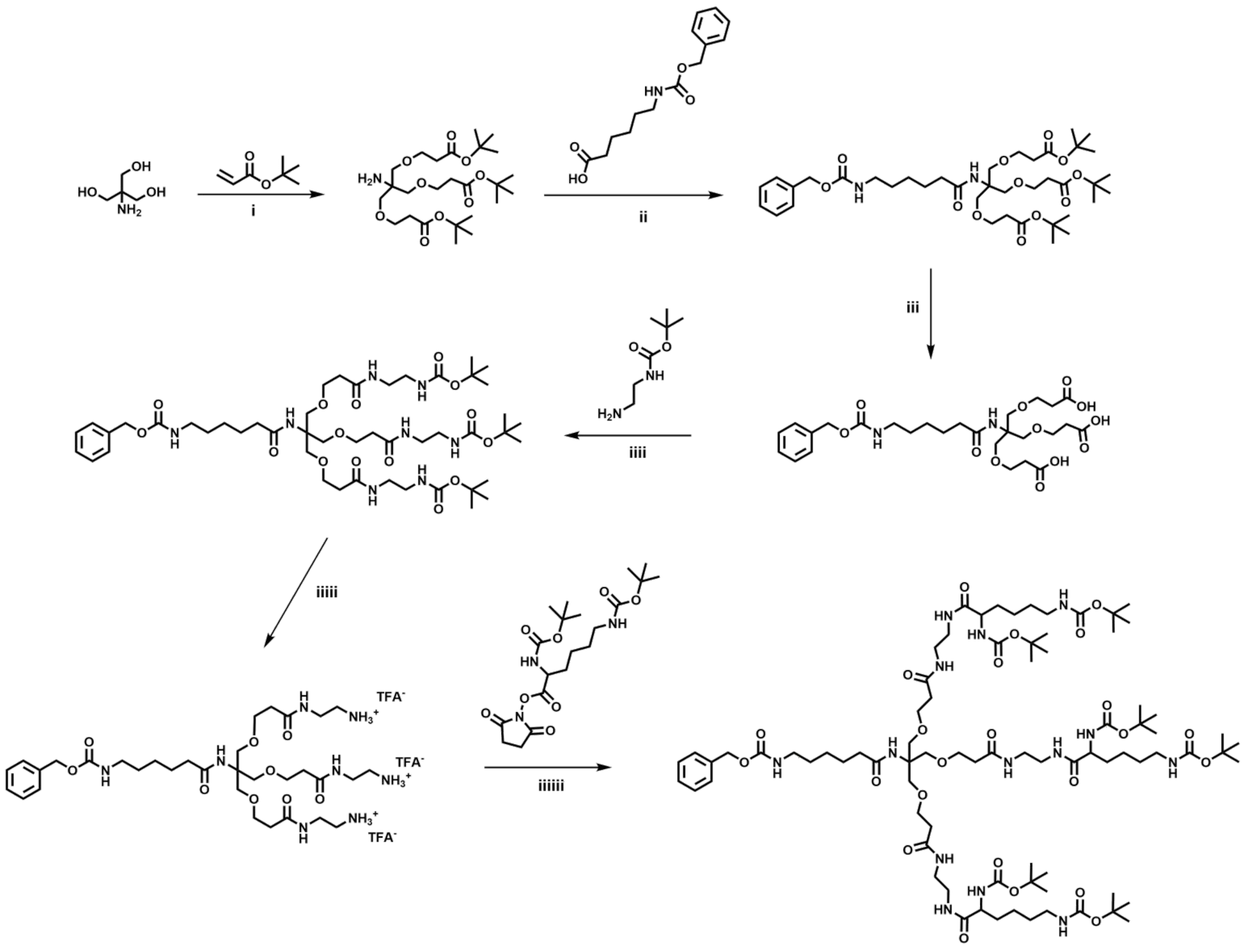

Figure S12. Synthesis of the Heptafunctional initiator. i) NaOH, DMSO, 15 °C, 24 h, yield 30 %. ii) PyBOP, DIPEA, THF, rt, yield: 91 %. iii) TFA/DCM (1/1), 0 °C, 1 h, yield: quantitative. iiii) PyBOP, DIPEA, DMF/DCM (2/1), rt, 24 h, yield: 55 %. iiiii) TFA/DCM (1/1), 10 °C, 1 h, yield: quantitative. iiiiii) DIPEA, DCM/DMF (1/1), rt, 24 h, yield: 63 %.

Synthesis of Cbz-Ahx-Tris{[2-(tert-butoxycarbonyl)ethoxy]methyl}methylamide (ii)

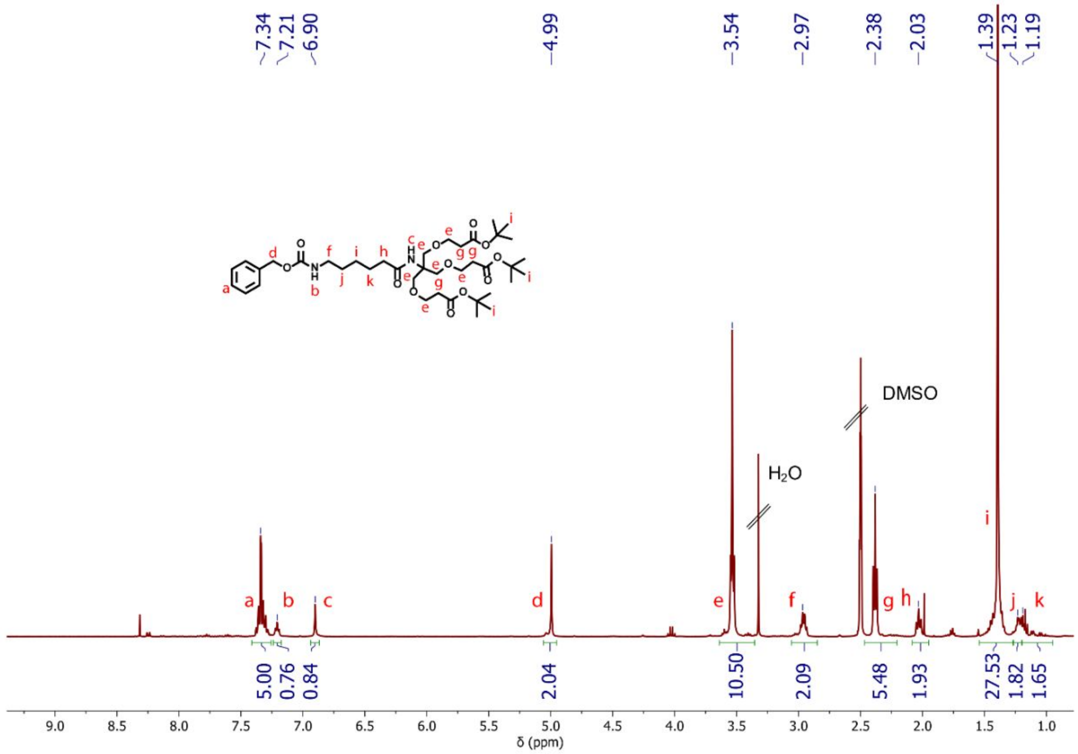

Figure S13. ^{1}H NMR spectrum of (ii) Cbz-Ahx-Tris{[2-(*tert*-butoxycarbonyl)ethoxy]methyl}methylamide in DMSO-d_6.

Synthesis of Cbz-Ahx-Tris[2-(carboxyethoxy)methyl]methylamide (iii)

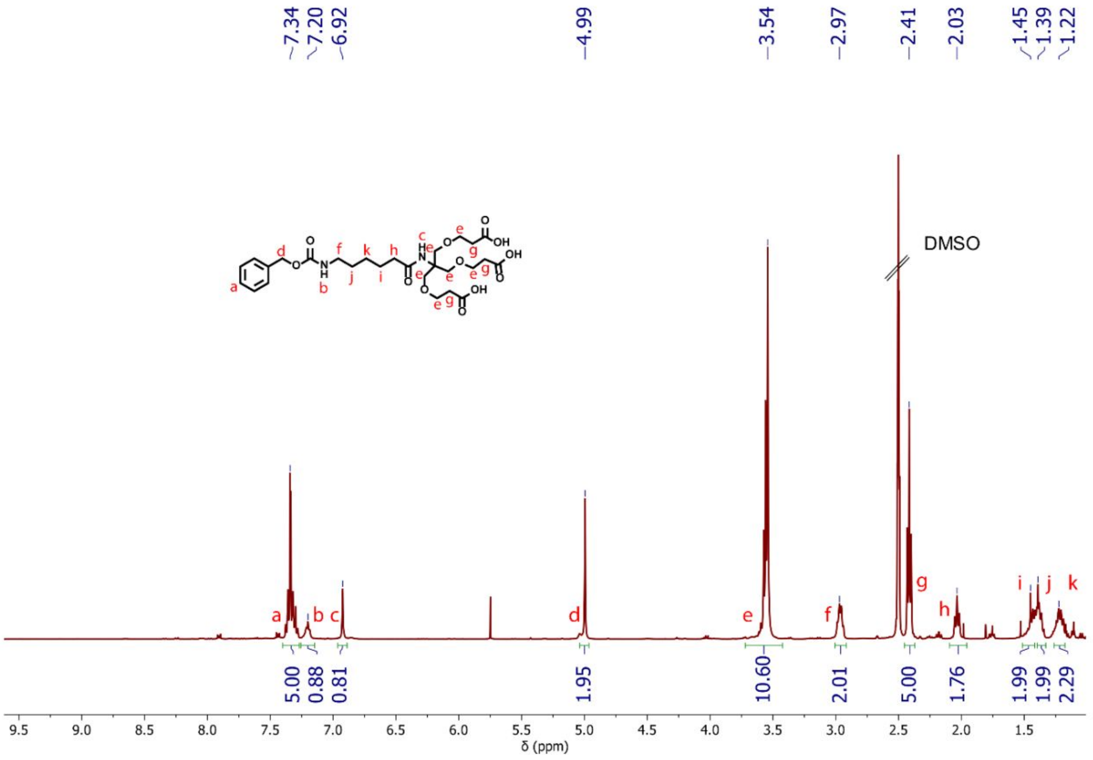

Figure S14. ^{1}H NMR spectrum of (iii) Cbz-Ahx-Tris[2-(carboxyethoxy)methyl]methylamide in DMSO-d_6.

Synthesis of Cbz-Ahx-Tris{[2-(N-Boc-ethylendiaminecarbonyl)ethoxy]methyl}methylamide (iiii)

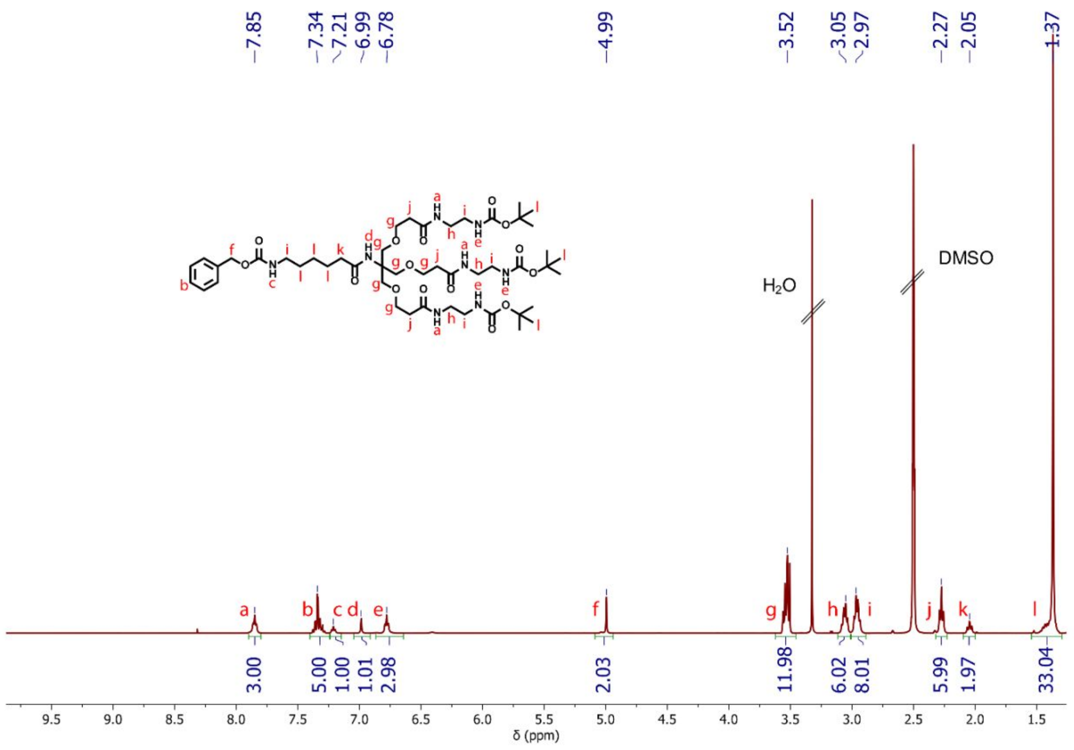

Figure S15. ^{1}H NMR spectrum of (iiii) Cbz-Ahx-Tris{[2-(*N*-Boc-ethylendiaminecarbonyl)ethoxy]methyl}methylamide in DMSO-d_6.

Boc-Deprotection of the tetrafunctional initiator (iiiii)

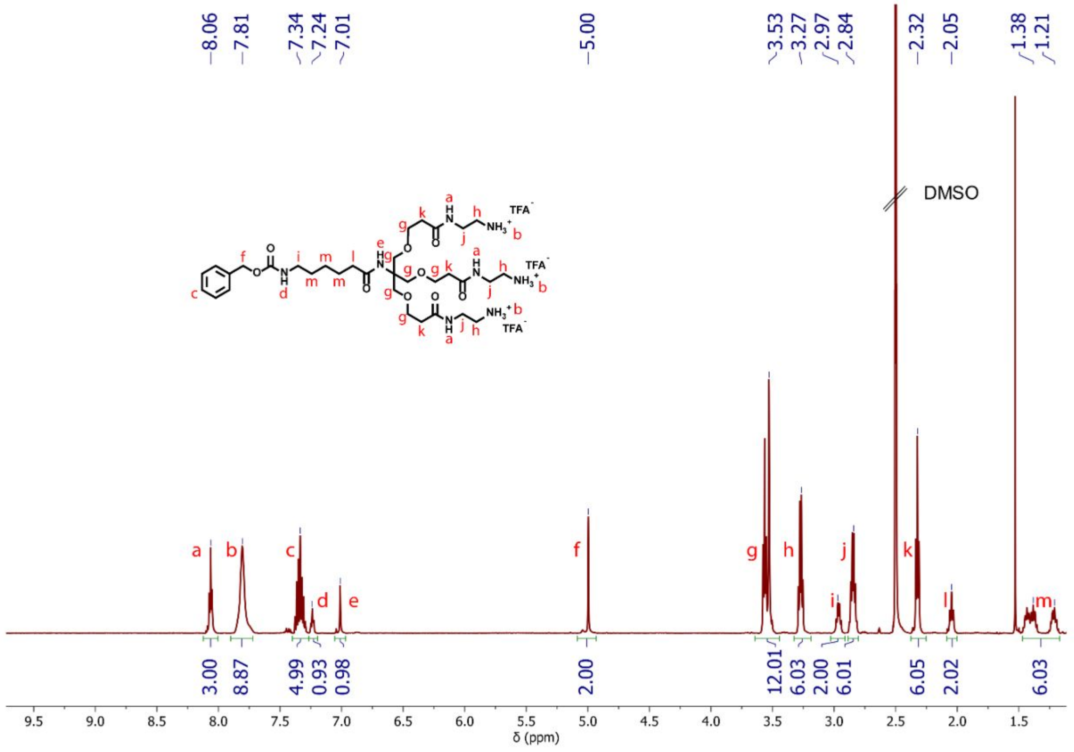

Figure S16. ^{1}H NMR spectrum of (iiiii) the Boc-deprotected tetrafunctional initiator in DMSO-*d*$_6$.

Synthesis of the heptafunctional initiator (iiiiii)

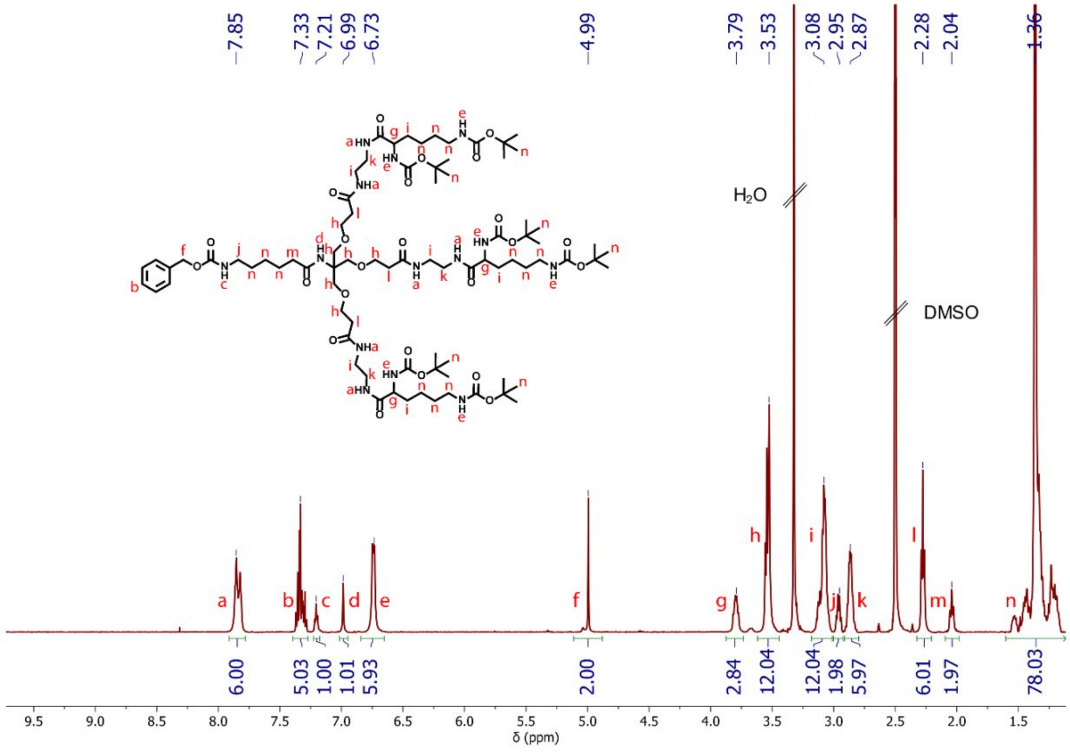

Figure S17. ^{1}H NMR spectrum of (iiiiii) the heptafunctional initiator in DMSO-d_6.

Initiator Deprotection for Macroinitiator Synthesis

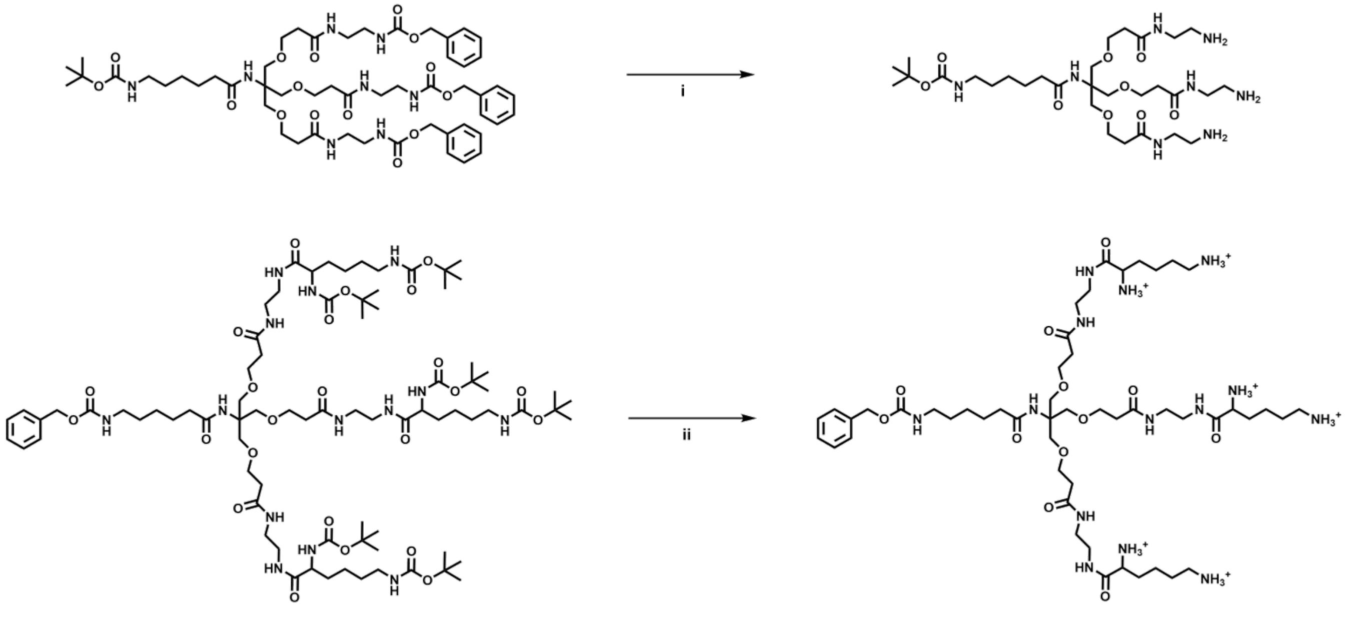

Figure S18. Applied deprotection methods as prerequisite for macroinitiator synthesis. i) Pd/C (10 wt%), H_2, MeOH, rt, 2d, yield: quantitative. ii) TFA/DCM (1/1), 0 °C, 1 h, yield: quantitative.

Cbz-deprotection of the tetrafunctional initiator (i)

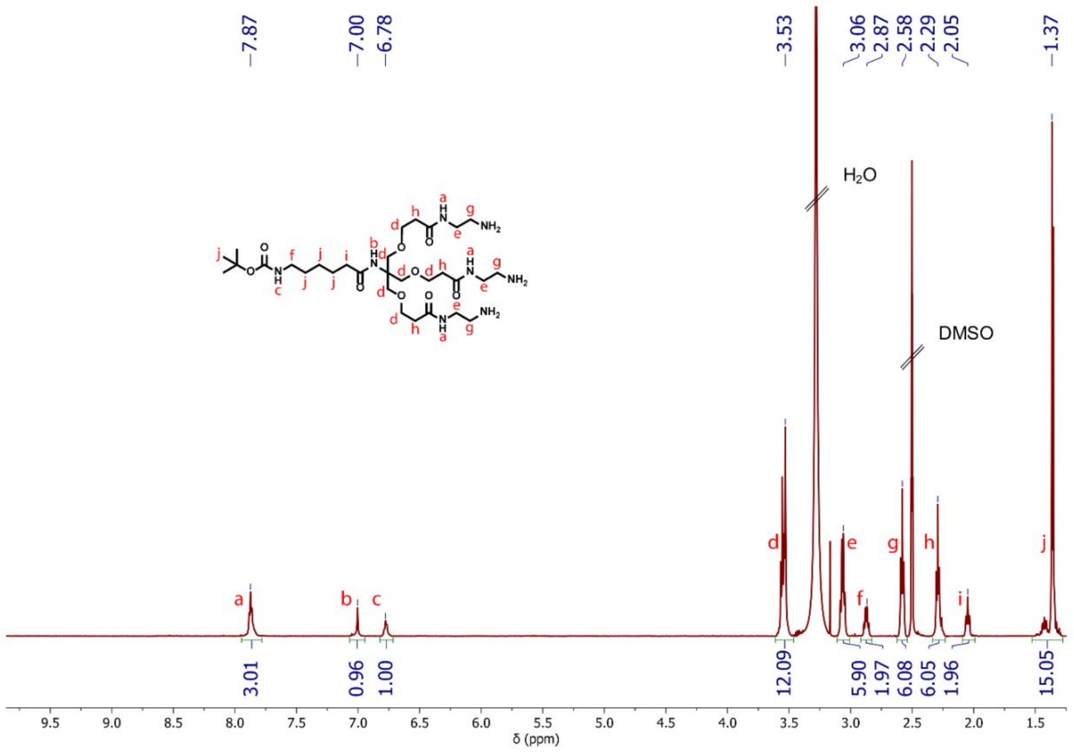

Figure S19. ^{1}H NMR spectrum of i) Cbz-deprotected tetrafunctional initiator in DMSO-d_6.

Boc-deprotection of the heptafunctional initiator (ii)

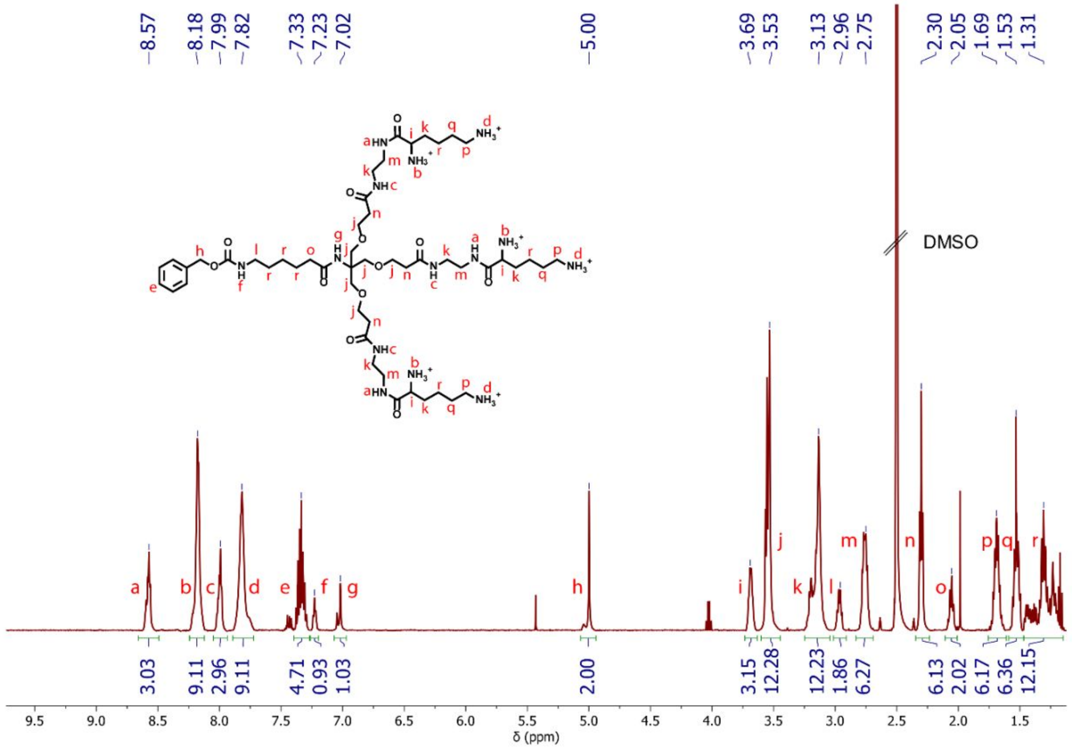

Figure S20. ^{1}H NMR spectrum of ii) Boc-deprotected heptafunctional initiator in DMSO-$\boldsymbol{d_6}$.

Polypeptoid Macroinitiators

Three-Arm Macroinitiator Synthesis (Boc-(pSar$_{100}$-N$_3$)$_3$)

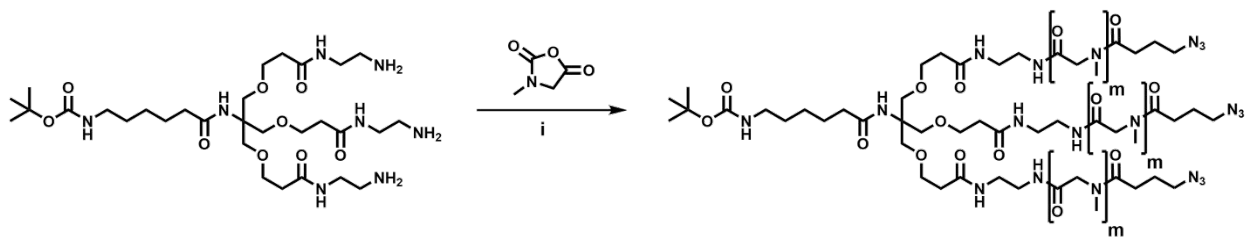

Figure S21. Synthesis of the three-arm macroinitiator with m = 100 i) DMF, 0 °C, 3d; pentafluorophenyl-4-azido-butanoat, DIPEA, rt, 1d, yield: 77 %.

Synthesis of three-arm pSar-macroinitiator (i)

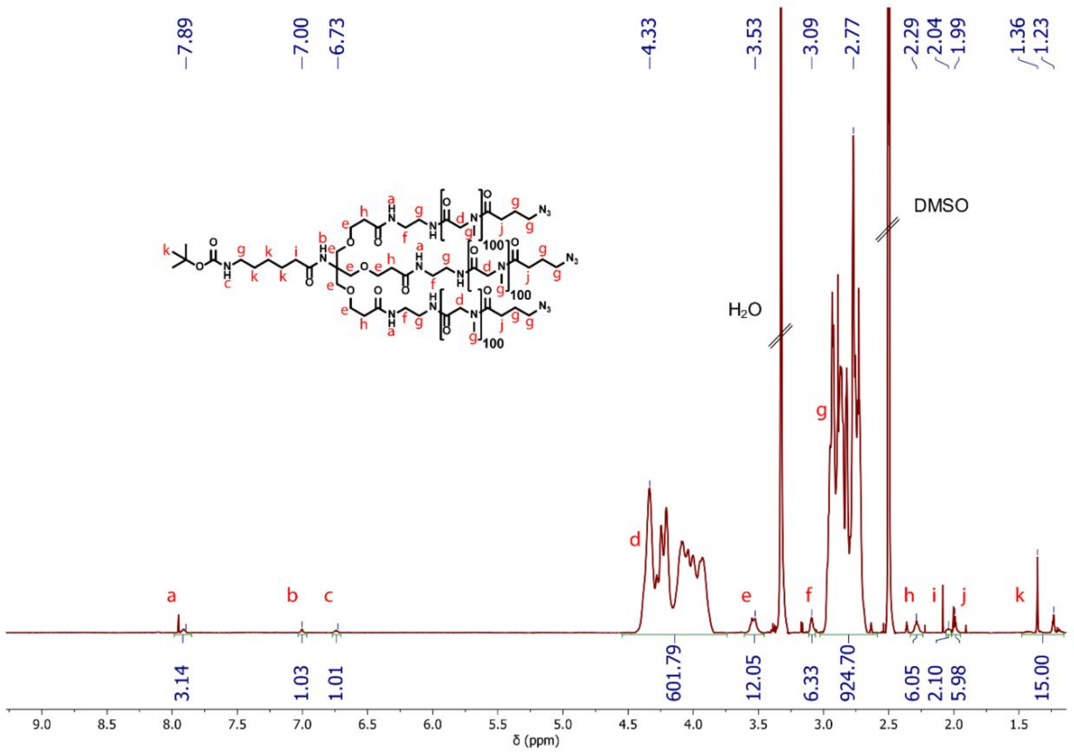

Figure S22. ^{1}H NMR spectrum of i) three-arm macroinitiator in DMSO-d_6.

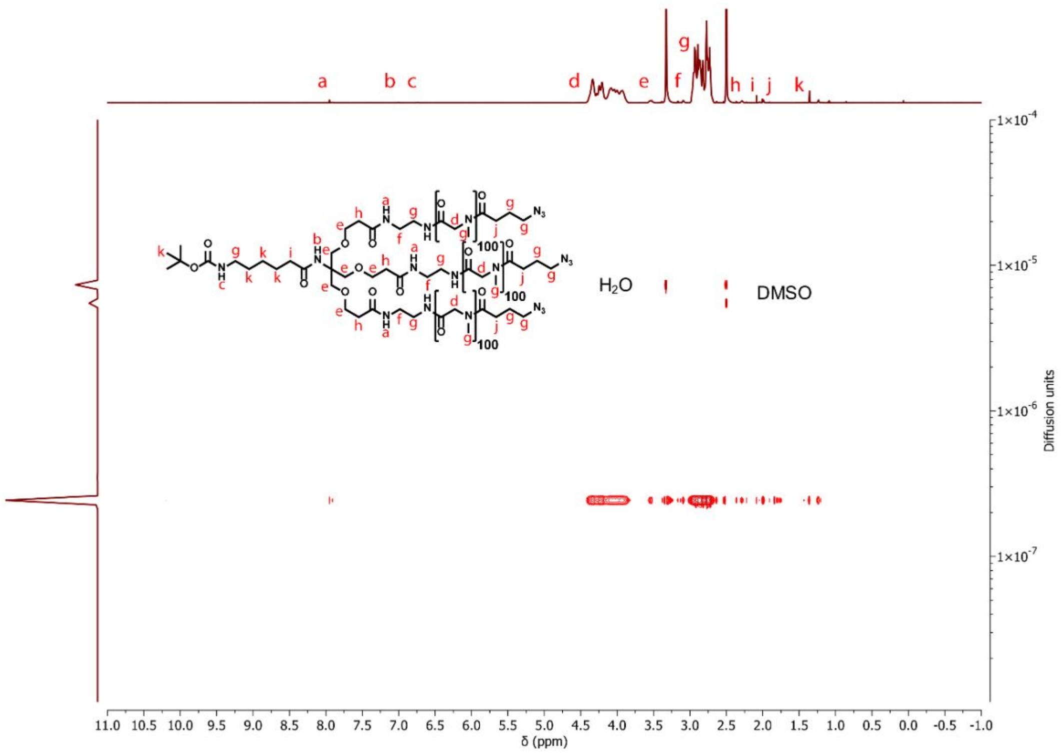

Figure S23. ^{1}H DOSY spectrum of i) three-arm macroinitiator in DMSO-d_6.

Six-Arm Macroinitiator Synthesis (Cbz-(pSar$_{50}$-N$_3$)$_6$)

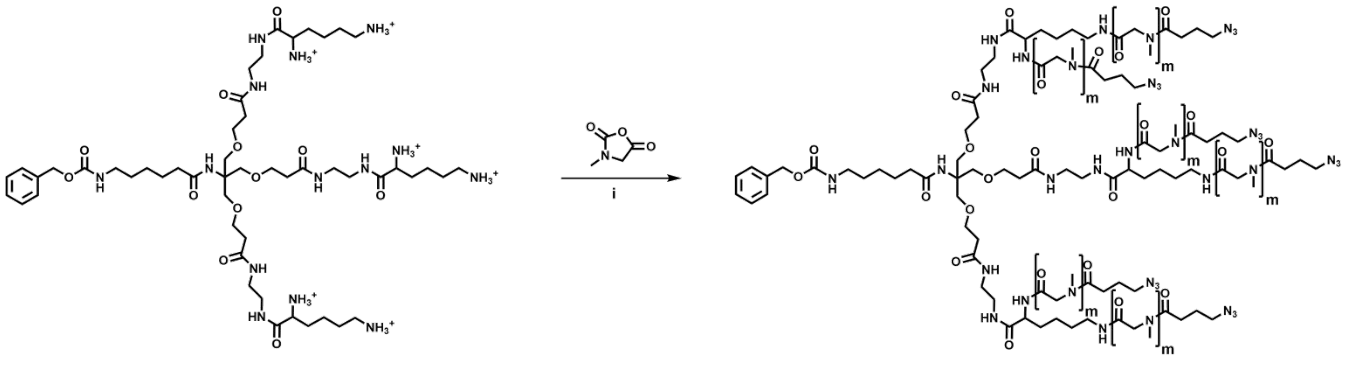

Figure S24. Synthesis of the six-arm macroinitiators with m = 50 i) DMF, DIPEA, 0 °C, 3d; pentafluorophenyl-4-azido-butanoat, DIPEA, rt, 1d, yield: 72 %.

Synthesis of six-arm pSar-macroinitiator (i)

Boc-Deprotection of the Three-Arm Macroinitiator (NH_2-($pSar_{100}$-N_3)$_3$)

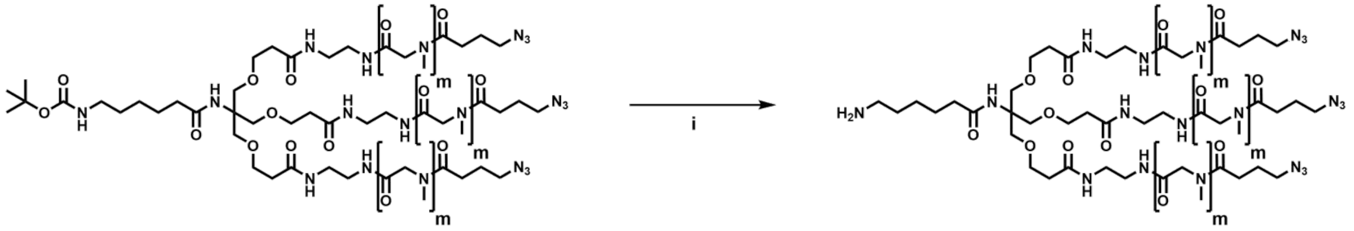

Figure S25. Boc-deprotection of the three-arm macroinitiator i) TFA/H_2O (1/1), 0 °C, 2 h, yield: 65%.

Boc-deprotection of three-arm macroinitiator Boc-($pSar_{100}$-N_3)$_3$ (i)

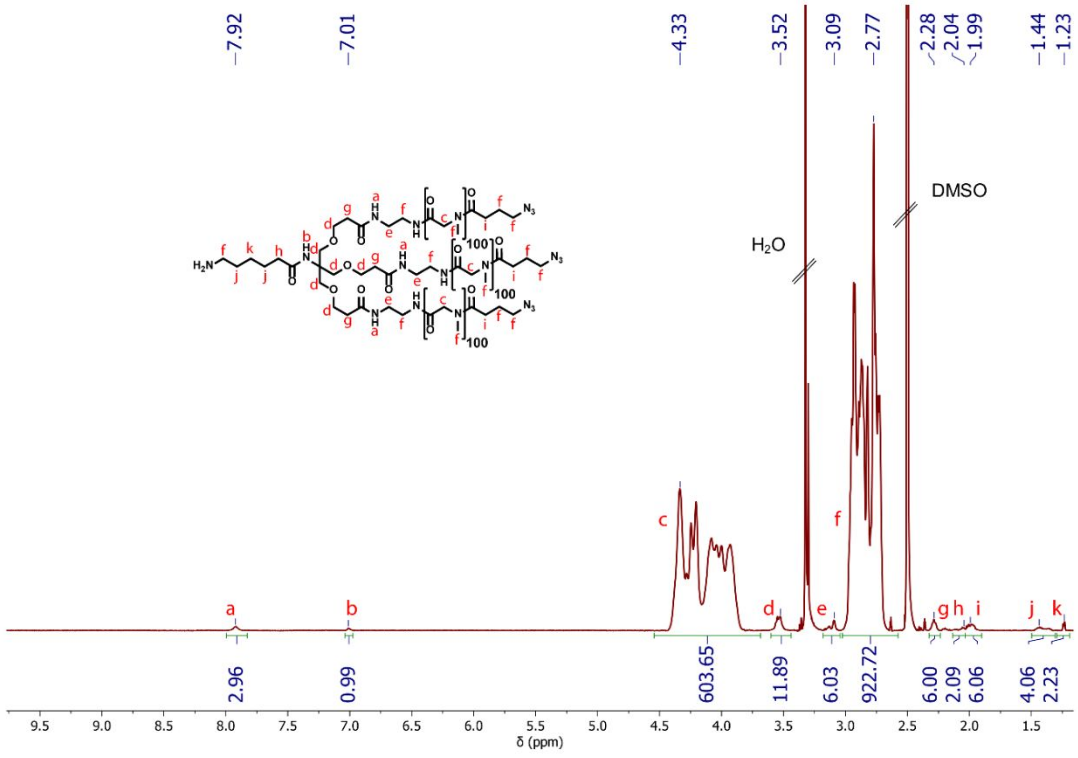

Figure S26. ^{1}H NMR spectrum of i) deprotected three-arm macroinitiator in DMSO-d_6.

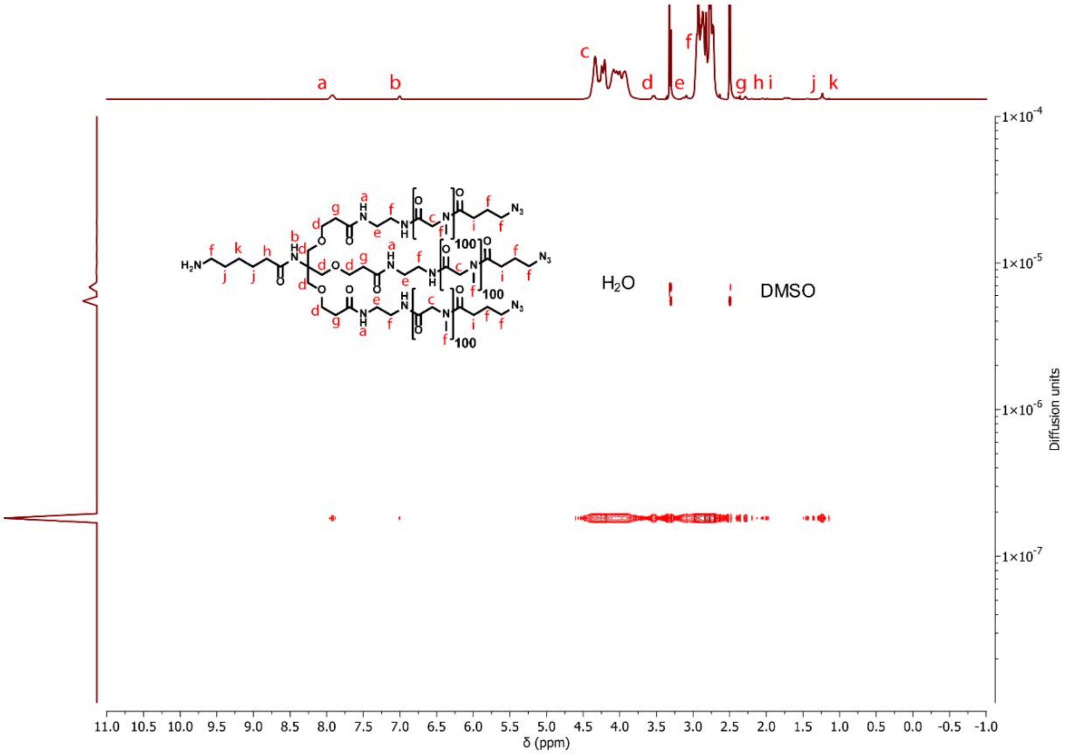

Figure S27. DOSY spectrum of i) deprotected three-arm macroinitiator in DMSO-d_6.

Cbz-Deprotection of the Six-Arm Macroinitiator (NH_2-($pSar_{50}$-N_3)$_6$)

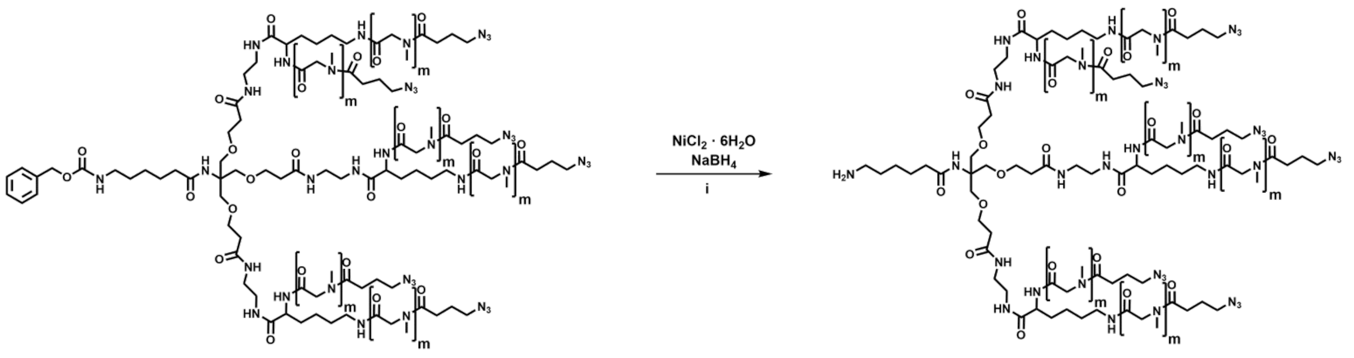

Figure S28. Cbz-deprotection of the six-arm macroinitiator i) MeOH, 0 °C, 1 h, rt, 48 h yield: 63%.

Cbz-deprotection of six-arm macroinitiator Cbz-($pSar_{50}$-N_3)$_6$ (i)

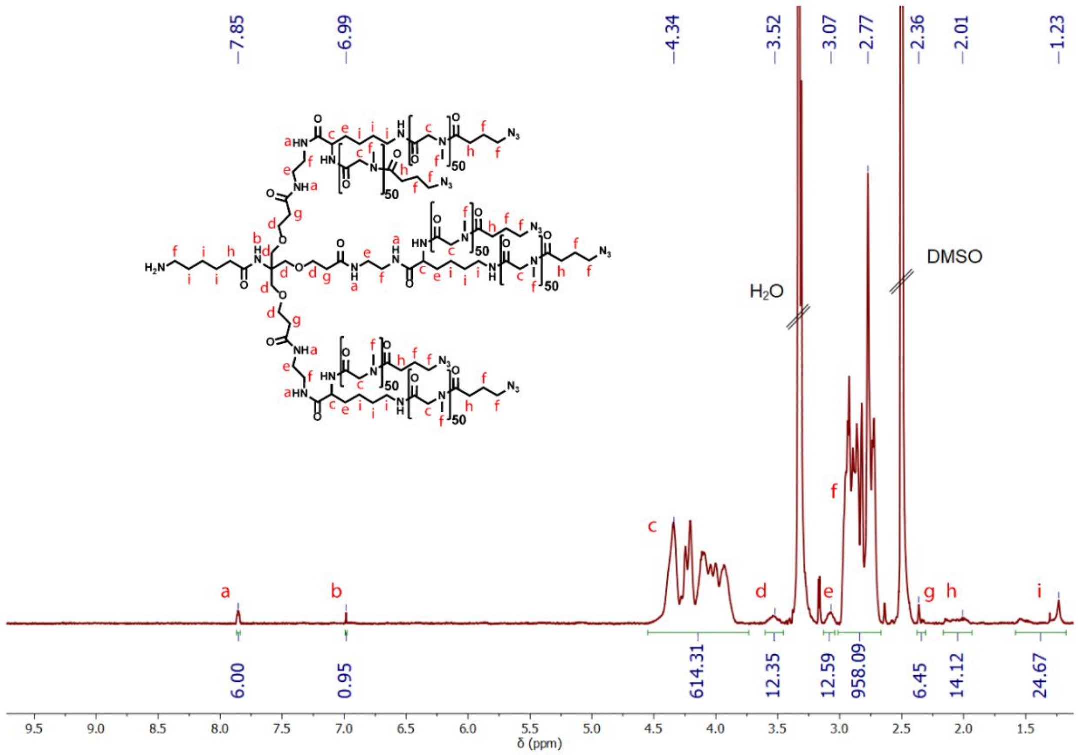

Figure S29. ^{1}H NMR spectrum of i) deprotected six-arm macroinitiator in DMSO-d_6.

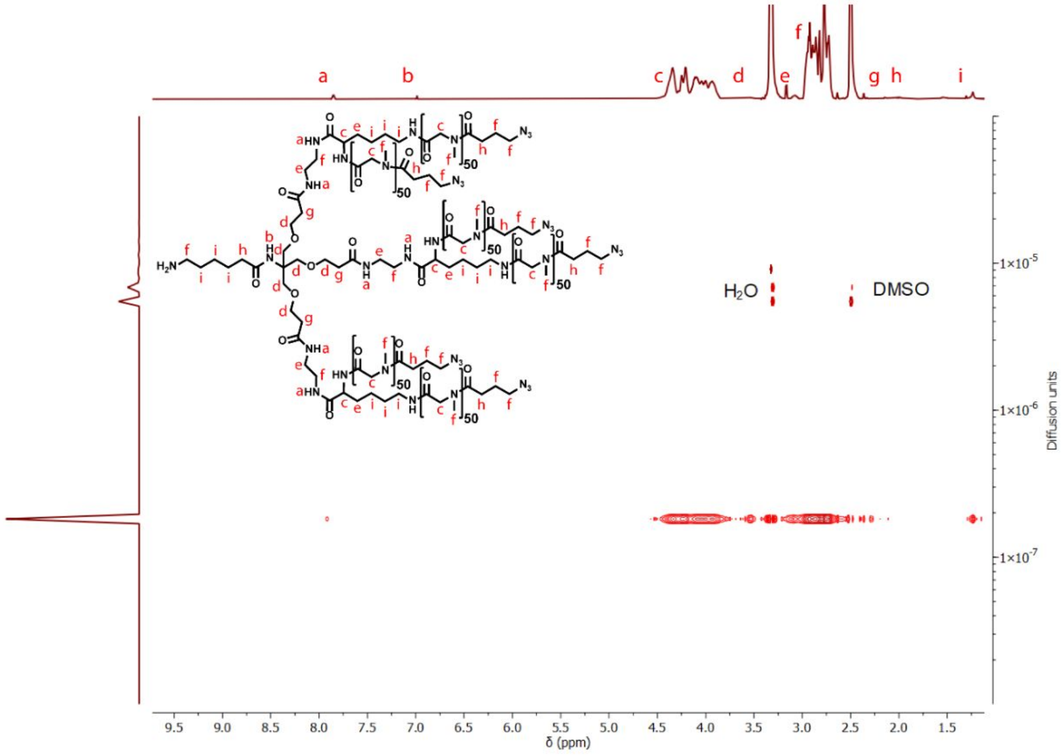

Figure S30. ^{1}H NMR spectrum of i) deprotected six-arm macroinitiator in DMSO-d_6.

Cross-linkable and Complexing Miktoarm Star Polymers (PeptoMiktoStars)

AB_3 PeptoMiktoStar ($pLys(Boc)_{20}pHcy(SO_2Et)_{20}(pSar_{100}\text{-}N_3)_3$)

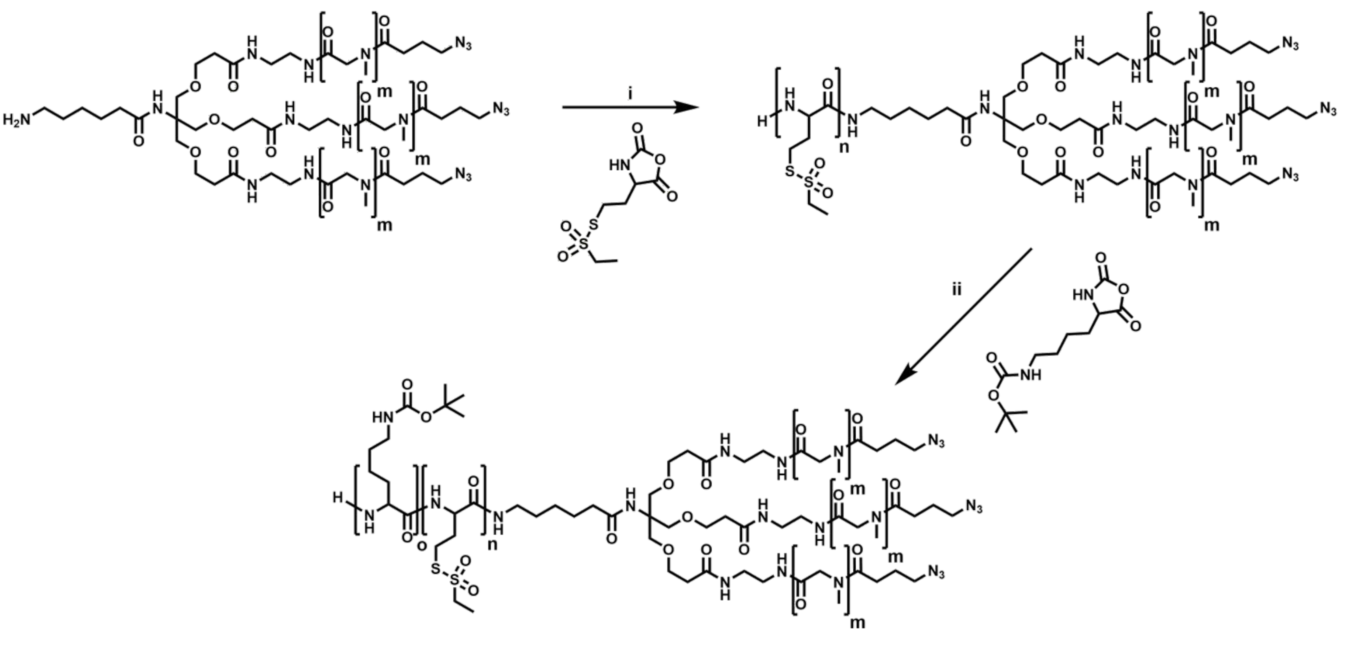

Figure S31. Introduction of both polypeptide blocks for the synthesis of AB_3 PeptoMiktoStars with m = 100, n = 20 and o = 20, i) DMF, -10 °C, 7 d, yield: 78%; ii) DMF, 0 °C, 3 d, yield: 80 %.

Introduction of the pHcy(SO_2Et)-block - (pHcy(SO_2Et)$_{20}$(pSar$_{100}$-N_3)$_3$ (i)

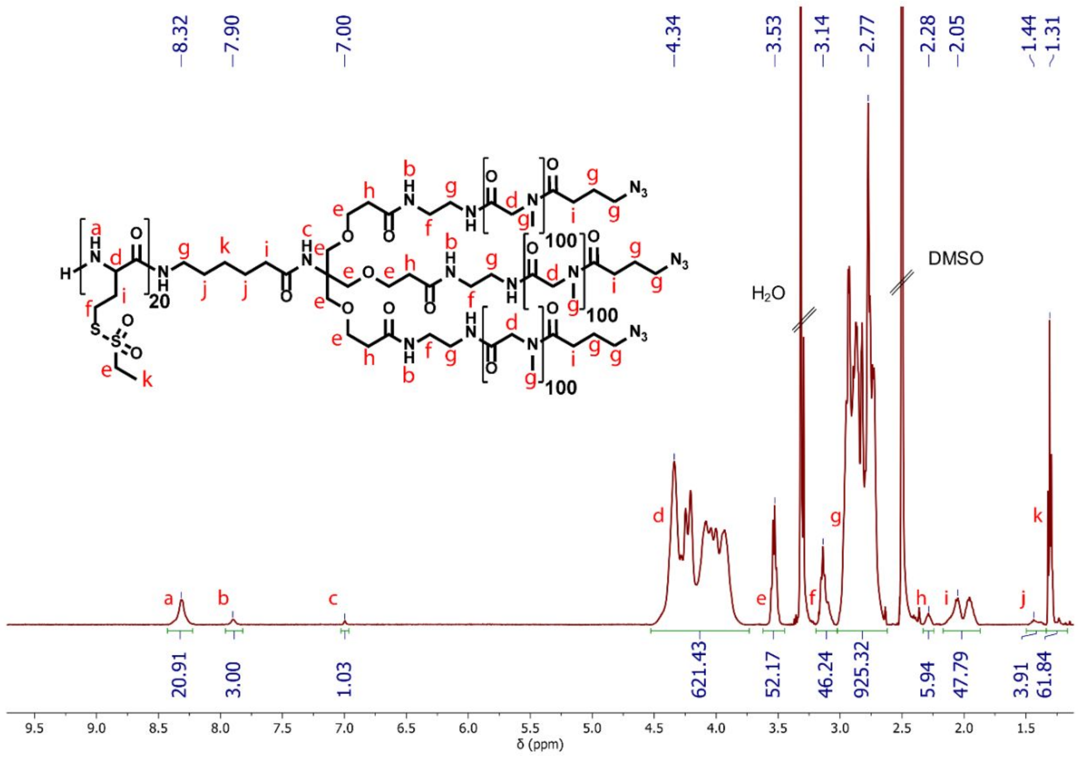

Figure S32. ^{1}H NMR spectrum of i) AB_3 PeptoMiktoStar pHcy(SO_2Et)$_{20}$(pSar$_{100}$-N_3)$_3$ in DMSO-d_6.

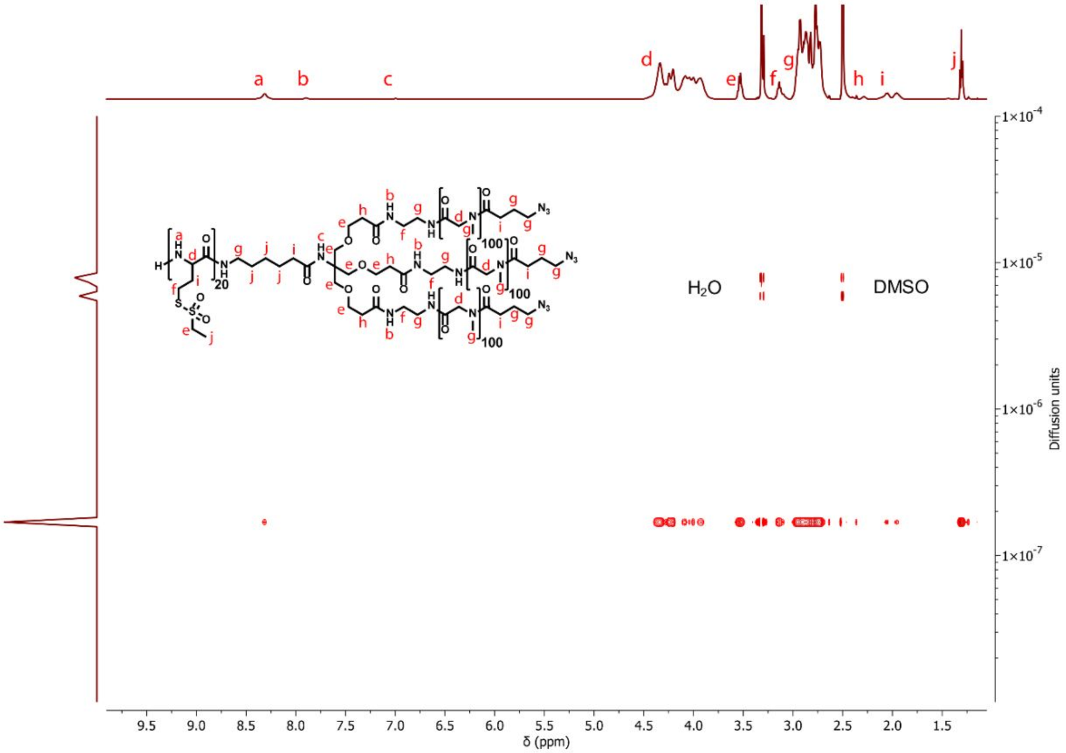

Figure S33. DOSY spectrum of i) AB_3 PeptoMiktoStar pHcy$(SO_2Et)_{20}$(pSar$_{100}$-N_3)$_3$ in DMSO-*d*$_6$.

Introduction of the pLys(Boc)-block - (pLys(Boc)$_{20}$pHcy(SO$_2$Et)$_{20}$(pSar$_{100}$-N$_3$)$_3$ (ii)

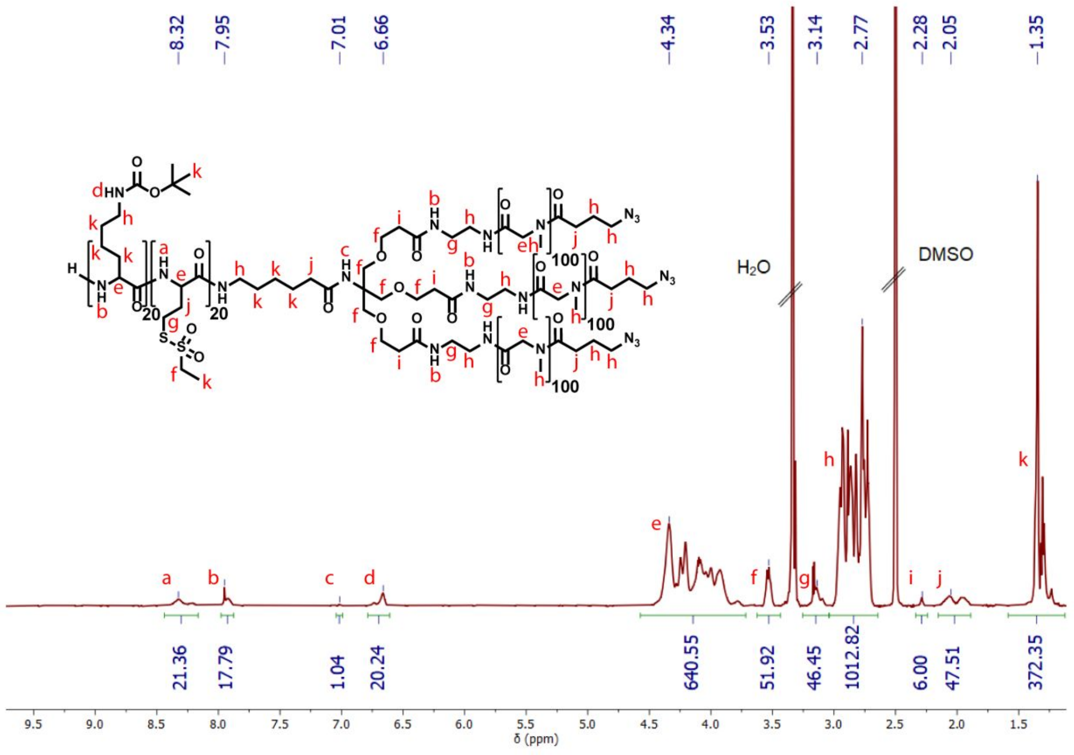

Figure S34. ^{1}H NMR spectrum of ii) AB$_3$ PeptoMiktoStar pLys(Boc)$_{20}$pHcy(SO$_2$Et)$_{20}$(pSar$_{100}$-N$_3$)$_3$ in DMSO-d_6.

Deprotected AB$_3$ PeptoMiktoStar (pLys$_{20}$pHcy(SO$_2$Et)$_{20}$(pSar$_{100}$-N$_3$)$_3$)

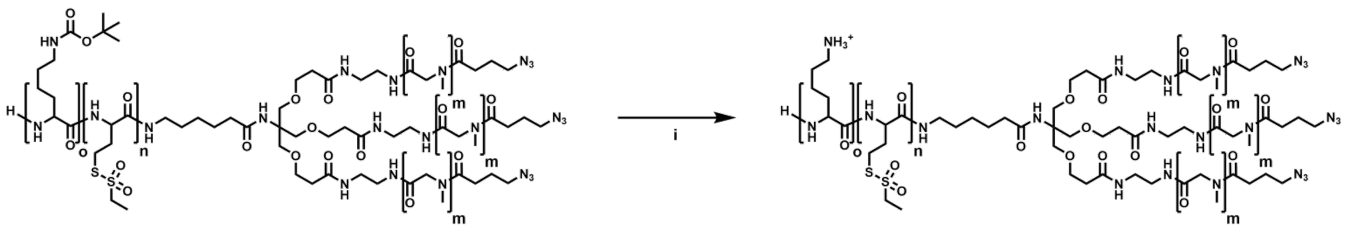

Figure S35. Boc-Deprotection of AB$_3$ PeptoMiktoStars with m = 100, n = 20 and o = 20 i) TFA/H_2O (1/1), 2h, 0 °C, overnight, rt, yield: 85%.

AB_6 PeptoMiktoStar ($pLys(Boc)_{20}pHcy(SO_2Et)_{20}(pSar_{50}\text{-}N_3)_6$)

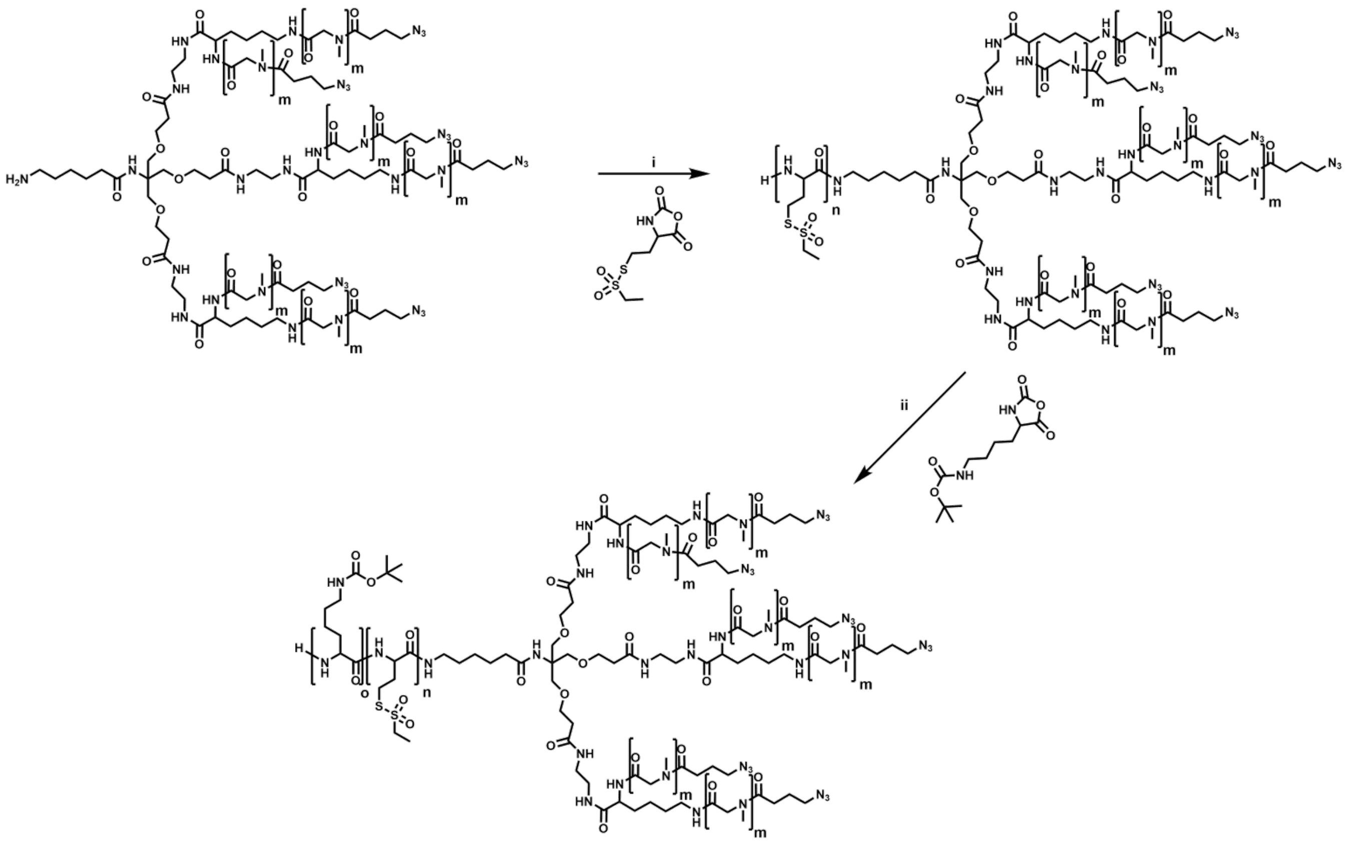

Figure S36. Introduction of both polypeptide blocks for the synthesis of AB_6 PeptoMiktoStars with m = 50, n = 20 and o = 20, i) DMF, -10 °C, 7 d, yield: 67%; ii) DMF, 0 °C, 3 d, yield: 72%.

Introduction of the pHcy(SO_2Et)-block - (pHcy(SO_2Et)$_{20}$(pSar$_{50}$-N_3)$_6$ (i)

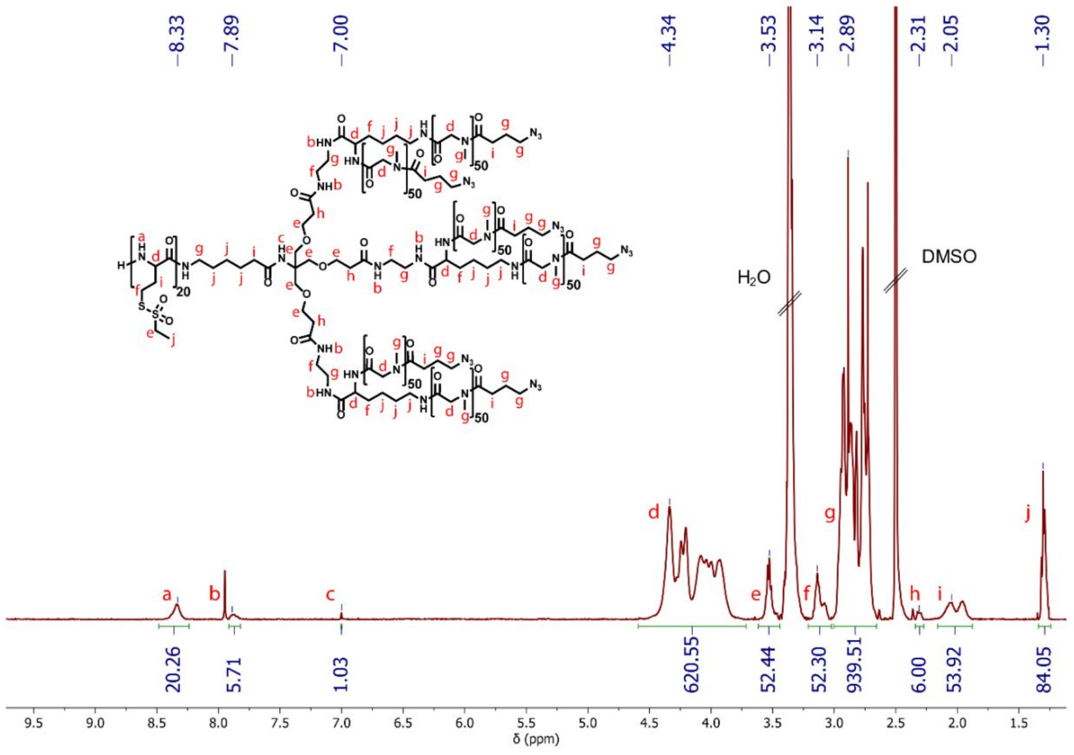

Figure S37. ^{1}H NMR spectrum of i) AB$_6$ PeptoMiktoStar pHcy(SO_2Et)$_{20}$(pSar$_{50}$-N_3)$_6$ in DMSO-*d*$_6$.

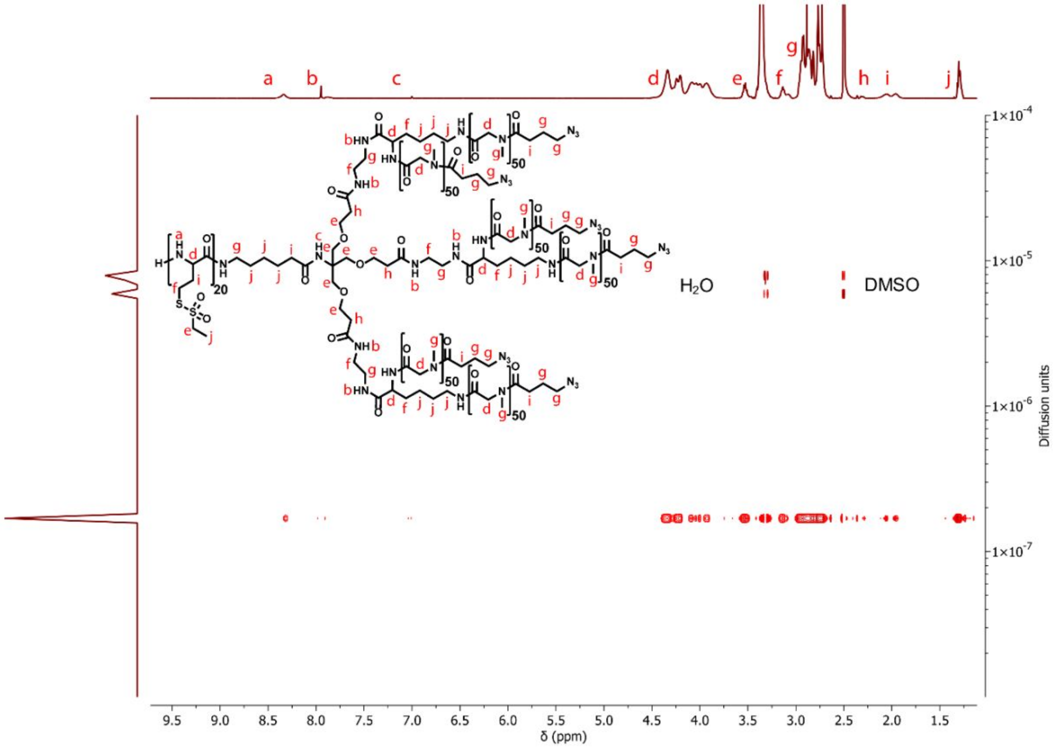

Figure S38. DOSY spectrum of i) AB_6 PeptoMiktoStar $pHcy(SO_2Et)_{20}(pSar_{50}\text{-}N_3)_6$ in DMSO-d_6.

Introduction of the pLys(Boc)-block - (pLys(Boc)$_{20}$pHcy(SO$_2$Et)$_{20}$(pSar$_{50}$-N$_3$)$_6$ (ii)

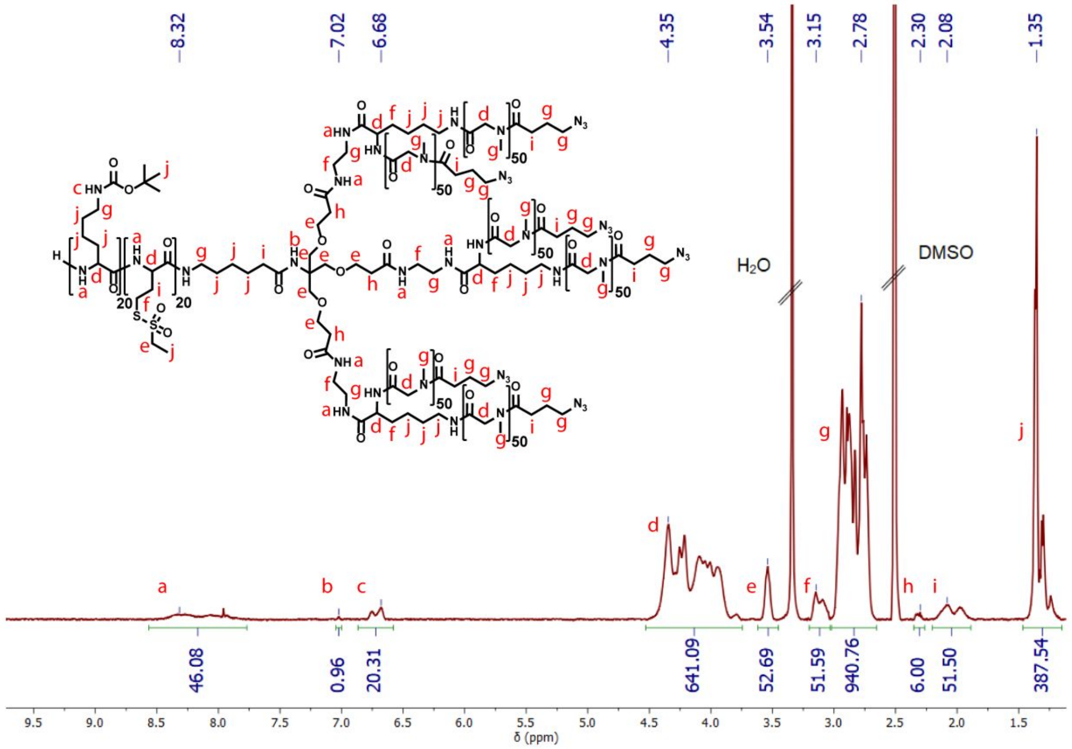

Figure S39. ^{1}H NMR spectrum of ii) AB$_6$ PeptoMiktoStar pLys(Boc)$_{20}$pHcy(SO$_2$Et)$_{20}$(pSar$_{50}$-N$_3$)$_6$ in DMSO-d_6.

Deprotected AB_6 PeptoMiktoStar ($pLys_{20}pHcy(SO_2Et)_{20}(pSar_{50}\text{-}N_3)_6$)

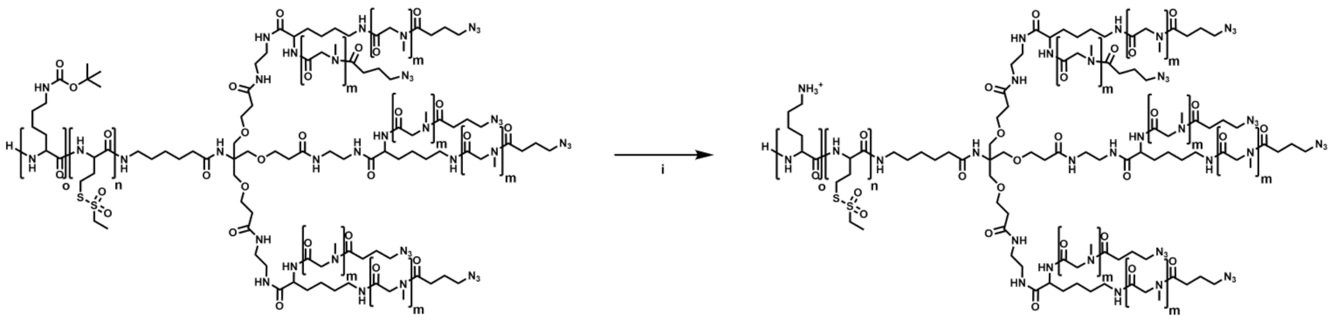

Figure S40. Boc-Deprotection of AB_6 PeptoMiktoStars with m = 50, n = 20 and o = 20 i) TFA/H_2O (1/1), 2h, 0 °C, overnight, rt, yield: 77 %.

Further Reagents

Pentafluorophenyl-4-azidobutanoat

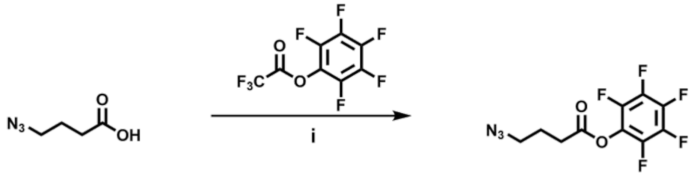

Figure S41. Synthesis of the azide functionalization agent pentafluorophenyl-4-azidobutanoat.(i) TEA, THF, rt, 1d, yield: 83%.

Synthesis of Pentafluorophenyl-4-azidobutanoat (i)

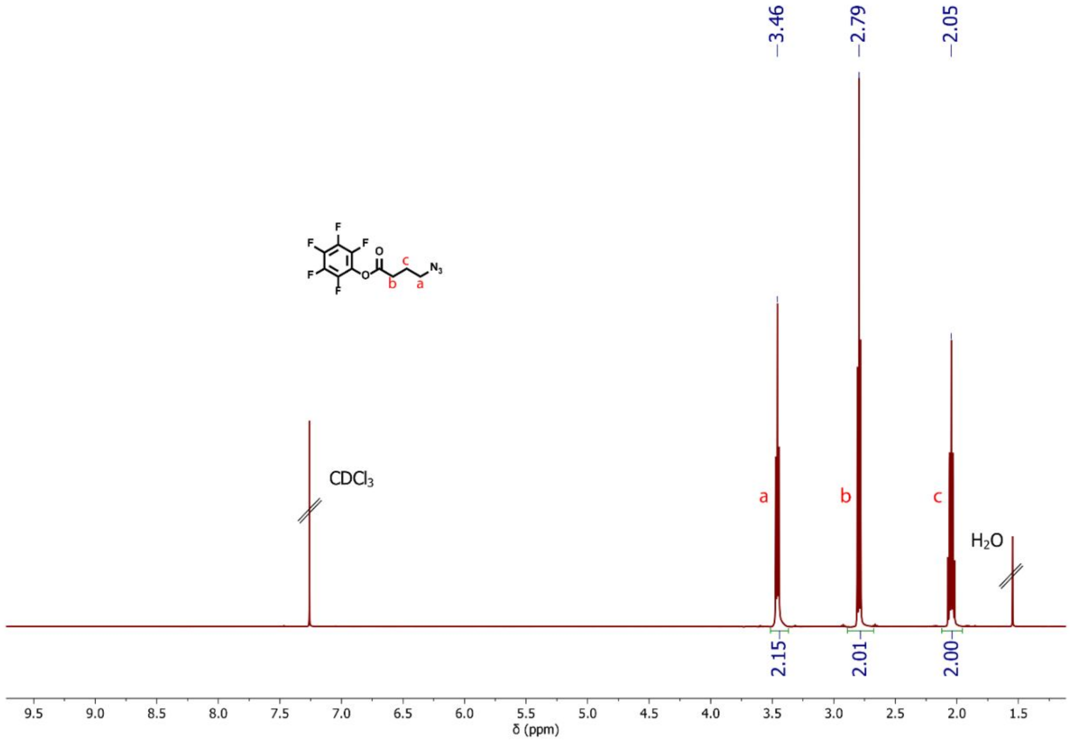

Figure S42. ^{1}H NMR spectrum of pentafluorophenyl-4-azidobutanoat in $CDCl_3$.

Biological test

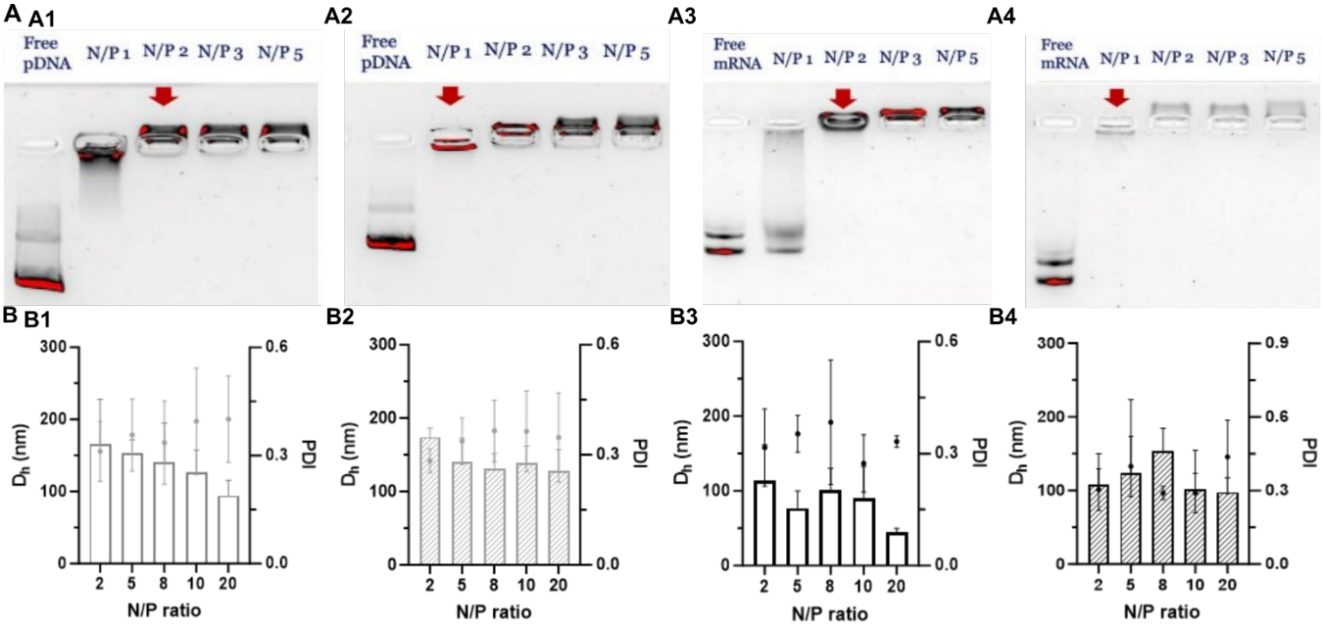

Figure S43. Characterization of pDNA/ABn and mRNA/ABn PICMs. (A) Complexation ability of (A1, A2) pDNA and (A3, A4) mRNA by (A1, A3) AB3 or (A2, A4) AB6 triblock copolymer using agarose gel electrophoresis. Lane 1, naked/free (A1, A2) pDNA or (A3, A4) mRNA, lane 2-5, (A1, A2) pDNA/ABn PICMs at N/P 1, 2, 3 and 5, (A3, A4) mRNA/ABn PICMs at N/P 1, 2, 3 and 5. (B) Size and size distribution of (B1, B2) pDNA/ABn PICMs and (B3, B4) mRNA/ABn PICMs complexed by triblock copolymer of (B1, B3) AB_3 or (B2, B4) AB_6 in HEPES buffer (10 mM, pH7.4) measured by DLS. All data was averaged from three independent experiments (n≥9).

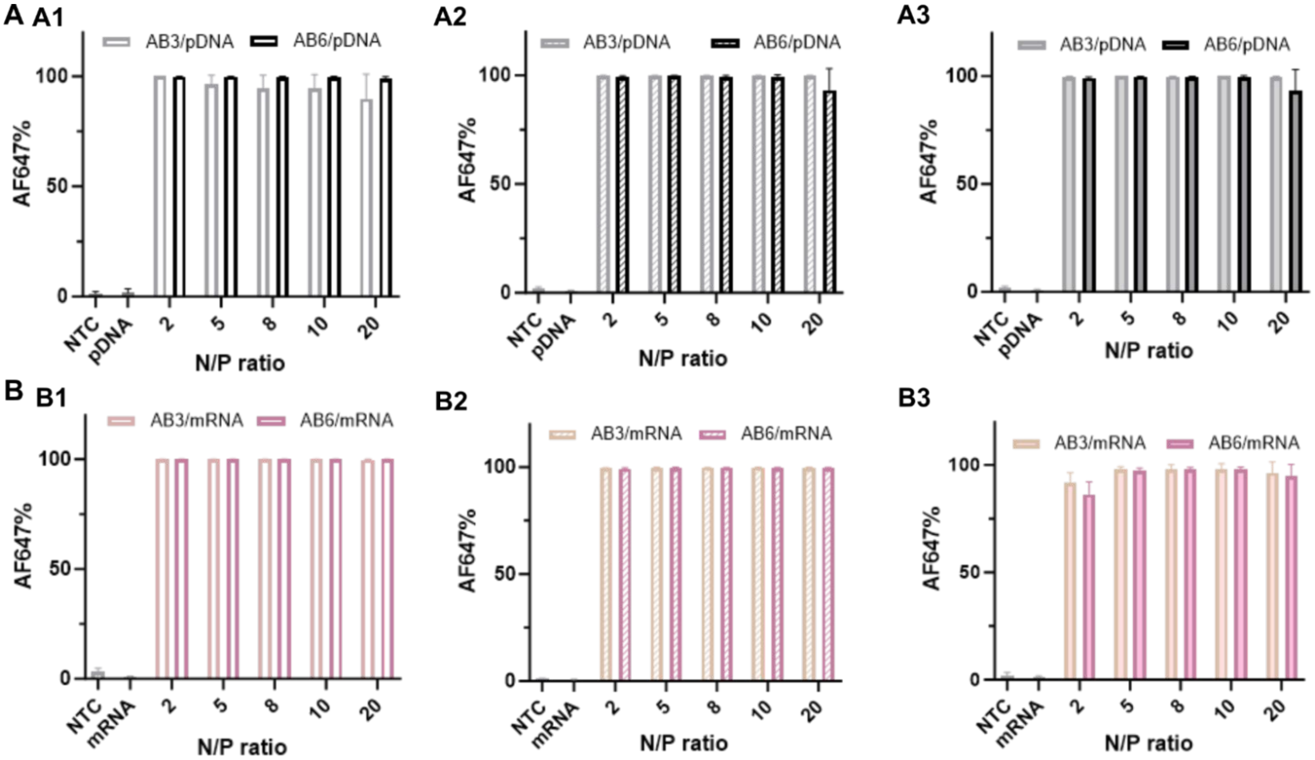

Figure S44. *In vitro* evaluation of (A) pDNA/AB_n PICMs and (B) mRNA/ABn PICMs on (A1, B1) D1 cells and (A2, B2, A3, B3) Jurkat T cells. (A1) D1 cells and (A2) Jurkat T cells were exposed to pDNA/AB_n PICMs (final pDNA concentration of 0.2 µg/well) in fresh culture medium for 48 h. (A3) pDNA/ABn PICMs were incubated with Jurkat T cells in Opti-MEM for 48 h. At the end of incubation, cells were collected for flow cytometry measurements. (B1) D1 cells and (B2) Jurkat T cells were exposed to mRNA/ABn PICMs in fresh culture medium for 24 h. (B3) mRNA/AB_n PICMs were incubated with Jurkat T cells in Opti-MEM for 24 h. At the end of incubation, cells were collected for flow cytometry measurements. In each well, the final pDNA or mRNA concentration is 0.2 µg/well. All data was averaged from three independent experiments (n≥9).

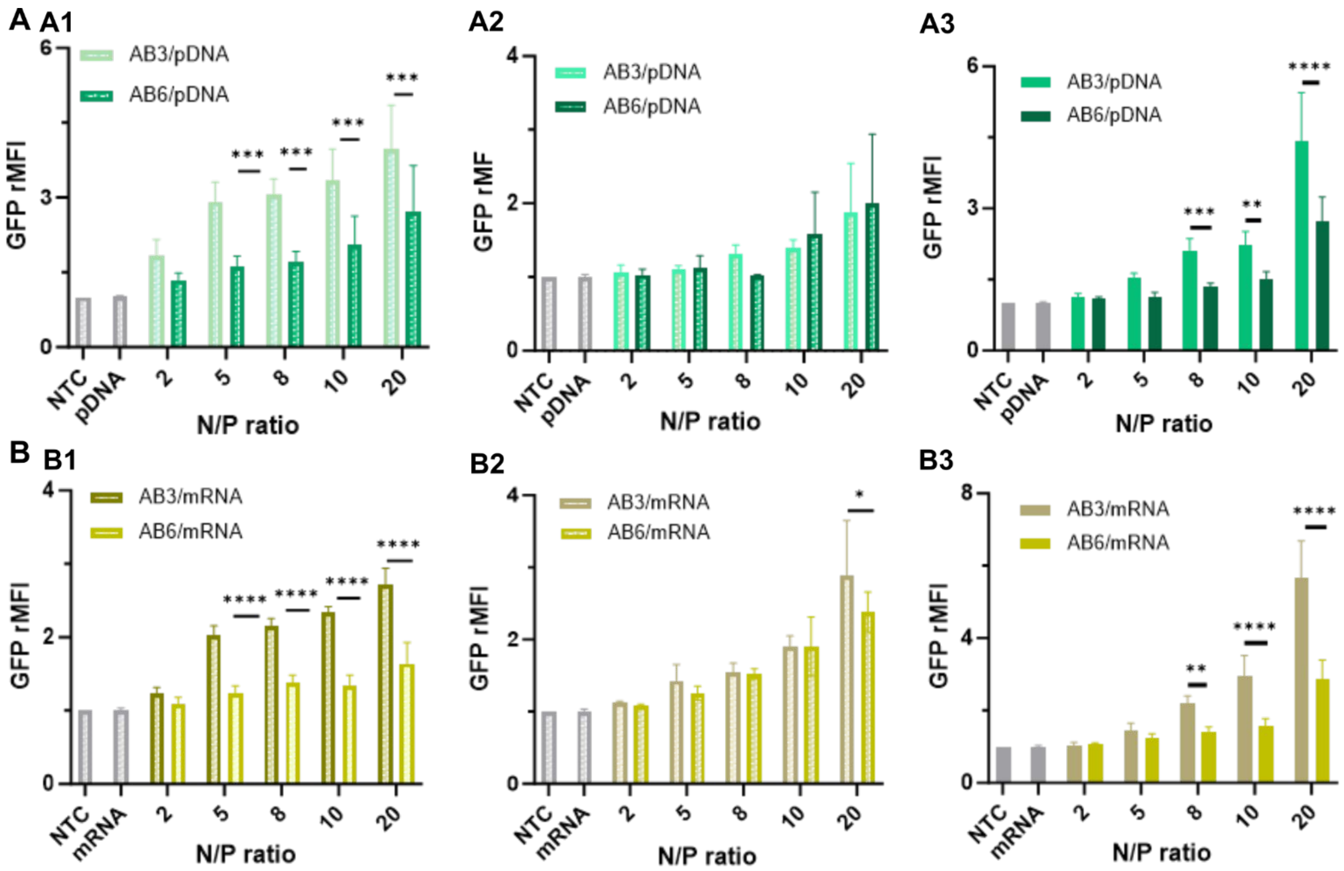

Figure S45. *In vitro* evaluation of (A) pDNA/AB$_n$ PICMs and (B) mRNA/AB$_n$ PICMs on (A1, B1) D1 cells and (A2, B2, A3, B3) Jurkat T cells. (A1) D1 cells and (A2) Jurkat T cells were exposed to pDNA/AB$_n$ PICMs (final pDNA concentration of 0.2 μg/well) in fresh culture medium for 48 h. (A3) pDNA/ABn PICMs were incubated with Jurkat T cells in Opti-MEM for 48 h. At the end of incubation, cells were collected for flow cytometry measurements. (B1) D1 cells and (B2) Jurkat T cells were exposed to mRNA/AB$_n$ PICMs in fresh culture medium for 24 h. (B3) mRNA/AB$_n$ PICMs were incubated with Jurkat T cells in Opti-MEM for 24 h. At the end of incubation, cells were collected for flow cytometry measurements. In each well, the final pDNA or mRNA concentration is 0.2 μg/well. All data was averaged from three independent experiments (n≥9).

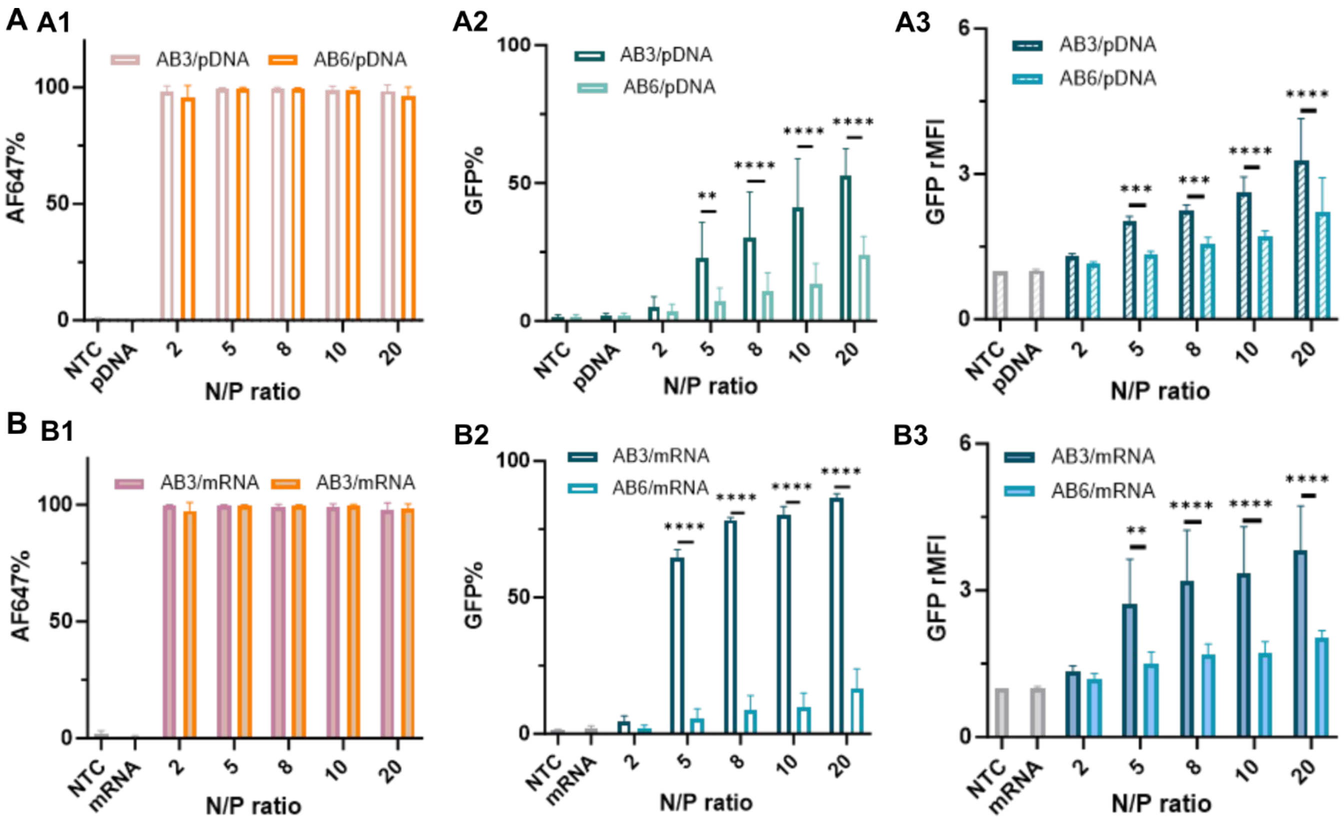

Figure S46. *In vitro* evaluation of (A) pDNA/AB_n PICMs and (B) mRNA/AB_n PICMs on D1 cells. (A) D1 cells cultured in Opti-MEM were exposed to pDNA/ABn PICMs (A1) for 24 h to examine cellular uptake and (A2, A3) for 48 h to examine transfection efficiency (final pDNA concentration of 0.2 µg/well). (B) D1 cells cultured in Opti-MEM were exposed to mRNA/ABn PICMs for 24 h (B1) for cellular uptake and (B2, B3) for transfection efficiency (final mRNA concentration of 0.2 µg/well). At the end of incubation, cells were collected for flow cytometry measurements. All data was averaged from three independent experiments (n≥9).

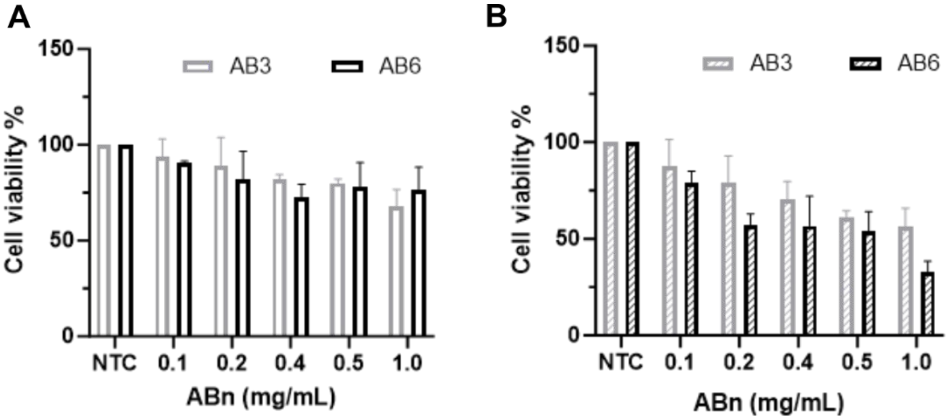

Figure S47. Cell viability of (A) D1 cell and (B) Jurkat T cell upon 24 hours' incubation with blank AB3 and blank AB6 at a final concentration of 0.1, 0.2, 0.4, 0.5 and 1.0 mg/mL. All data was averaged from three independent experiments (n≥9).
